# Immunotherapy, prognostic, and tumor biomarker based on pancancer analysis, SMARCD3

**DOI:** 10.18632/aging.205921

**Published:** 2024-06-11

**Authors:** Zishun Guo, Bingji Cao, Zhuozheng Hu, Jiajun Wu, Weijun Zhou, Wenxiong Zhang, Zhihua Shi

**Affiliations:** 1 Department of Thoracic Surgery, The Second Affiliated Hospital of Nanchang University, Nanchang 330006, China; 2 Department of Thoracic Surgery, The Fourth Hospital of Hebei Medical University, Shijiazhuang 050011, China

**Keywords:** SMARCD3, pan-cancer analysis, immunotherapy, prognostic, biomarker

## Abstract

Background: SMARCD3 has recently been shown to be an important gene affecting cancer, playing an important role in medulloblastoma and pancreatic ductal adenocarcinoma. Therefore, we conducted this research to investigate the potential involvement of SMARCD3 across cancers and to offer recommendations for future studies.

Methods: Utilizing information on 33 malignancies in the UCSC Xena database, SMARCD3 expression and its prognostic value were assessed. The tumor microenvironment was evaluated with the “CIBERSORT” and “ESTIMATE” algorithms. SMARCD3 and immune-related genes were analyzed using the TISIDB website. The pathways related to the target genes were examined using GSEA. MSI (microsatellite instability), TMB (tumor mutational burden), and immunotherapy analysis were used to evaluate the impact of target genes on the response to immunotherapy.

Results: There is heterogeneity in terms of the expression and prognostic value of SMARCD3 among various cancers, but it is a risk factor for many cancers including uterine corpus endometrial cancer (UCEC), renal clear cell carcinoma (KIRC), and gastric adenocarcinoma (STAD). GSEA revealed that SMARCD3 is related to chromatin remodeling and transcriptional activation, lipid metabolism, and the activities of various immune cells. The TMB and MSI analyses suggested that SMARCD3 affects the immune response efficiency of KIRC, LUAD and STAD. Immunotherapy analysis suggested that SMARCD3 may be a potential immunotherapy target. RT-qPCR demonstrated the variation in SMARCD3 expression in KIRC, LUAD, and STAD.

Conclusion: Our study revealed that SMARCD3 affects the prognosis and immunotherapy response of some tumors, providing a direction for further research on this gene.

## INTRODUCTION

Every year, cancer morbidity and death rates increase worldwide, resulting in significant social and economic burdens [1–3]. In the exploration of cancer treatment methods, the exploration of new tumor markers and targets is becoming increasingly important, and precise treatment strategies targeting tumor markers have become an important research direction for cancer treatment [4, 5].

SMARCD3 (SWI/SNF connected, matrix related, actin dependent regulator of chromatin, subfamily d, member 3) is a gene with great potential value. It has been demonstrated to have a significant impact on chromatin remodeling, DNA damage repair, and other processes. When SMARCD3 is silenced in mice, it leads to abnormalities in heart development [6]. SMARCD3 genetic polymorphisms are strongly linked to DNA damage levels in the Chinese population [7].

Recently, researchers have discovered that it also plays a role in cancer. SMARCD3 was shown to control epithelial–mesenchymal transition in breast cancer cells and epithelial tissue stem cells [8]. The urothelial carcinoma associated 1 (UCA1)/SMARCD3 axis promotes cervical cancer development [9]. The SMARCD protein plays an important role in prostate cancer [10]. In recent research, high SMARCD3 expression has been shown to be closely related to medulloblastoma metastasis [11]. In pancreatic ductal adenocarcinoma, SMARCD3 is considered an epigenetic regulator and a potential therapeutic target [12]. It is considered a possible risk factor for multiple myeloma [13].

We conducted this research to systematically assess the function of SMARCD3 across cancers and provide specific directions for subsequent research on SMARCD3.

## MATERIALS AND METHODS

### Pancancer data download

The gene expression, survival and clinicopathological parameter data of 33 cancer patients were extracted from the UCSC Xena online database (https://xenabrowser.net/datapages/) [14]. Our study method is presented in Supplementary Figure 1.

### ssGSEA gene activity analysis

Utilizing the single-sample gene set enrichment method (ssGSEA) [15], the activity scores of SMARCD3 in every cancer were determined according to the expression level of SMARCD3, and the values were compared between tumor and normal samples.

### Pancancer prognostic analysis

Using univariate Cox analysis, the associations between the expression level of SMARCD3 and clinicopathological characteristics and survival (including overall survival (OS), disease-specific survival (DSS), disease-free survival (DFS) and progression-free survival (PFS)) in 33 cancer types were examined. Survival rates were compared between groups with high and low SMARCD3 expression using Kaplan-Meier curves.

### Tumor microenvironment

After the expression data of 33 cancers were obtained, the “ESTIMATE” program was used to analyze the expression data; calculated the immune cell content (ImmuneScore), stromal cell content (StromalScore) and comprehensive content (ESTIMATEScore) [16]; and determine the association between the content of these cells and the SMARCD3 expression level. Then, the “CIBERSORT” package was used to analyze the immunological cells infiltrating 33 cancers, and the correlation between immune cells and SMARCD3 was assessed in samples grouped by the expression level of SMARCD3. For the correlation analysis, the screening criteria were R > 0.3 and *P* < 0.001.

### Immune gene correlation

TISIDB online tools were used to identify immunostimulatory genes, immunosuppressive genes and major histocompatibility complex (MHC) molecules significantly associated with SMARCD3 (http://cis.hku.hk/TISIDB/) [17].

### GSEA

Based on the expression of SMARCD3 (low vs. high) in the UCSC database, the samples were split into groups. The LogFC value of each sample was then computed based on the mean expression value, and gene set enrichment analysis (GSEA) was carried out. The Kyoto Encyclopedia of Genes and Genomes (KEGG) and Gene Ontology (GO) datasets were acquired from the GESA database (https://www.gsea-msigdb.org/gsea/index.jsp).

### Tumor mutation burden and microsatellite instability

TMB indicates the number of mutations in tumor samples, and the level of TMB affects the impact of immunotherapy and the immune response of malignancies [18]. In MSI, the DNA sequence changes in tumor cells due to the deletion or insertion of microsatellite DNA repeats [19]. MSI is associated with a response to tumor immunotherapy. The TCGA database provides TMB and MSI data.

### Immunotherapy analysis

GSE78220, GSE67501, and GSE126044 are public datasets for melanoma, renal cell carcinoma, and lung adenocarcinoma, respectively, while IMvigor210 is an immunotherapy dataset for bladder cancer. We used these four datasets to analyze the immunotherapy effect of SMARCD3 and analyzed the potential of SMARCD3 as an immunotherapy target [20–23]. The Gene Expression Omnibus (GEO) website was utilized to obtain the GSE78220, GSE67501, and GSE126044 datasets (https://www.ncbi.nlm.nih.gov/geo/). The IMvigor210 set was obtained by utilizing the IMvigor210 Core Biologies program.

### SMARCD3 immunohistochemical staining

The expression map of SMARCD3 in KIRC, LUAD, and STAD tumors as well as normal tissues can be found on the Human Protein Atlas website (http://www.Proteinatlas.org/) [24].

### Cell line culture

A human KIRC cell line (Caki-1), a normal kidney cell line (AD293), a human LUAD cell line (SPC-A-1), a normal lung cell line (BEAS-2B), a human STAD cell line (AGS) and a normal gastric cell line (GES-1) were acquired from Fuheng Biotechnology (Shanghai, China). The cells were cultivated in a media consisting of 1% penicillin/streptomycin (HyClone) supplemented with 10% fetal bovine serum (FBS; Sage Creation Science Co., Ltd., Beijing, China) in Dulbecco’s modified Eagle’s medium (DMEM; HyClone, Logan, UT, USA). The culture conditions included 5% CO_2_ and a temperature of 37°C.

### Real-time quantitative PCR

Tissue samples were collected from lung cancer patients who had undergone surgical resection at the Department of Thoracic Surgery, the Second Affiliated Hospital of Nanchang University. Six pairs of LUAD specimens and surrounding normal tissues were collected in total. After the samples were collected, they were rapidly frozen in liquid nitrogen and then kept in a refrigerator at −4°C.

Total RNA was extracted from cells using the TRIzol™ Plus RNA Purification Kit (Invitrogen, Thermo Fisher Scientific, Inc., USA) in accordance with the manufacturer’s instructions. The RNA was subsequently reverse transcribed into cDNA in accordance with the instructions provided by Takala, Japan’s PrimeScript RT Master Mix. The purity and concentration of the cDNA were evaluated. Next, we used the SYBR Premix Ex Taq II (Takara, Japan) kit to perform RT-qPCR. The protocol used for the polymerase chain reaction (PCR) included a 30 s denaturation stage at 95°C, 40 cycles of decomposition for 5 s at 95°C, 30 seconds of annealing at 60°C, 45 s of extension at 72°C, and a final 10 min extension step at 72°C. The internal reference gene used in the investigation was β-actin, and the data obtained were evaluated according to the guidelines provided in the literature [25]. The sequences of primers used for RT-qPCR was as follows: β-actin, 5′-CATCCGCAAAGACCTGTACG-3′, 5′-CCTGCTTGCTGATCCACATC-3′; SMARCD3, 5′-GGACGAAGTTGCCGGAGG-3′, 5′-TGGGGCATCCGGGCT-3′ (Supplementary Table 1). The primers used were obtained from Sangong Bioengineering Co., Ltd., (Shanghai, China).

## RESULTS

### Analysis of SMARCD3 expression across cancers

**Figure 1:**
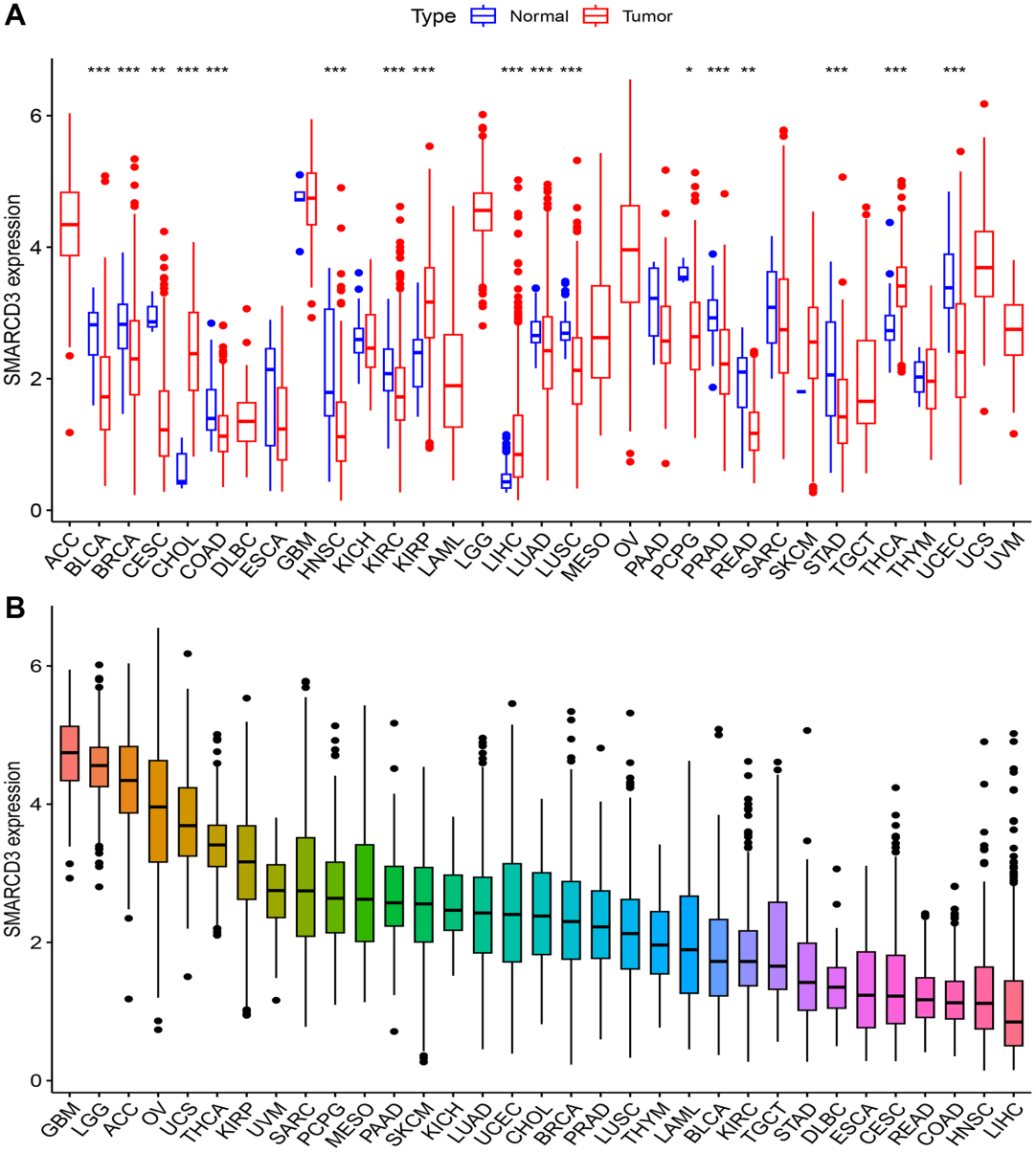
**Expression of SMARCD3 in pan-cancer.** (**A**) Expression difference of SMARCD3 between tumor samples and normal tissue; (**B**) Ranking of SMARCD3 expression in pan-cancer.

We evaluated the expression of SMARCD3 in 33 cancers. The results demonstrated that SMARCD3 expression differed between tumor and normal tissues for 17 distinct forms of cancer (*P* < 0.05), including bladder urothelial carcinoma (BLCA), breast invasive carcinoma (BRCA), cholangiocarcinoma (CHOL), cervical and endocervical cancer (CESC), colon adenocarcinoma (COAD), head and neck squamous cell carcinoma (HNSC), kidney papillary cell carcinoma (KIRP), kidney clear cell carcinoma (KIRC), liver hepatocellular carcinoma (LIHC), lung squamous cell carcinoma (LUSC), lung adenocarcinoma (LUAD), prostate adenocarcinoma (PRAD), pheochromocytoma and paraganglioma (PCPG), rectum adenocarcinoma (READ), stomach adenocarcinoma (STAD) and thyroid carcinoma (THCA) (Figure 1A). In 4 of these cancers (CHOL, KIRP, LIHC and THCA), SMARCD3 expression was lower in normal tissues than in tumor tissues. In the other 13 cancers, SMARCD3 was expressed at lower levels in tumor tissues. Furthermore, SMARCD3 was most highly expressed in brain lower grade glioma (LGG) and glioblastoma multiforme (GBM), although its expression in both healthy and tumorous tissues was similar. In the context of THCA, the expression level of SMARCD3 exhibited a substantial difference between cancerous tissues and healthy tissues (Figure 1B). Moreover, a research project was carried out to investigate the associations between clinicopathological characteristics and the expression of SMARCD3. However, clear associations were observed in a few cancer types, and the association patterns varied across cancers (Supplementary Figure 2). Table 1 is a summary of all the studies’ findings.

**Table 1:** Pan-cancer analysis results.

**Cancer**	**Difference analysis**	**Gene activity**	**OS**	**DFS**	**DSS**	**PFS**	**TMB**	**MSI**
ACC	NA	NA	NA	NA	NA	NA	NA	H
BLCA	L	L	NA	NA	NA	NA	L	NA
BRCA	L	L	NA	NA	NA	NA	L	H
CESC	L	L	NA	NA	NA	NA	NA	NA
CHOL	H	H	NA	NA	NA	NA	NA	NA
COAD	L	L	R	NA	R	R	NA	NA
DLBC	NA	NA	NA	NA	NA	NA	NA	NA
ESCA	NA	L	NA	NA	NA	NA	L	NA
GBM	NA	L	NA	NA	NA	NA	NA	NA
HNSC	L	L	R	R	NA	R	NA	NA
KICH	NA	L	NA	NA	NA	NA	NA	NA
KIRC	L	L	R	NA	R	NA	L	L
KIRP	H	NA	NA	NA	NA	NA	NA	NA
LAML	NA	NA	NA	NA	NA	NA	L	NA
LGG	NA	NA	NA	R	NA	NA	H	H
LIHC	H	NA	NA	NA	NA	NA	L	NA
LUAD	L	L	P	NA	P	NA	L	NA
LUSC	L	L	NA	NA	NA	NA	NA	NA
MESO	NA	NA	NA	NA	NA	R	NA	NA
OV	NA	NA	NA	NA	NA	NA	NA	NA
PAAD	NA	NA	NA	P	NA	P	L	L
PCPG	L	H	NA	NA	NA	NA	NA	NA
PRAD	L	L	NA	NA	NA	NA	L	NA
READ	L	L	NA	NA	NA	NA	NA	NA
SARC	NA	NA	NA	NA	NA	NA	L	NA
SKCM	NA	NA	NA	NA	NA	NA	NA	H
STAD	L	L	NA	R	R	R	L	L
TGCT	NA	NA	NA	NA	NA	NA	NA	NA
THCA	H	H	P	P	NA	NA	NA	H
THYM	NA	NA	NA	NA	NA	NA	NA	NA
UCEC	L	L	R	NA	R	R	L	NA
UCS	NA	NA	NA	NA	NA	NA	NA	NA
UVM	NA	NA	R	NA	R	R	NA	NA

Abbreviations: ACC: adrenocortical cancer; BLCA: bladder urothelial carcinoma; BRCA: breast invasive carcinoma; CESC: cervical and endocervical cancer; CHOL: cholangiocarcinoma; COAD: colon adenocarcinoma; DFS: disease-free survival; DSS: disease-specific survival; DLBC: diffuse large B-cell lymphoma; ESCA: esophageal carcinoma; GBM glioblastoma multiforme; GO: Gene Ontology; GSEA: Gene Set Enrichment; H: Highly expressed in tumor tissues; HNSC: head and neck squamous cell carcinoma; KEGG: Kyoto Encyclopedia of Genes and Genomes; KICH kidney chromophobe; KIRC: kidney clear cell carcinoma; KIRP: kidney papillary cell carcinoma; L: Low expression in tumor tissue; LAML: acute myeloid leukemia; LGG: brain lower grade glioma; LIHC: liver hepatocellular carcinoma; LUAD: lung adenocarcinoma; LUSC: lung squamous cell carcinoma; MESO: mesothelioma; MHC: major histocompatibility complex; MSI: microsatellite instability; NA: Meaningless; OS: overall survival; OV: ovarian serous cystadenocarcinoma; PAAD: pancreatic adenocarcinoma; PCPG: pheochromocytoma and paraganglioma; PFS: progression-free survival; PRAD: prostate adenocarcinoma; READ: rectum adenocarcinoma; SARC: sarcoma; SKCM: skin cutaneous melanoma; STAD: stomach adenocarcinoma; TGCT: testicular germ cell tumor; THCA thyroid carcinoma; THYM: thymoma; TMB: tumor mutational burden; UCEC: uterine corpus endometrioid carcinoma; UCS uterine carcinosarcoma; UVM: uveal melanoma.

### Gene activity analysis

We analyzed the gene activity of SMARCD3 in 33 cancers. There was a notable disparity in gene expression patterns between healthy and malignant tissues across a total of 18 distinct types of cancer, including BRCA, BLCA, CHOL, CESC, COAD, esophageal carcinoma (ESCA), GBM, HNSC, KIRC, kidney chromophobe (KICH), LUSC, LUAD, PRAD, PCPG, READ, STAD, THCA, and uterine corpus endometrioid carcinoma (UCEC). In CHOL, PCPG, and THCA, gene activity within tumor tissue was greater, while in the remaining 15 cancers, gene activity was higher in normal tissue (Supplementary Figure 3A). We also noted that the activity of the SMARCD3 gene in LGG, PCPG, and GBM was greater than that in other cancers (Supplementary Figure 3B). Considering these results and those of the difference analysis, we believe that the cancers that need to be focused on include BRCA, BLCA, CHOL, CESC, COAD, KIRC, HNSC, LUSC, LUAD, PRAD PCPG, and READ.

### Impact of SMARCD3 on prognosis

**Figure 2:**
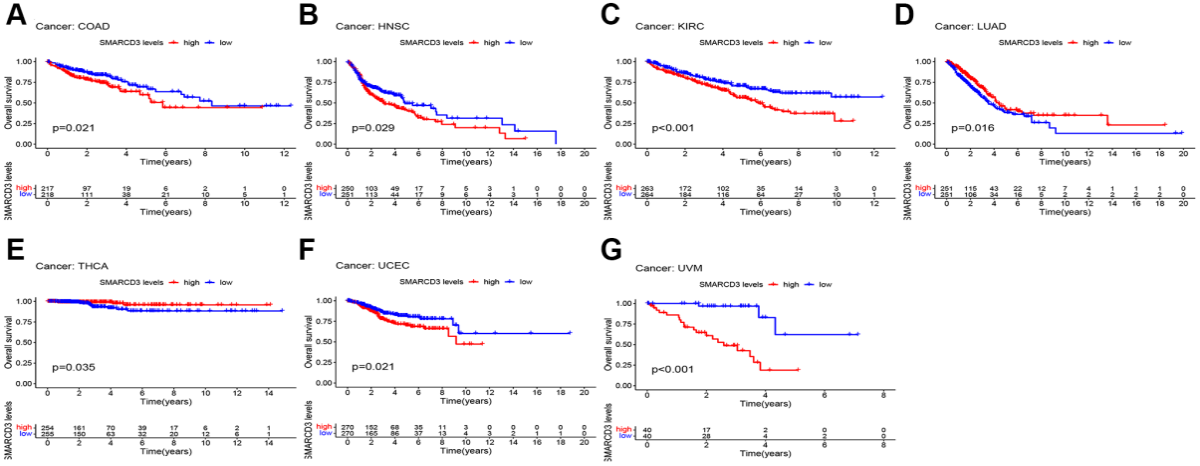
**KM survival curves based on overall survival in 7 cancers.** (**A**) COAD; (**B**) HNSC; (**C**) KIRC; (**D**) LUAD; (**E**) THCA; (**F**) UCFC; (**G**) UVM.

Subsequently, an evaluation was conducted to ascertain the prognostic relevance of SMARCD3 for various kinds of cancer. Supplementary Figure 4 shows the statistical significance of SMARCD3 expression in terms of OS, DFS, DSS, and PFS of several cohorts in cancer patients, as determined via univariate Cox analysis. These results and the KM survival curves indicated the following associations: In terms of OS, SMARCD3 was a risk factor in COAD, HNSC, KIRC, UCEC, and uveal melanoma (UVM), and a protective factor in LUAD and THCA (Figure 2). In terms of DSS, SMARCD3 was a risk factor for COAD, KIRC, STAD, UCEC and UVM and a protective factor for LUAD (Supplementary Figure 5). In terms of DFS, SMARCD3 was a risk factor in HNSC, LGG, and STAD and a protective factor for PAAD and THCA (Supplementary Figure 6). Finally, in terms of PFS, SMARCD3 was a risk factor for COAD, HNSC, mesothelioma (MESO), STAD, UCEC, and UVM and a protective factor against PAAD (Supplementary Figure 7). In summary, among the cancers whose prognosis is affected by SMARCD3, those worthies of focus include COAD, HNSC, KIRC, LUAD, STAD, and UCEC. The statistics for this particular prognostic analysis are displayed in Supplementary Tables 2–5.

### Tumor microenvironment analysis

**Figure 3:**
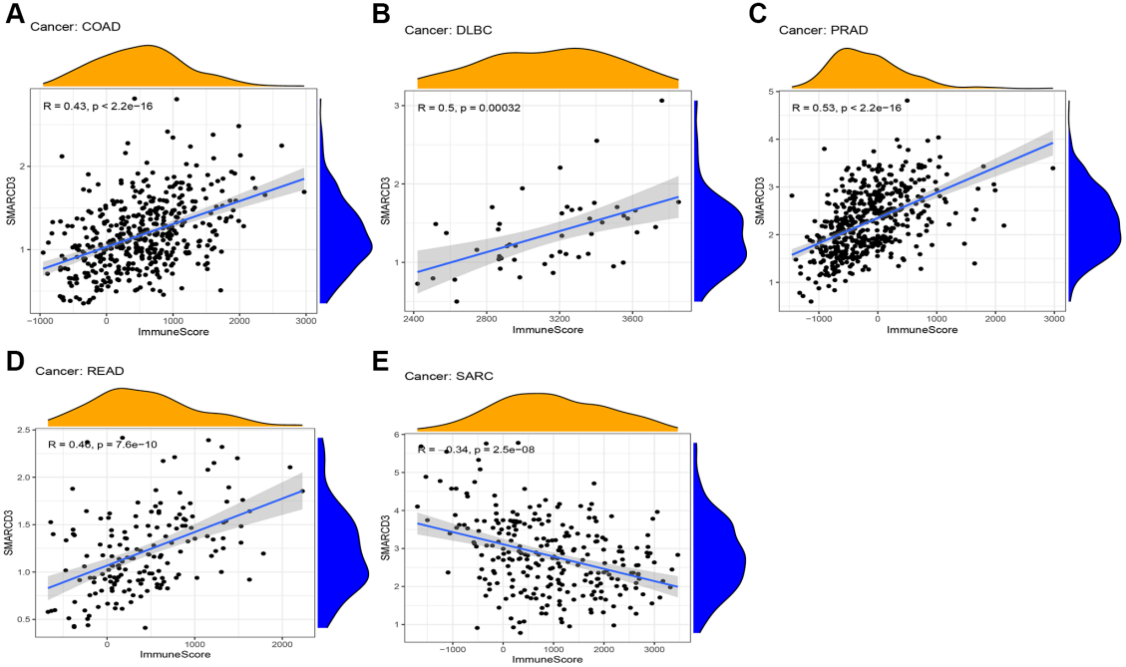
**Correlation between ImmuneScore and SMARCD3 expression in 5 cancers.** (**A**) COAD; (**B**) DLBC; (**C**) PRAD; (**D**) READ; (**E**) SRAC.

We performed a correlation analysis on the tumor microenvironments of 33 cancer types. Favorable interactions between immune cell infiltration and SMARCD3 expression were detected in COAD, DLBC, PRAD, and READ. However, a negative correlation was observed for the SARC (Figure 3). The infiltration of stromal cells was positively correlated with COAD, ESCA, LIHC, HNSC, READ, PRAD, and STAD, testicular germ cell tumor (TGCT), and thymoma (THYM), and negatively correlated with GBM and SARC (Supplementary Figure 8). The ESTIMATEScore was positively correlated with SMARCD3 expression in COAD, PRAD, and READ, indicating that immune cells and stromal cells are highly infiltrated in these three cancers (Supplementary Figure 9A–9C). The exact values used in this study are listed in Table 2. Subsequently, the infiltration of particular immune cell types was examined using the CIBERSORT algorithm. We found that SMARCD3 expression was positively correlated with the levels of monocytes, resting dendritic cells, M2 macrophages, and resting mast cells and negatively correlated with those of naive B cells, eosinophils, M1 macrophages, neutrophils, focal helper T cells, plasma cells, and activated memory CD4 T cells in certain cancers. All the findings of the CIBERSORT algorithm are shown in Supplementary Figure 10.

**Table 2:** Correlation of SMARCD3 expression with stromal cells and immune cells.

**Cancer**	**P-StromalCell**	**P-ImmuneCell**
ACC	0.03	0.03
BLCA	0.01	0.68
BRCA	0.01	0.03
CESC	0.00	0.34
CHOL	0.76	0.85
COAD	0.00	0.00
DLBC	0.06	0.00
ESCA	0.00	0.28
GBM	0.00	0.00
HNSC	0.00	0.13
KICH	0.30	0.50
KIRC	0.00	0.91
KIRP	0.05	0.62
LAML	0.00	0.00
LGG	0.00	0.00
LIHC	0.00	0.00
LUAD	0.02	0.02
LUSC	0.02	0.06
MESO	0.12	0.02
OV	0.00	0.01
PAAD	0.02	0.01
PCPG	0.70	0.65
PRAD	0.00	0.00
READ	0.00	0.00
SARC	0.00	0.00
SKCM	0.24	0.01
STAD	0.00	0.00
TGCT	0.00	0.01
THCA	0.00	0.11
THYM	0.00	0.26
UCEC	0.34	0.19
UCS	0.79	0.01
UVM	0.01	0.01

Abbreviations: ACC: adrenocortical cancer; BLCA: bladder urothelial carcinoma; BRCA: breast invasive carcinoma; CESC: cervical and endocervical cancer; CHOL: cholangiocarcinoma; COAD: colon adenocarcinoma; DLBC: diffuse large B-cell lymphoma; ESCA: esophageal carcinoma; GBM glioblastoma multiforme; GO: Gene Ontology; GSEA: Gene Set Enrichment; HNSC: head and neck squamous cell carcinoma; KEGG: Kyoto Encyclopedia of Genes and Genomes; KICH kidney chromophobe; KIRC: kidney clear cell carcinoma; KIRP: kidney papillary cell carcinoma; LAML: acute myeloid leukemia; LGG: brain lower grade glioma; LIHC: liver hepatocellular carcinoma; LUAD: lung adenocarcinoma; LUSC: lung squamous cell carcinoma; MESO: mesothelioma; MHC: major histocompatibility complex; OV: ovarian serous cystadenocarcinoma; PAAD: pancreatic adenocarcinoma; PCPG: pheochromocytoma and paraganglioma; P-ImmuneCell: *P*-value of Immune Cell; P-StromalCell: *P*-value of Stromal Cell; PRAD: prostate adenocarcinoma; READ: rectum adenocarcinoma; SARC: sarcoma; SKCM: skin cutaneous melanoma; STAD: stomach adenocarcinoma; TGCT: testicular germ cell tumor; THCA thyroid carcinoma; THYM: thymoma; UCEC: uterine corpus endometrioid carcinoma; UCS uterine carcinosarcoma; UVM: uveal melanoma.

### Immune gene correlation analysis

**Figure 4:**
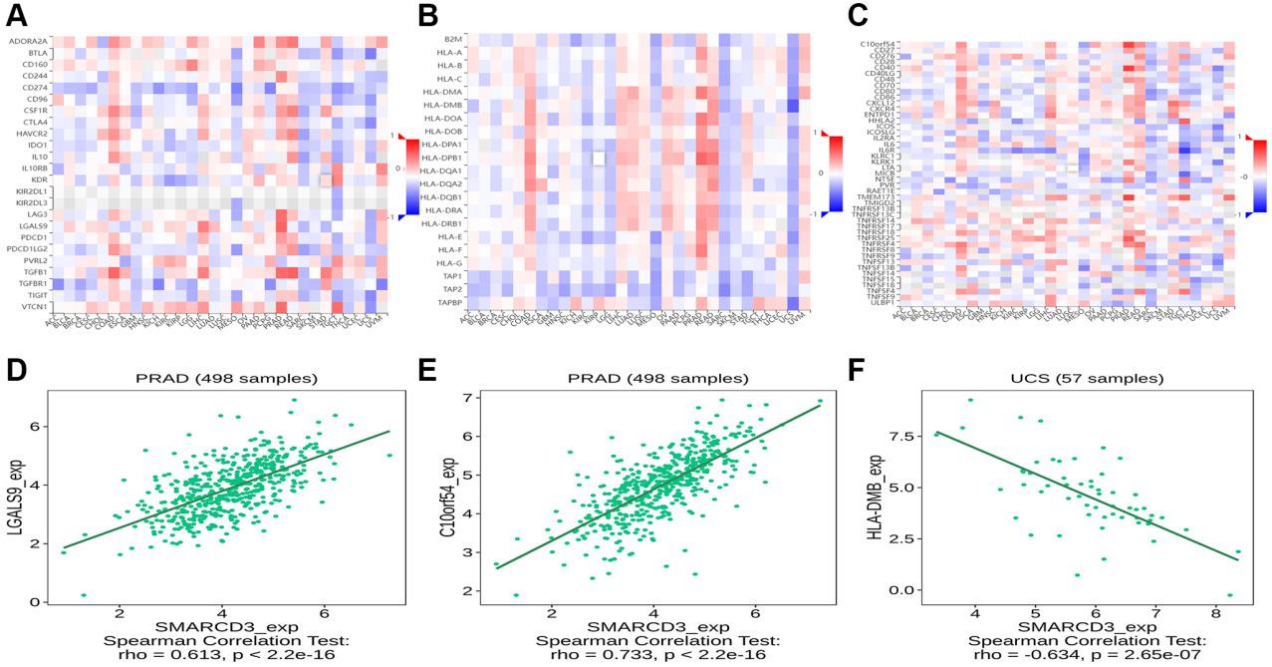
**Correlation analysis between SMARCD3 and immune-related genes.** (**A**) Correlation heat map of SMARCD3 and Immunoinhibitor; (**B**) Correlation heat map of SMARCD3 and Immunostimulator; (**C**) Correlation heat map of SMARCD3 and MHC; (**D**) Correlation map of SMARCD3 and LGALS9 in PRAD; (**E**) Correlation map of SMARCD3 and C10or54 in PRAD; (**F**) Correlation map of SMARCD3 and HLA-DMB in UCS.

We used TISIDB online tools to determine the associations between SMARCD3 and immune-related genes. The results revealed that SMARCD3 was significantly correlated with multiple immune-related genes. In the analysis of immunoinhibitors, SMARCD3 showed a positive correlation with LGALS9 and C10orf54 in PRAD, was positively correlated with TGFβ1 in READ, and demonstrated a negative correlation with CD274 and BTLA in MESO and UCS, respectively. According to the immunostimulatory analysis, SMARCD3 target genes were negatively correlated with LTA and IL6R in TGCT and positively correlated with CD40 in PRAD. Finally, in the MHC molecule analysis, target genes showed a positive correlation with HLA-DPB1 in both the PRAD and READ cohorts but demonstrated a negative correlation with HLA-DMB and TAPBR in the UCS cohort (Figure 4). The remaining relevant genes are shown in Supplementary Figure 11.

### GSEA

We performed GSEA of SMARCD3-related genes. We found that SMARCD3 is involved in many biological processes. First, SMARCD3 is undoubtedly involved in chromatin remodeling, DNA transcription and activation. We also found that SMARCD3 is involved in the metabolism of various substances and the activities of various immune cells. Among them, SMARCD3 participates in or affects lipid metabolism in CHOL, CESC, DLBC, ESCA, KIRP, LGG, LAML, OV, PRAD, PCPG, STAD, SARC, THCA and UCS. It affects the activities of a series of immune cells (neutrophils, T cells, dendritic cells, natural killer cells, etc.) in 24 cancer types including ACC, BRCA, COAD, KIRP, GBM, KICH, LGG, LAML, LIHC, LUSC, LUAD, OV, MESO, PRAD, READ, PCPG, STAD, SKCM, TGCT, THYM, THCA, UCEC, UVM and UCS. This shows that SMARCD3 affects substance metabolism, especially lipid metabolism, and may thereby affect tumor progression. It also affects the tumor microenvironment and may influence tumor development, immune evasion, and the immune response. Finally, aging-related pathways were enriched in BLCA, COAD, KIRP, TGCT and UVM, proving that SMARCD3 is related to cellular aging (Supplementary Figures 12, 13). The top 10 enriched pathways from our pan cancer screen are displayed in Supplementary Tables 6, 7.

### Analysis of TMB and MSI

**Figure 5:**
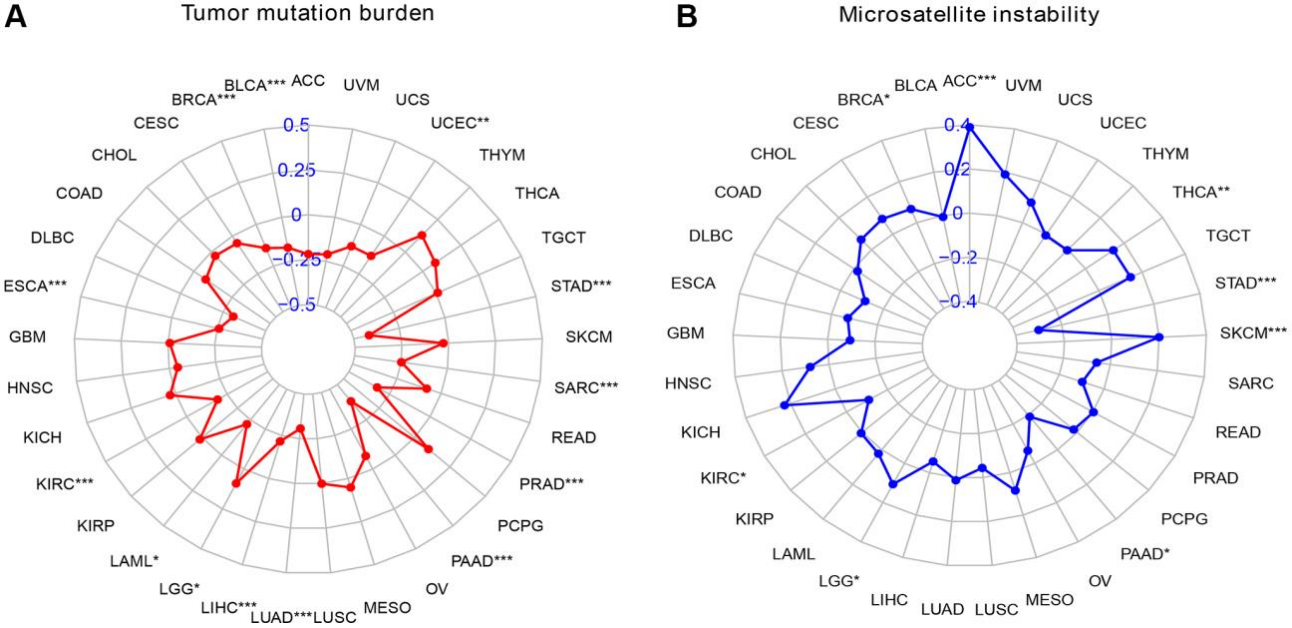
**Radar map of TMB versus MSI scores in pan-cancer.** (**A**) Radar chart of TMB score; (**B**) Radar chart of MSI score.

First, we analyzed the relationship between SMARCD3 and tumor mutational burden in 33 cancers. We observed that SMARCD3 expression was negatively correlated with the TMB in BRCA, BLCA, ESCA, KIRC, LAML, LUAD, LIHC, PRAD, PAAD, STAD, SARC, and UCEC, while it was positively correlated with that in LGG (Figure 5A). Next, microsatellite instability was assessed. SMARCD3 was negatively correlated with MSI in KIRC, PAAD, and STAD and positively correlated with MSI in the remaining BRCA, ACC, LGG, SKCM, and THCA (Figure 5B). Combining gene expression and gene activity analysis, we found that in most cancers with low SMARCD3 expression and activity, such as BLCA, LUAD, KIRC, PAAD and STAD. The TMB and MSI of cancer are negatively correlated with the expression of SMARCD3; that is, low expression and activity of SMARCD3 are associated with increased TMB and MSI. The effects of SMARCD3 on chromatin remodeling, DNA synthesis and DNA damage repair are likely to be among the mechanisms underlying this process, as this gene affects the course and prognosis of malignant tumors. The findings obtained from the aforementioned analysis are summarized in Supplementary Tables 8, 9. Considering these results and those of previous studies, SMARCD3 has achieved relatively ideal results in KIRC, LUAD, STAD and UCEC. We believe that SMARCD3 has more research value among these four cancers.

### Immunotherapy analysis

**Figure 6:**
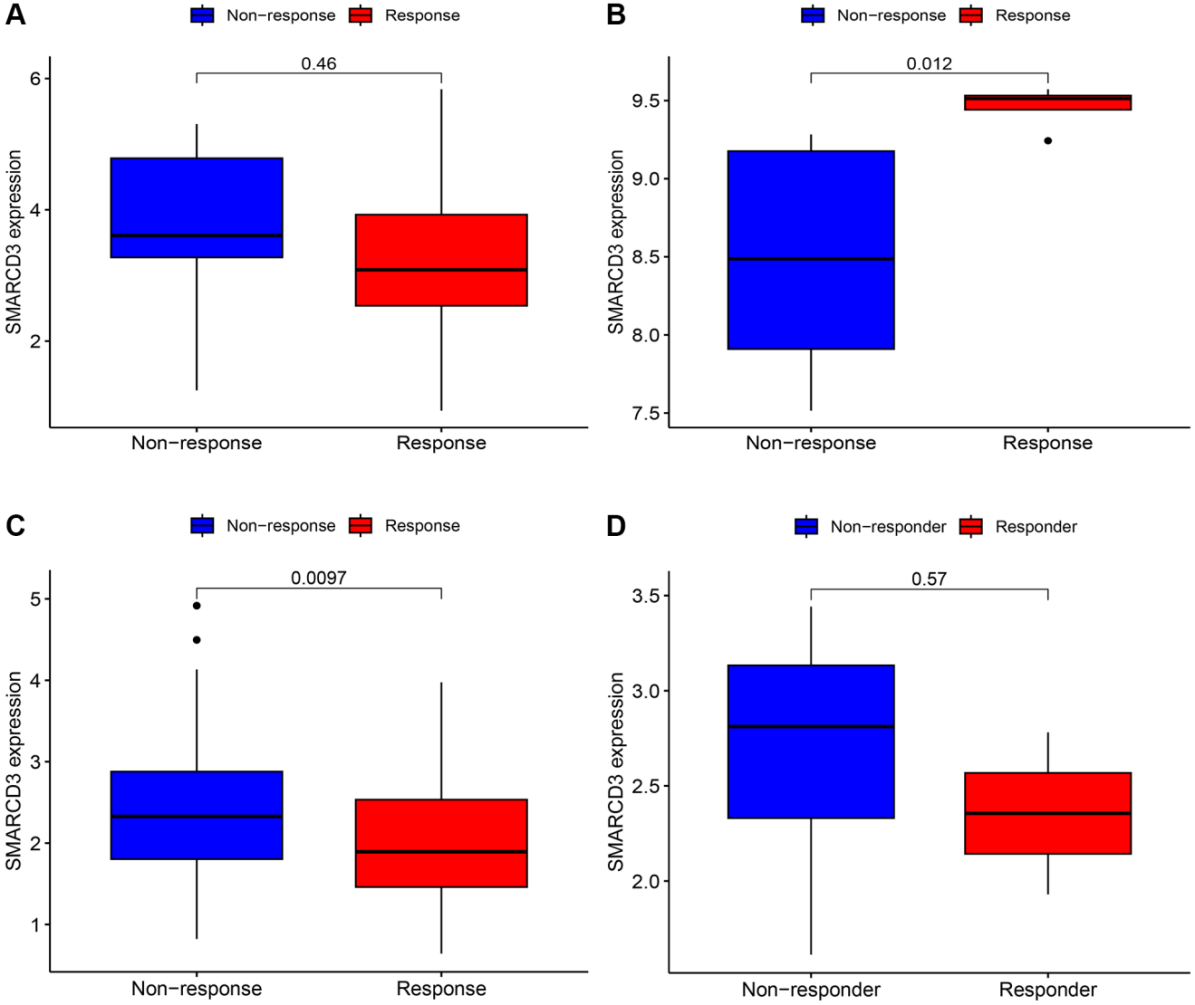
**Immunotherapy analysis of SMARCD3 in three cohorts.** (**A**) Immunotherapy analysis of SMARCD3 in the GSE78220 cohort; (**B**) Immunotherapy analysis of SMARCD3 in the GSE67501 cohort; (**C**) Immunotherapy analysis of SMARCD3 in the IMvigor210 cohort. (**D**) Immunotherapy analysis of SMARCD3 in the GSE126044 cohort.

We performed immunotherapy analysis of SMARCD3 using the GSE78220, GSE67501, IMvigor210 and GSE126044 cohorts. SMARCD3 was strongly expressed in the responder group of the GSE67501 cohort and in the nonresponder group of the IMvigor210 cohort; however, the data did not reveal a significant difference between the GSE78220 and GSE126044 cohorts. This finding suggested that SMARCD3 is a potential immunotherapy target with heterogeneous expression and implications across cancers (Figure 6A–6D).

### Immunohistochemistry and *in vitro* validation of SMARCD3

Immunohistochemical analysis based on the HPA database revealed that the expression of SMARCD3 in normal tissues was greater than that in tumor tissues in KIRC and STAD, as depicted in Supplementary Figure 14A. In this study, we used KIRC, LUAD, and STAD tumor and normal cell lines to assess and validate the expression levels and variations of SMARCD3. The findings of the study indicated that the levels of SMARCD3 expression in tumor cell lines from three different types of cancer were lower than those in normal cell lines. Moreover, there were statistically significant variations in the expression levels, as depicted in Supplementary Figure 14B. Next, we further verified the expression of SMARCD3 in human tissues. We obtained cancer tissues and adjacent normal tissues from six LUAD patients for experimental verification. As shown in Supplementary Figure 15, the expression of SMARCD3 differed between LUAD cancer tissues and normal tissues, with low expression in cancer tissues. The obtained outcome provides validation for our prior research and substantiates the degree of accuracy of our study.

### Availability of data and material

The data sets used and/or analyzed during the current study are available from the corresponding author on reasonable request.

## DISCUSSION

With the development of precision medicine, cancer treatment is becoming more individualized and precise [26]. In recent years, researchers have focused on exploring key genes related to cancer, identifying new approaches for cancer therapy, and identifying novel antitumor medications [27]. SMARCD3 is a very promising gene that has been demonstrated to be a key component in cancers such as breast cancer and medulloblastoma. However, to date, SMARCD3 has not been well studied, and its involvement in cancer has not been fully explained. Therefore, the purpose of this research was to reveal the function of SMARCD3 across cancers and to offer a novel avenue for investigating its mode of action in particular cancer types. In our study, we found heterogeneity in the expression and role of SMARCD3 in various cancers, but its effects on cancer are mostly unfavorable, and it is a risk factor for various cancers. GSEA analysis revealed the involvement of SMARCD3 in chromatin remodeling and transcriptional activation, as well as its role in the metabolism of diverse substances, particularly lipid metabolism, and the modulation of various immune cell activities. Immune gene correlation analysis proved that SMARCD3 is strongly connected to genes involved in immunity, such as CD40, HLA-DMB, and TAPBR. Immunotherapy analysis demonstrated that it affected the effectiveness of immunotherapy in renal cell carcinoma and bladder cancer cohorts.

First, we analyzed SMARCD3 expression and patient prognosis in 33 cancers. It was differentially expressed in 17 tumor tissues compared with normal tissues of different cancer types, and its expression was heterogeneous across cancers. However, in most cancers, SMARCD3 is expressed at low levels in tumor tissues. For example, cancers such as COAD, HNSC, and LUAD. A study on colorectal cancer supported our results [28]. However, in most cancers, SMARCD3 expression is lower in tumor tissues. We also found that the activity of target genes was low in most tumor tissues. There seems to be a special mechanism by which the expression and gene activity of SMARCD3 are inhibited in cancer. For example, SMARCD3 transcription is reduced in ER+ breast tumor cells due to methylation of the SMARCD3 promoter, but in ER+ breast cancer, SMARCD3 is still regarded as a tumor suppressor gene [29]. Next, we created KM curves to evaluate the predictive significance of SMARCD3 across cancers. We focused on HNSC, COAD, KIRC, LUAD, UCEC, and STAD. SMARCD3 affects the prognosis of these 5 cancers. Moreover, the prognosis was better for individuals with high SMARCD3 expression in LUAD than for those with low SMARCD3 expression. Compared to patients with high SMARCD3 expression, those with reduced SMARCD3 expression had a better prognosis for the remaining 4 cancers. Moreover, the results of the TMB and MSI analyses also attracted our attention. We found that SMARCD3 expression was negatively correlated with the TMB and MSI in most cancers, especially in KIRC, LUAD, STAD and UCEC. Low SMARCD3 expression was associated with high TMB and MSI. Considering the impact of SMARCD3 on chromatin remodeling and DNA transcription, we believe that the loss of SMARCD3 is likely to lead to abnormalities in DNA damage repair, which may be a potential mechanism by which SMARCD3 affects the aggressiveness and malignancy of tumors. Moreover, low SMRCD3 expression may also affect the sensitivity of tumors to immunotherapy. As a result, we performed an immunotherapy and discovered that in the renal cell carcinoma (GSE67501) and bladder cancer (IMvigor210) datasets, there were notable changes in the immune response effects among the SMARCD3 low and high-expression groups. This finding suggested that SMARCD3 influences the efficacy of cancer immunotherapy. Unfortunately, the lung adenocarcinoma dataset did not yield meaningful results due to the small sample size. RT-qPCR experiments on KIRC, LUAD and STAD cell lines and LUAD patient tissues verified the effectiveness of our approach to a certain extent. Overall, we contend that SMARCD3 influences the prognosis of UCEC, COAD, LUAD, KIRC, and STAD patients. Furthermore, this gene is likely crucial for KIRC, LUAD, and STAD.

Next, we evaluated the relationships of SMARCD3 with the ImmuneScore and StromalScore in different cancers. Notably, SMARCD3 was negatively correlated with the immune score in SARC patients and was positively correlated with the stromal score, immune score and ESTIMATEScore in COAD, PRAD, and READ patients. A study focused on the CD2 gene of breast cancer revealed that patients with high immune scores had improved OS, proving that the immune component of the immune microenvironment is a potential favorable factor in terms of the prognosis of BRCA patients [30]. The same results were obtained in a study of colorectal cancer [31]. The CIBERSORT algorithm derives links between target genes and immune cells in some specific cancers. The analysis of SMARCD3 and immune-related genes, revealed it is connected to a variety of immune-related genes. For example, in PRAD, SMARCD3 is positively correlated with CD40, and CD40 has been shown to be active in B cells and myeloid cells with high SMARCD3 expression [32]. In UCS patients, SMARCD3 was negatively correlated with HLA-DMB and TAPBR. HLA-DMB has been shown to affect antigen presentation and activation in CD4+ T cells [33].

GSEA revealed that SMARCD3 is involved in various metabolic processes and immune cell activities. First, in 14 tumors, we observed pathways closely related to lipid metabolism (e.g., GOBP_REGULATION OF TRIGLYCERIDE METABOLIC PROCESS; and GOBP_LONG CHAIN FATTY ACID METABOLIC PROCESS). Researchers have shown that in cancer stem cells, cholesterol and lipid metabolism are related to various cell signaling pathways and drug resistance [34, 35]. This may be a mechanism by which SMARCD3 affects cancer development. Second, we identified multiple pathways related to immune cell activity (e.g., GOBP_ALPHA BETA T CELL ACTIVATION; GOBP_POSITIVE REGULATION OF NATURAL KILLER CELL MEDIATED IMMUNITY) in 24 cancer types. Several studies have shown that immune cells inside the tumor microenvironment can either stimulate or suppress the growth of tumors [36, 37]. Our research revealed that immune cells mainly affected by SMARCD3 include natural killer cells, neutrophils, dendritic cells, and T cells. In a study on breast cancer, researchers found that the tumor-secreted protease cathepsin C (CTSC) promotes breast cancer lung metastasis by affecting the infiltration of neutrophils [38]. In a study of EMP3, researchers found that EMP3 inhibits T-cell infiltration in GBM and promotes tumor progression [39]. SMARCD3 may also promote or inhibit the development and occurrence of tumors by impacting immune cell activities in the tumor microenvironment. Finally, aging-related pathways were found to be enriched in 5 tumors. Given that SMARCD3 affects DNA damage repair, cellular aging may also be one of the mechanisms by which SMARCD3 affects tumor development [7].

Our study systematically evaluated the function of SMARCD3 across cancers. This study provides direction for further research on SMARCD3. We examined the expression features and prognostic significance of SMARCD3 across cancers and found that SMARCD3 may be an important gene affecting COAD, HNSC, KIRC, LUAD, STAD and UCEC. These results and those of the TMB, MSI and immunotherapy analyses indicated that SMARCD3 affects the prognosis and immunotherapy response of KIRC, STAD and LUAD patients, which has important research value. GSEA revealed that SMARCD3 may exert effects on lipid metabolism and immune cell activity as potential mechanism in cancer. However, our study has limitations. Our study relied on public databases, and the relevant results still require further experimental verification.

## CONCLUSION

Based on differential analysis and survival analysis, we found that SMARCD3 affects the prognosis of multiple cancers, especially COAD, HNSC, KIRC, LUAD, STAD, and UCEC. These results and those of the TMB, MSI and immunotherapy analyses indicate that SMARCD3 may have high research value in KIRC, LUAD and STAD. GSEA revealed that SMARCD3 affects lipid metabolism and the activity of immune cells in tumors. Our study revealed that SMARCD3 may be an important gene and a potential tumor target and provides a direction for further research. However, due to the limitations of this study, the specific mechanism and value of SMARCD3 still need to be verified through basic and clinical experiments.

## Supplementary Materials

Supplementary Figures

Supplementary Tables 1-5 and 8-9

Supplementary Table 6

Supplementary Table 7
