# Supplementary material for: Immunotherapy, prognostic, and tumor biomarker based on pancancer analysis, SMARCD3
Source: Aging (Albany NY). 2024 Jun 11;16(11):10074–107. doi: 10.18632/aging.205921 (PMC11210247; doi:10.18632/aging.205921)
Supplement: Supplementary Figures [file aging-16-205921-s001.pdf]

SUPPLEMENTARY FIGURES

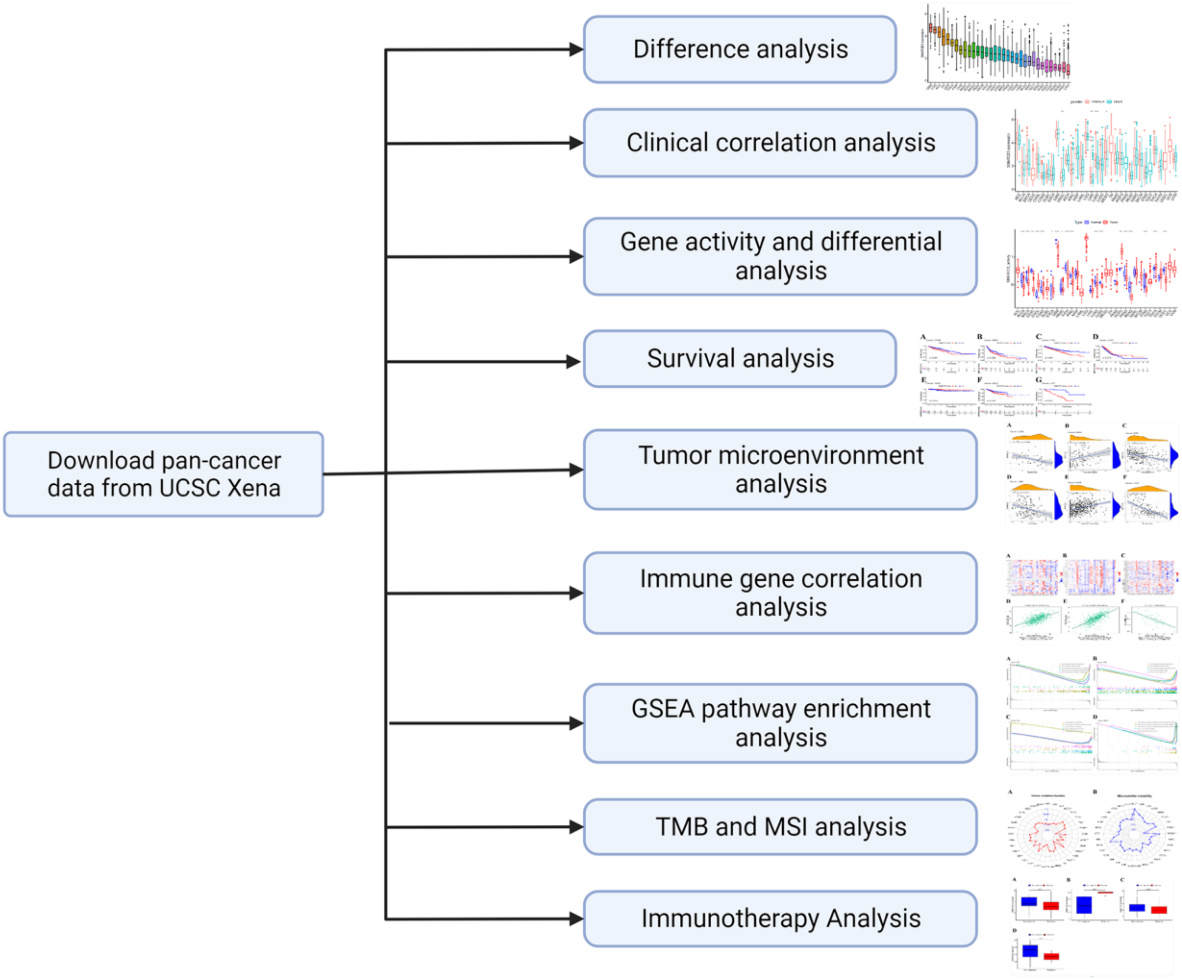

Supplementary Figure 1. Flow chart.

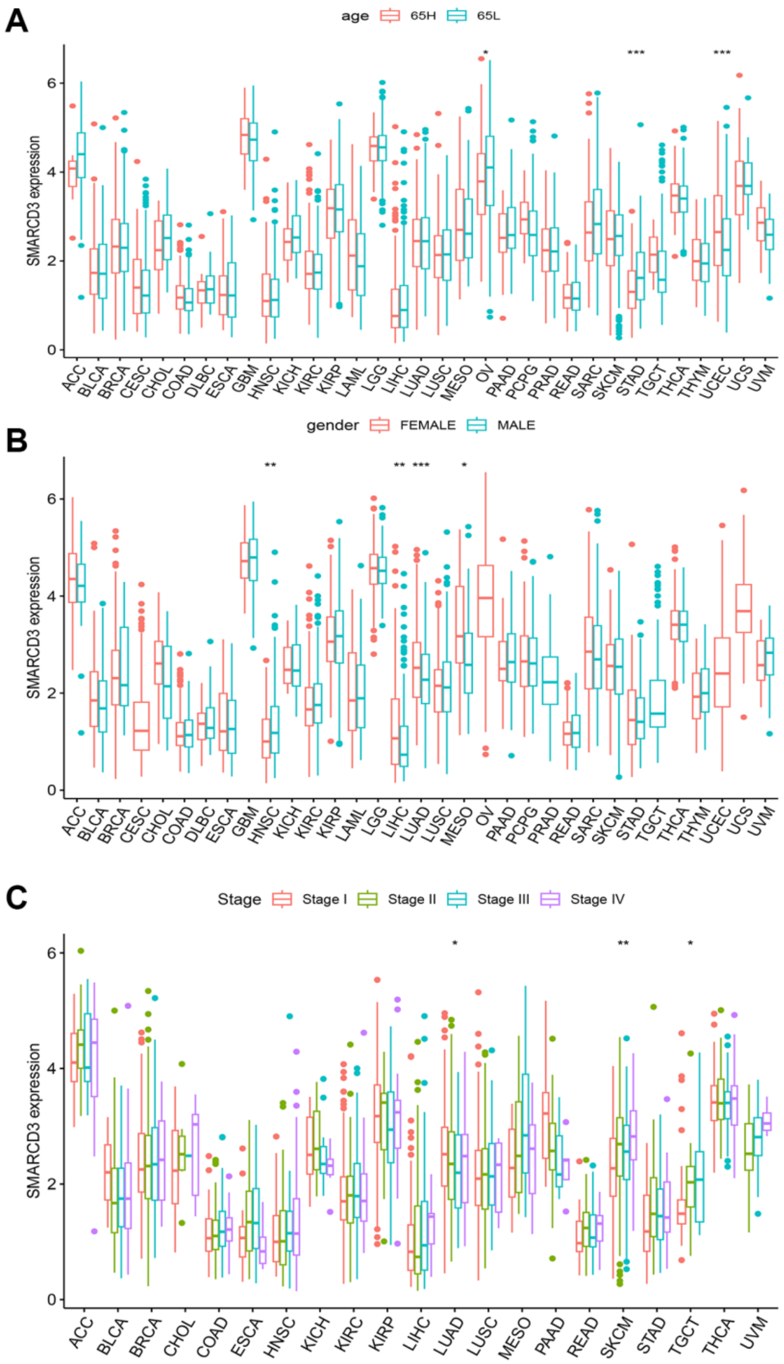

Supplementary Figure 2. Clinical correlation analysis of SMARCD3. (**A**) Relationship between SMARCD3 expression and age; (**B**) Relationship between SMARCD3 expression and gender; (**C**) Relationship between SMARCD3 expression and Stage.

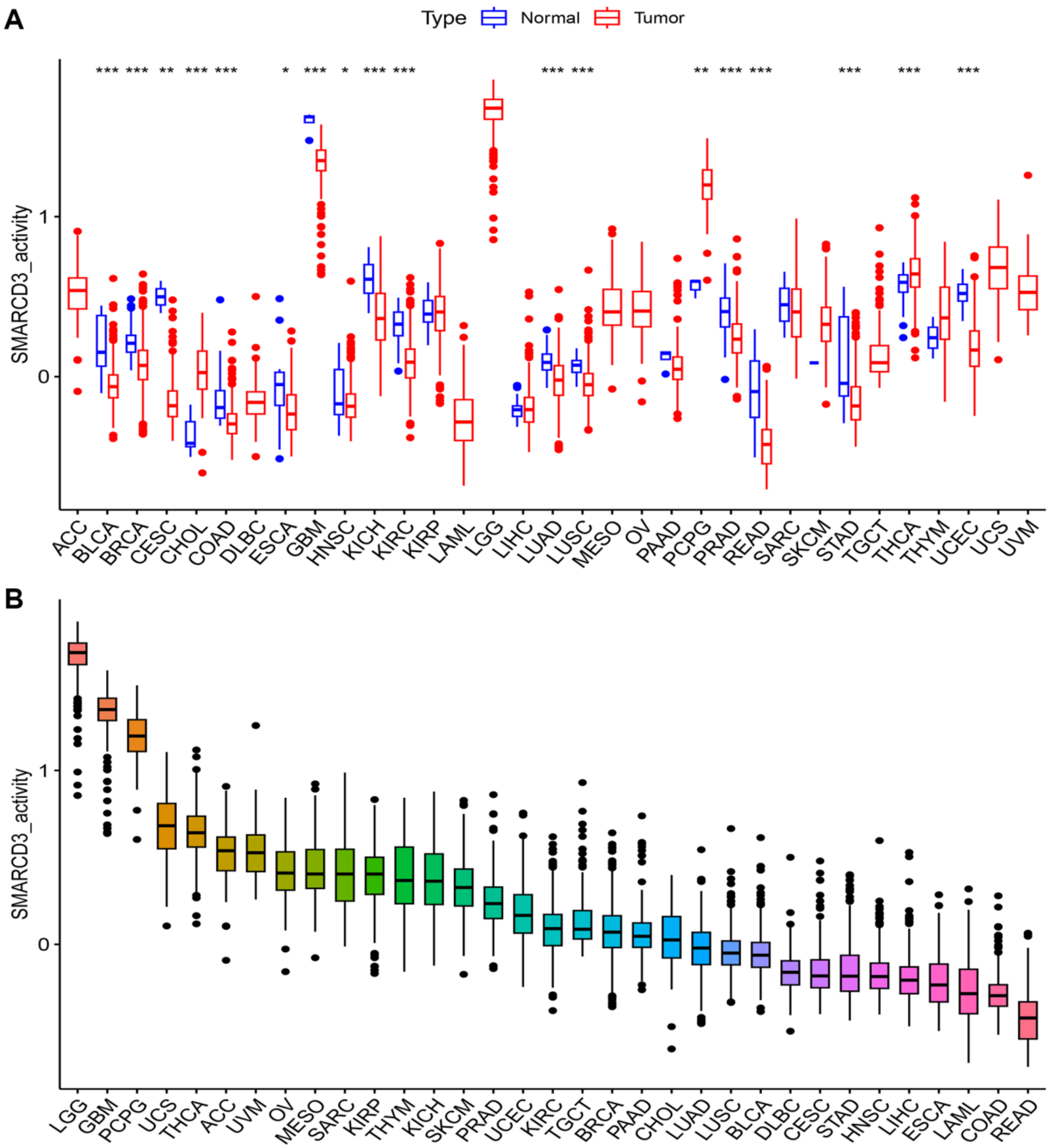

Supplementary Figure 3. Gene activity analysis of SMARCD3. (**A**) The difference of SMARCD3 gene activity between normal tissue and tumor tissue. (**B**) Ranking of SMARCD3 gene activity in pan-cancer.

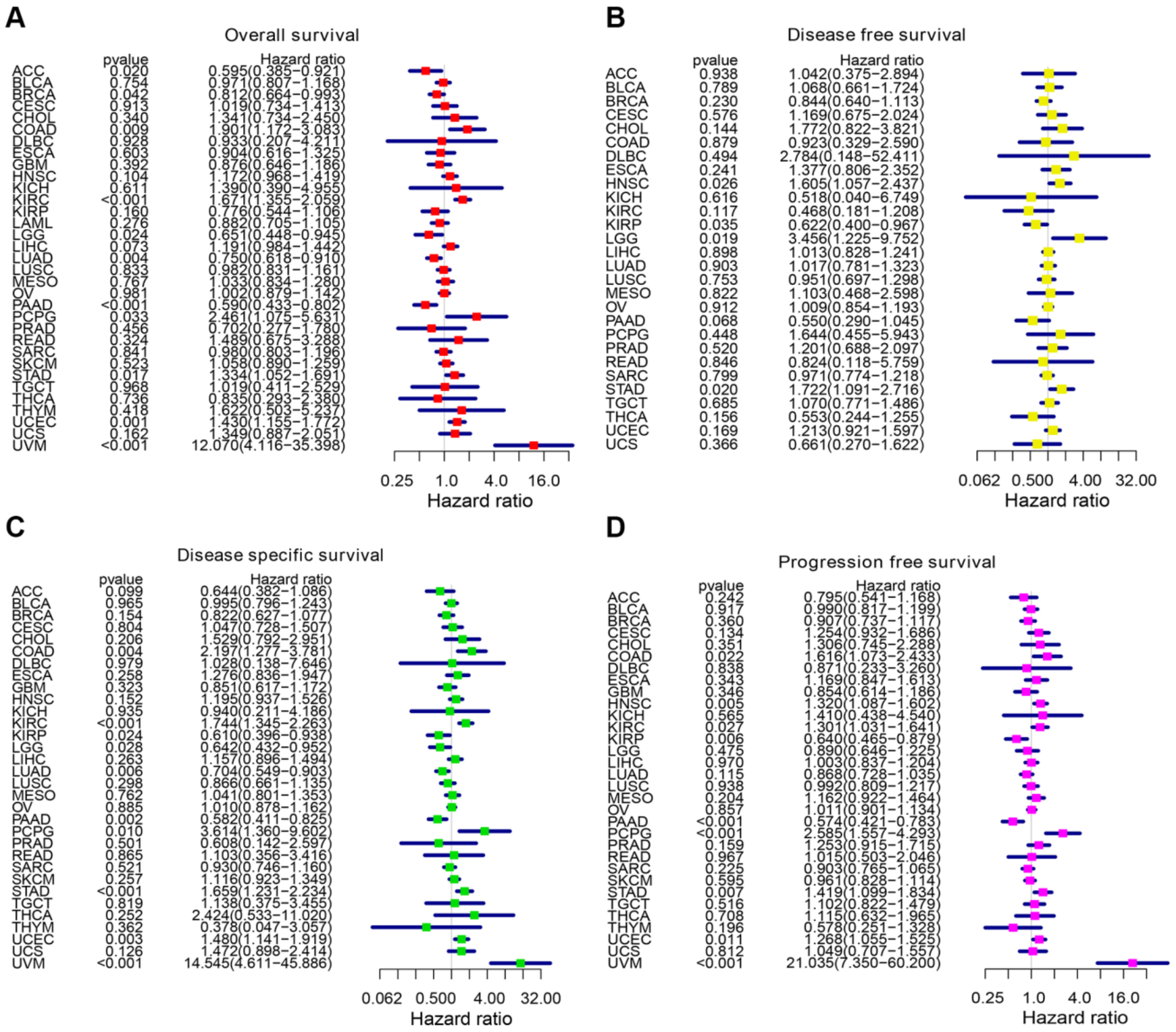

Supplementary Figure 4. Forest plot of correlation between SMARCD3 expression and survival time in pan-cancer. (**A**) Univariate COX analysis of SMARCD3 and OS; (**B**) Univariate COX analysis of SMARCD3 and DFS; (**C**) Univariate COX analysis of SMARCD3 and DSS; (**D**) Univariate COX analysis of SMARCD3 and PFS.

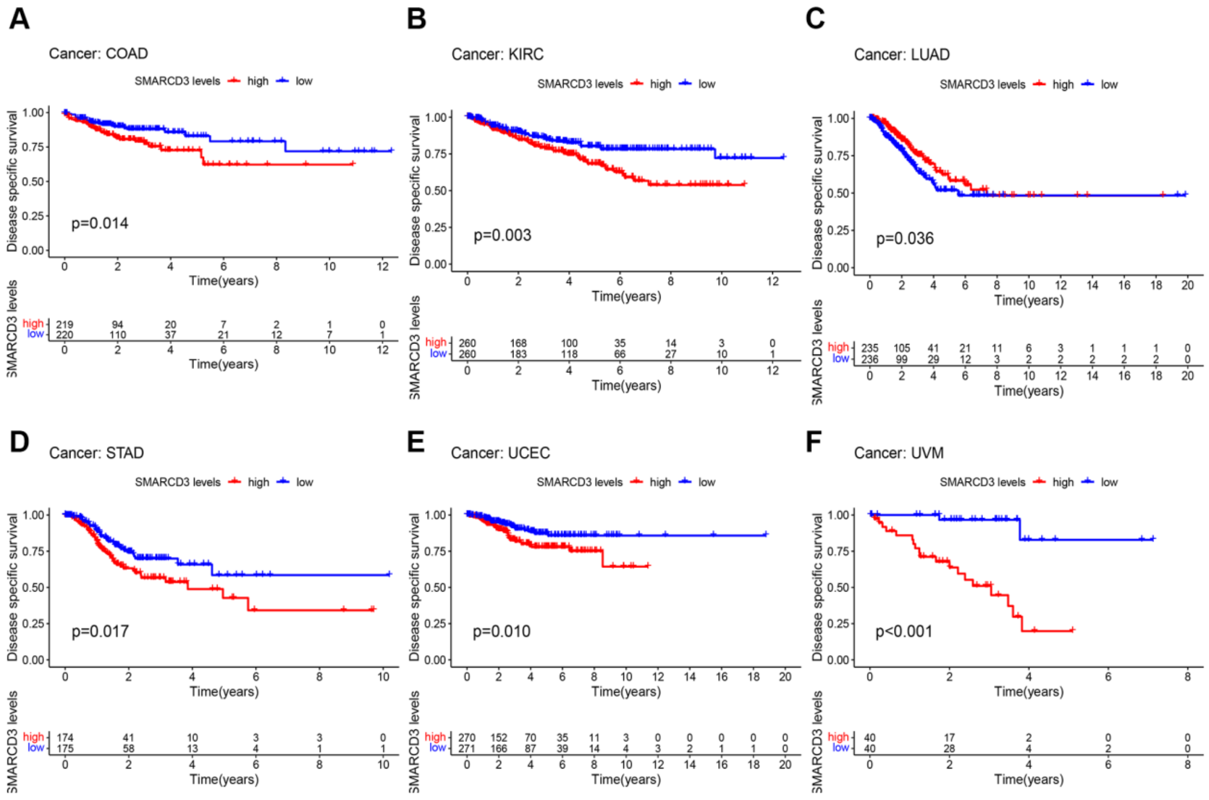

Supplementary Figure 5. KM survival curves based on disease-specific survival in 6 cancers. (**A**) COAD; (**B**) KIRC; (**C**) LUAD; (**D**) STAD; (**E**) UCEC; (**F**) UVM.

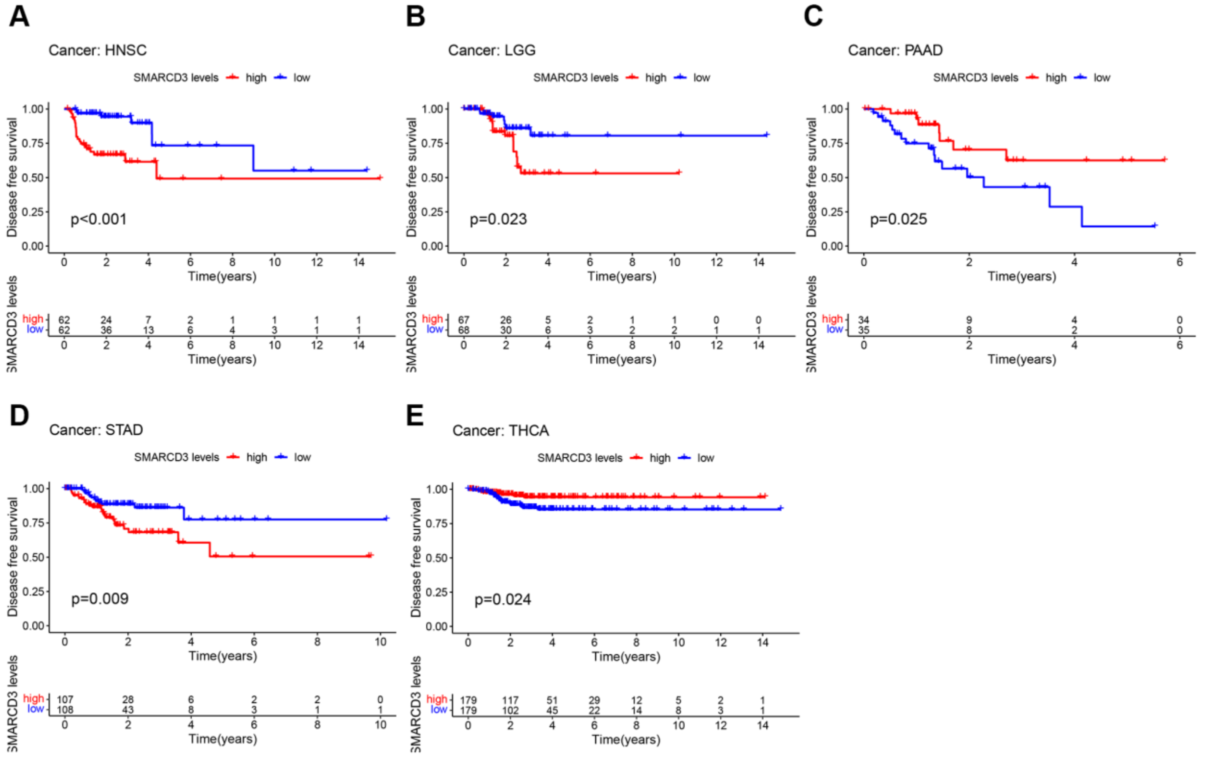

Supplementary Figure 6. KM survival curves based on disease-free survival in 5 cancers. (**A**) HNSC; (**B**) LGG; (**C**) PAAD; (**D**) STAD; (**E**) THCA.

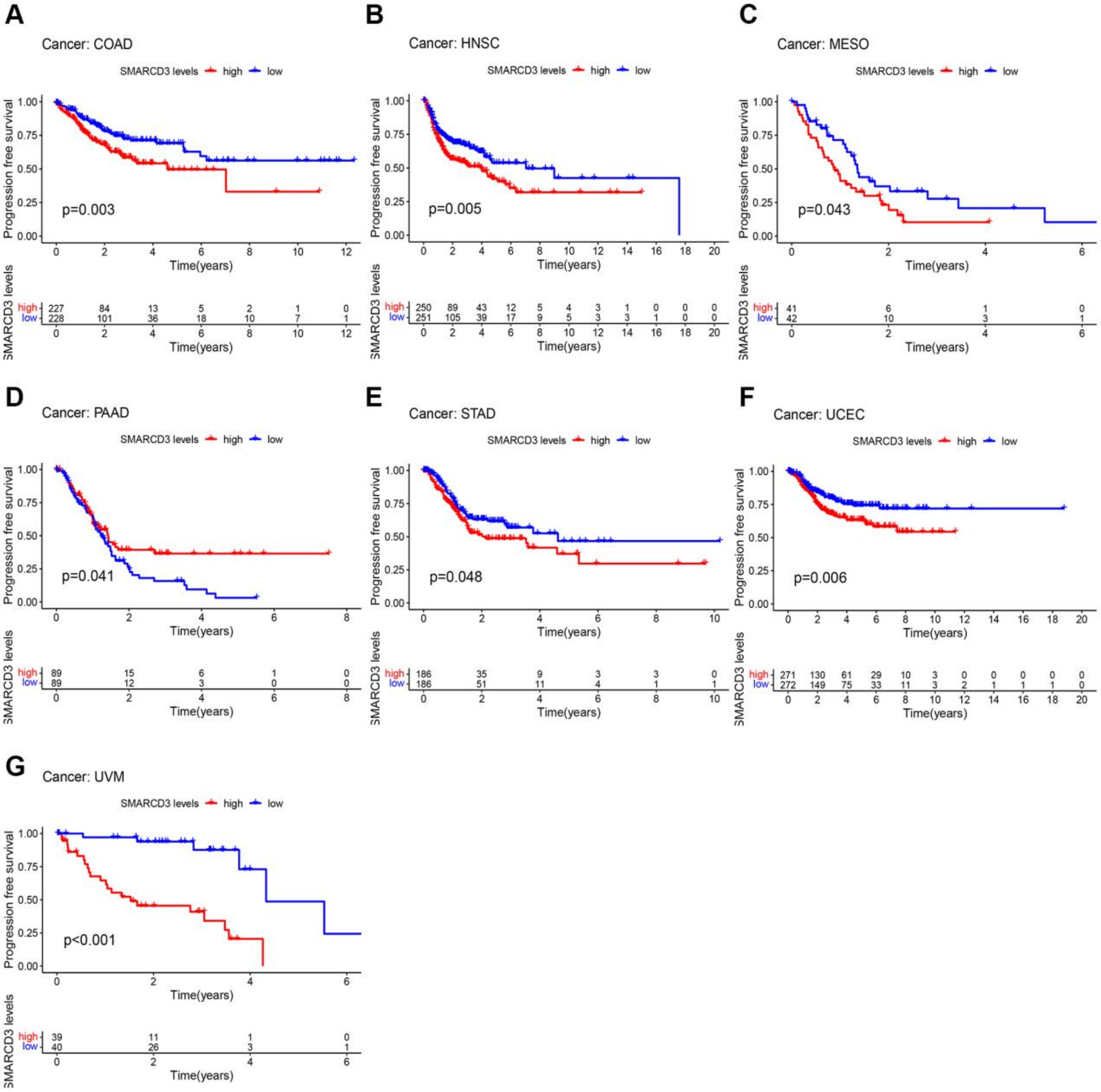

Supplementary Figure 7. KM survival curves based on progression-free survival in 7 cancers. (**A**) COAD; (**B**) HNSC; (**C**) MESO; (**D**) PAAD; (**E**) STAD; (**F**) UCEC; (**G**) UVM.

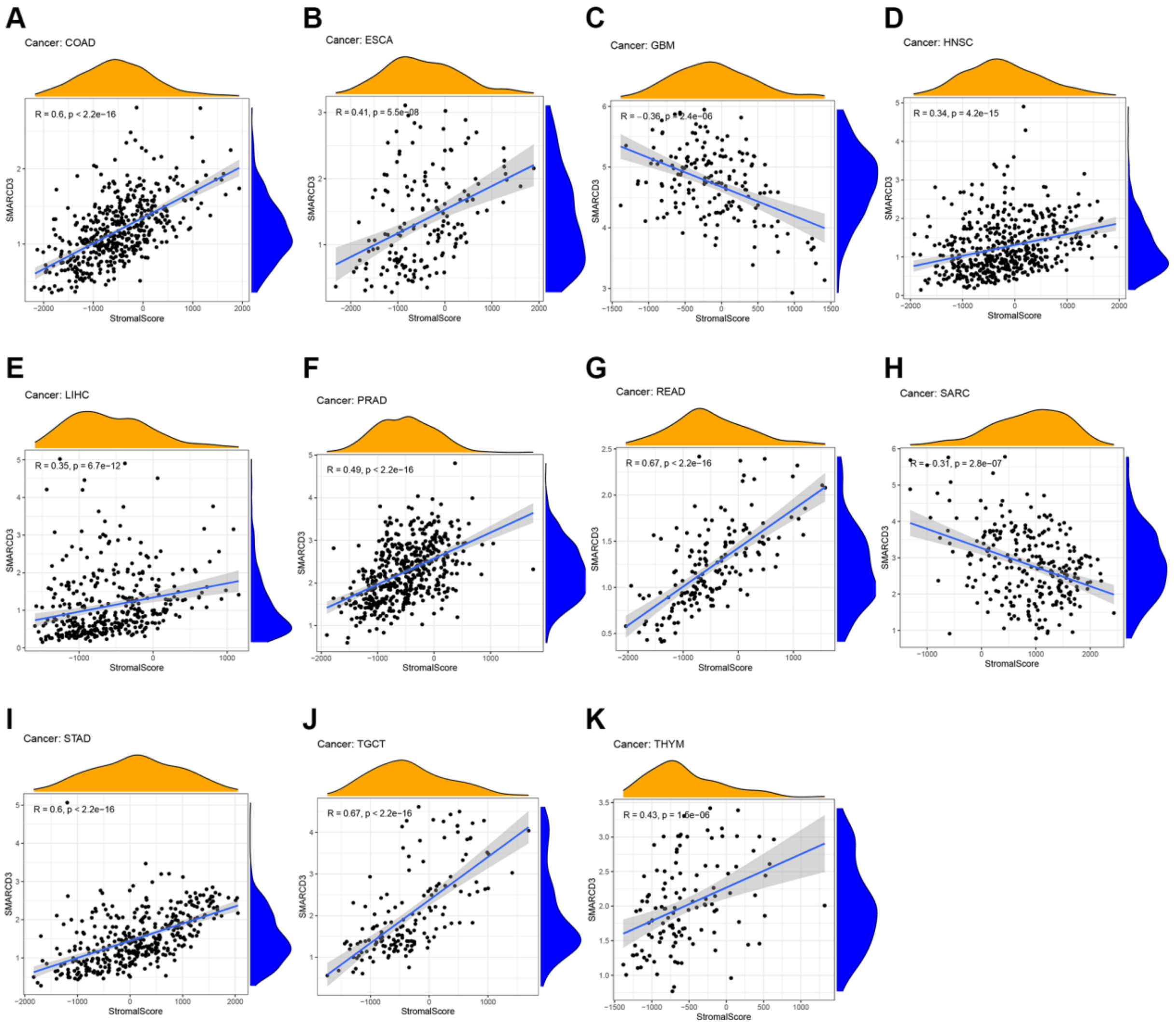

Supplementary Figure 8. Correlation between StromalScore and SMARCD3 expression in 11 cancers. (**A**) COAD; (**B**) ESCA; (**C**) GBM; (**D**) HNSC; (**E**) LIHC; (**F**) PRAD; (**G**) READ; (**H**) SARC; (**I**) STAD; (**J**) TGCT; (**K**) THYM.

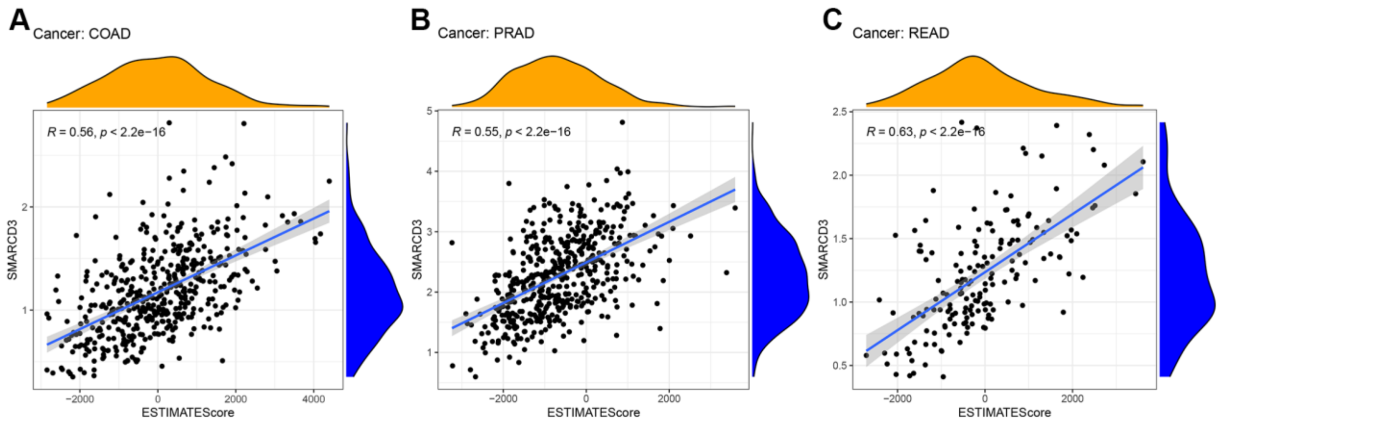

Supplementary Figure 9. Correlation between ESTIMATEScore and SMARCD3 expression in 3 cancers. (**A**) COAD; (**B**) PRAD; (**C**) READ.

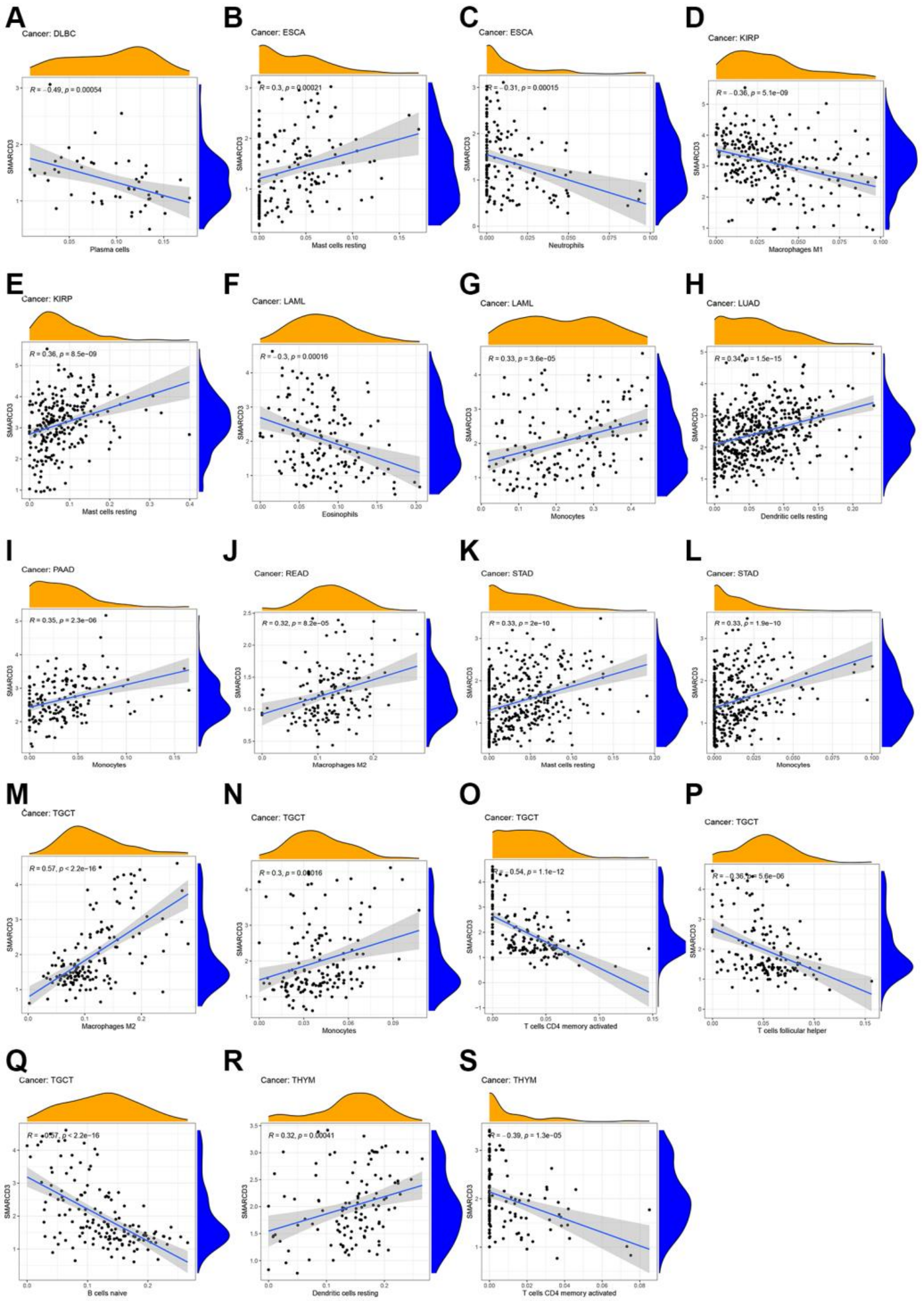

Supplementary Figure 10. SMARCD3-related immune cells in specific cancers. (**A**) Plasma cells in DLBC; (**B**) Mast cells resting in ESCA; (**C**) Neutrophils in ESCA; (**D**) Macrophages M1 in KIRP; (**E**) Mast cells resting in KIRC; (**F**) Eosinophils in LAML; (**G**) Monocytes in LAML; (**H**) Dendritic cells resting in LUAD; (**I**) Monocytes in PAAD; (**J**) Macrophages M2 in READ; (**K**) Mast cells resting in STAD; (**L**) Monocytes in STAD; (**M**) Macrophages M2 in TGCT; (**N**) Monocytes in TGCT; (**O**) T cells CD4 memory activated in TGCT; (**P**) T cells follicular helper in TGCT; (**Q**) B cells naive in TGCT; (**R**) Dendritic cells resting in THYM; (**S**) T cells CD4 memory activated in THYM.

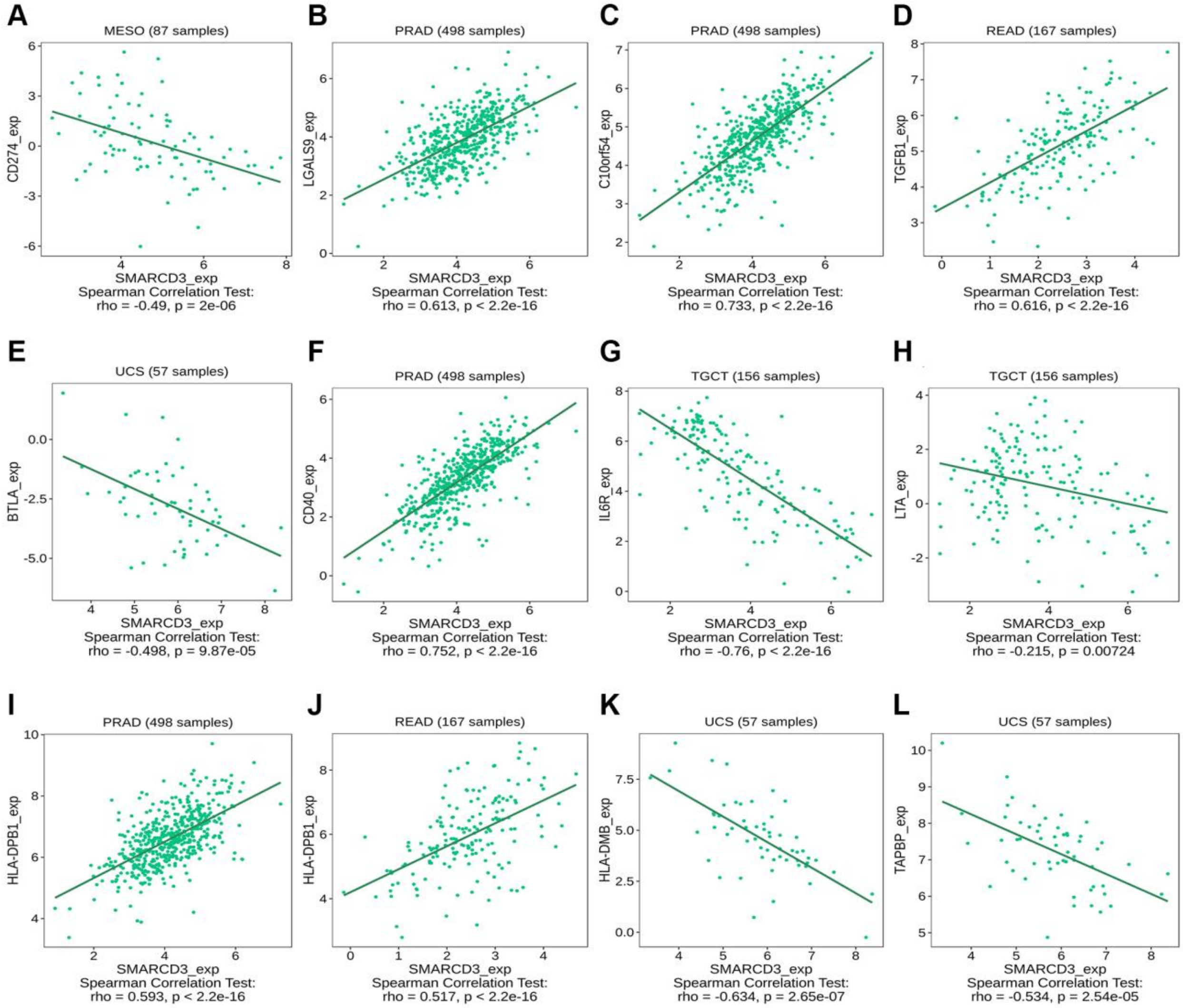

Supplementary Figure 11. Immune-related genes associated with SMARCD3 in specific cancers. (**A**–**E**) Immunoinhibitor type; (**F**–**H**) Immunostimulator type; (**I**–**L**) MHC type.

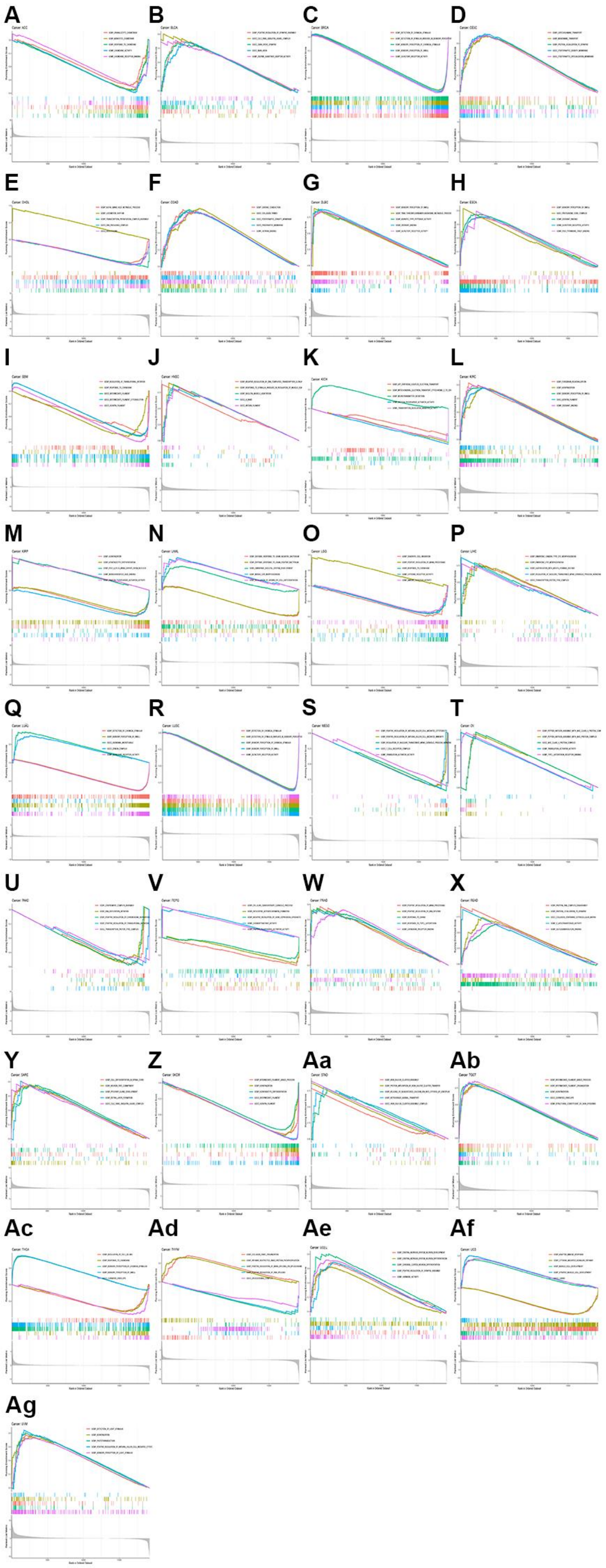

Supplementary Figure 12. Pan-cancer GSEA pathway enrichment analysis based on GO database. (**A**) ACC; (**B**) BLCA; (**C**) BRCA; (**D**) CESC; (**E**) CHOL; (**F**) COAD; (**G**) DLBC; (**H**) ESCA; (**I**) GBM; (**J**) HNSC; (**K**) KICH; (**L**) KIRC; (**M**) KIRP; (**N**) LAML; (**O**) LGG; (**P**) LIHC; (**Q**) LUAD; (**R**) LUSC; (**S**) MESO; (**T**) OV; (**U**) PAAD; (**V**) PCPG; (**W**) PRAD; (**X**) READ; (**Y**) SARC; (**Z**) SKCM; (**Aa**) STAD; (**Ab**) TGCT; (**Ac**) THCA; (**Ad**) THYM; (**Ae**) UCEC; (**Af**) UCS; (**Ag**) UVM.

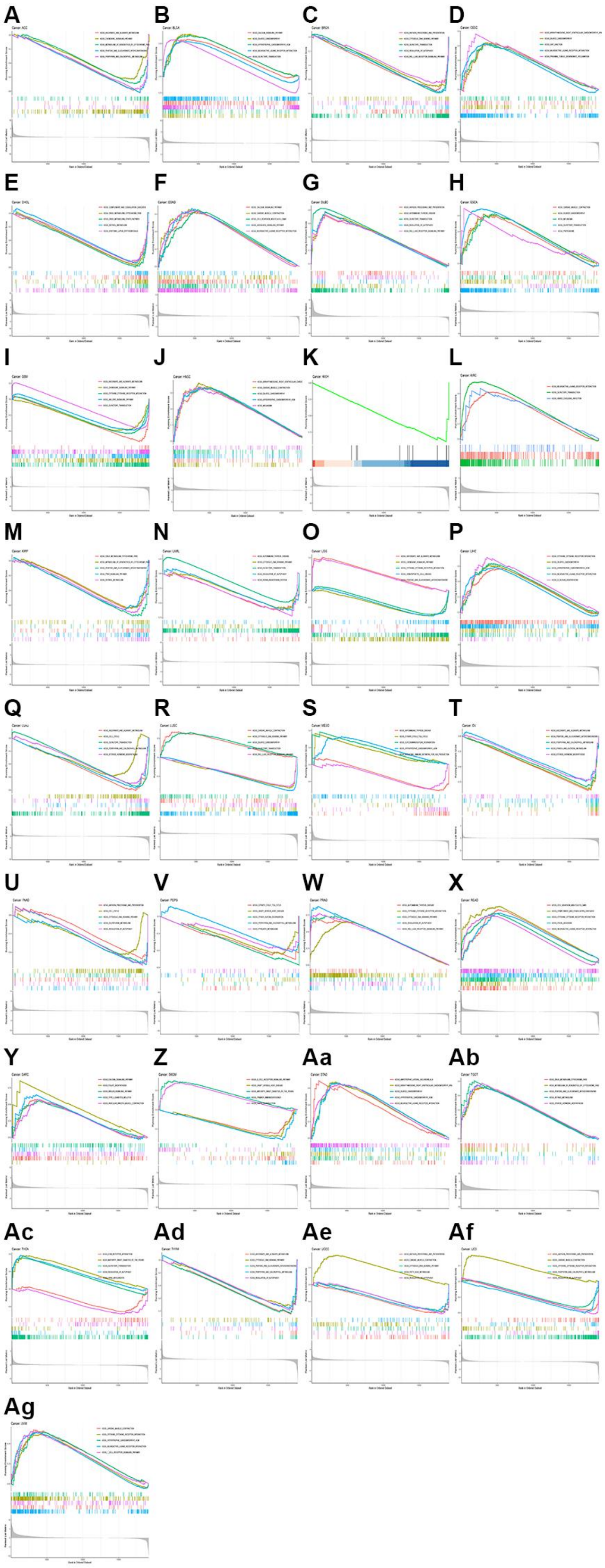

Supplementary Figure 13. Pan-cancer GSEA pathway enrichment analysis based on KEGG database. (**A**) ACC; (**B**) BLCA; (**C**) BRCA; (**D**) CESC; (**E**) CHOL; (**F**) COAD; (**G**) DLBC; (**H**) ESCA; (**I**) GBM; (**J**) HNSC; (**K**) KICH; (**L**) KIRC; (**M**) KIRP; (**N**) LAML; (**O**) LGG; (**P**) LIHC; (**Q**) LUAD; (**R**) LUSC; (**S**) MESO; (**T**) OV; (**U**) PAAD; (**V**) PCPG; (**W**) PRAD; (**X**) READ; (**Y**) SARC; (**Z**) SKCM; (**Aa**) STAD; (**Ab**) TGCT; (**Ac**) THCA; (**Ad**) THYM; (**Ae**) UCEC; (**Af**) UCS; (**Ag**) UVM.

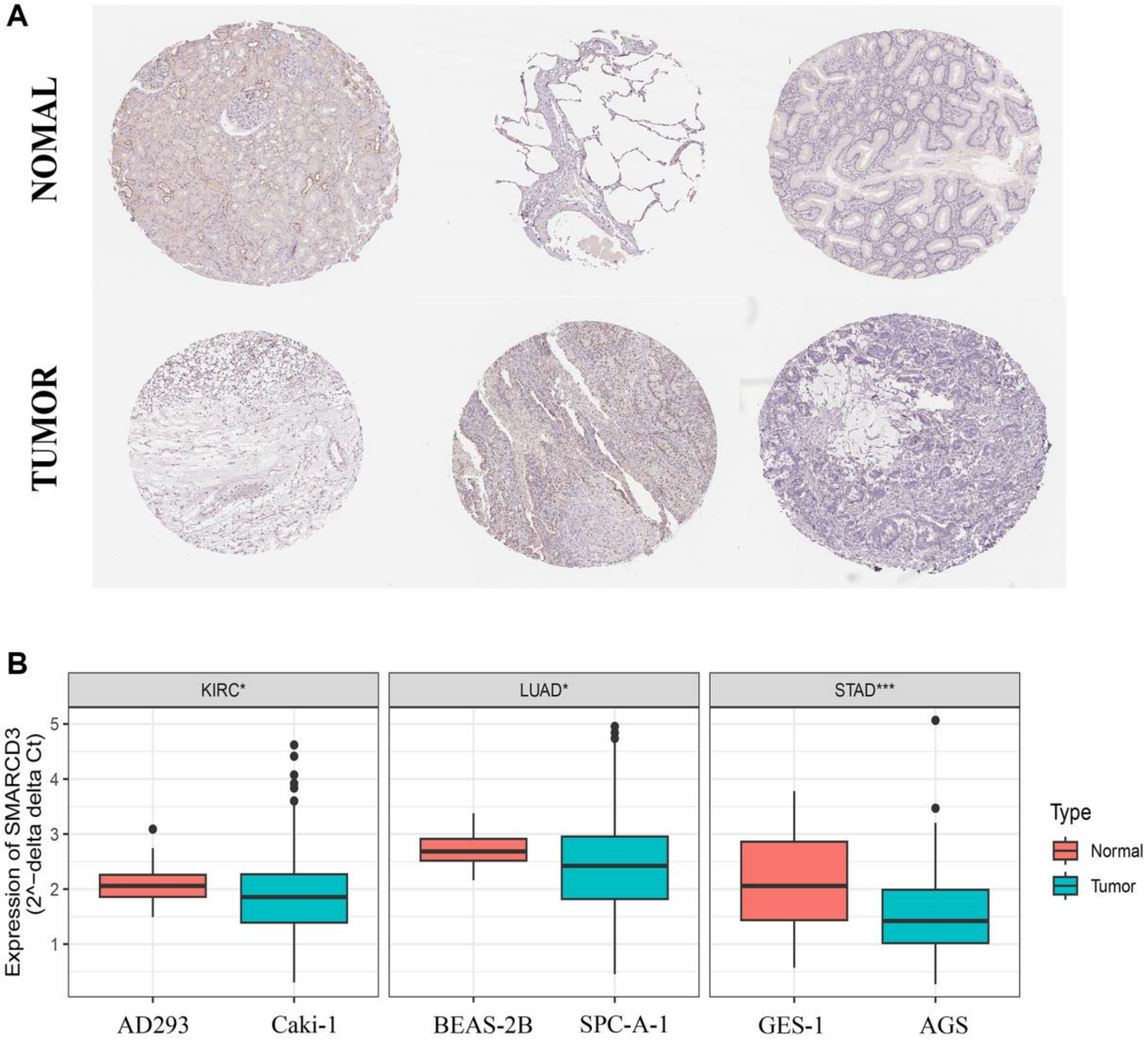

Supplementary Figure 14. Immunohistochemistry and RT-qPCR results of SMARCD3. (**A**) Immunohistochemical profile of SMARCD3; (**B**) SMARCD3 expression in KIRC, LUAD and STAD obtained by RT-qPCR analysis.

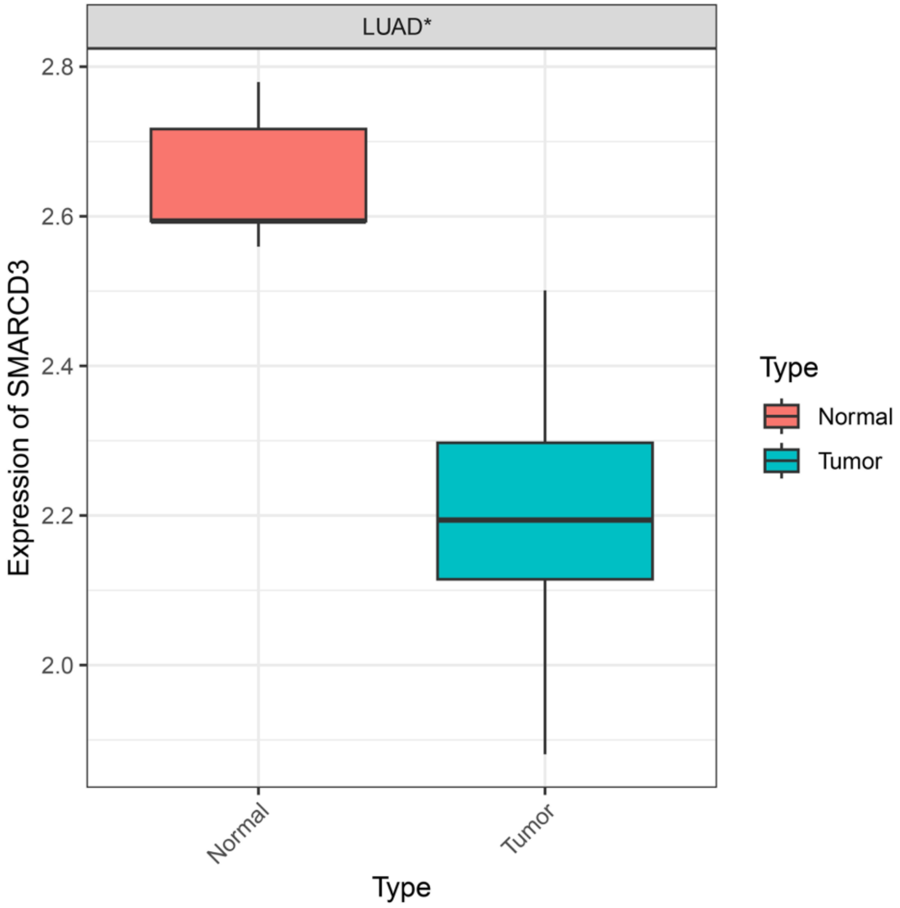

Supplementary Figure 15. Expression of SMARCD3 in tissues of LUAD patients.
