# Supplementary material for: Immunotherapy, prognostic, and tumor biomarker based on pancancer analysis, SMARCD3
Source: Aging (Albany NY). 2024 Jun 11;16(11):10074–107. doi: 10.18632/aging.205921 (PMC11210247; doi:10.18632/aging.205921)
Supplement: Supplementary Tables 1-5 and 8-9 [file aging-16-205921-s002.pdf]

SUPPLEMENTARY TABLES

Supplementary Table 1. Primer sequences.

Gene ID	Forward primer	Reverse primer
β-actin	CATCCGCAAAGACCTGTACG	CCTGCTTGCTGATCCACATC
SMARCD3	GGACGAAGTTGCCGGAGG	TGGGGCATCCGGGCT

Abbreviation: RT-qPCR: Real-time quantitative PCR.

Supplementary Table 2. Association of SMARCD3 with OS in 33 cancers by univariate COX analysis.

Cancer	HR	95% Low	95% High	*P*-value
ACC	0.60	0.38	0.92	0.02
BLCA	0.97	0.81	1.17	0.75
BRCA	0.81	0.66	0.99	0.04
CESC	1.02	0.73	1.41	0.91
CHOL	1.34	0.73	2.45	0.34
COAD	1.90	1.17	3.08	0.01
DLBC	0.93	0.21	4.21	0.93
ESCA	0.90	0.62	1.32	0.60
GBM	0.88	0.65	1.19	0.39
HNSC	1.17	0.97	1.42	0.10
KICH	1.39	0.39	4.95	0.61
KIRC	1.67	1.36	2.06	0.00
KIRP	0.78	0.54	1.11	0.16
LAML	0.88	0.70	1.10	0.28
LGG	0.65	0.45	0.94	0.02
LIHC	1.19	0.98	1.44	0.07
LUAD	0.75	0.62	0.91	0.00
LUSC	0.98	0.83	1.16	0.83
MESO	1.03	0.83	1.28	0.77
OV	1.00	0.88	1.14	0.98
PAAD	0.59	0.43	0.80	0.00
PCPG	2.46	1.08	5.63	0.03
PRAD	0.70	0.28	1.78	0.46
READ	1.49	0.67	3.29	0.32
SARC	0.98	0.80	1.20	0.84
SKCM	1.06	0.89	1.26	0.52
STAD	1.33	1.05	1.69	0.02
TGCT	1.02	0.41	2.53	0.97
THCA	0.84	0.29	2.38	0.74
THYM	1.62	0.50	5.24	0.42
UCEC	1.43	1.15	1.77	0.00
UCS	1.35	0.89	2.05	0.16
UVM	12.07	4.12	35.40	0.00

Abbreviations: ACC: adrenocortical cancer; BLCA: bladder urothelial carcinoma; BRCA: breast invasive carcinoma; CESC: cervical and endocervical cancer; CHOL: cholangiocarcinoma; COAD: colon adenocarcinoma; DLBC: diffuse large B-cell lymphoma; ESCA: esophageal carcinoma; GBM glioblastoma multiforme; GO: Gene Ontology; GSEA: Gene Set Enrichment; H: High; HNSC: head and neck squamous cell carcinoma; HR: Hazard Ratio; KEGG: Kyoto Encyclopedia of Genes and Genomes; KICH kidney chromophobe; KIRC: kidney clear cell carcinoma; KIRP: kidney papillary cell carcinoma; L: Low; LAML: acute myeloid leukemia;

LGG: brain lower grade glioma; LIHC: liver hepatocellular carcinoma; LUAD: lung adenocarcinoma; LUSC: lung squamous cell carcinoma; MESO: mesothelioma; MHC: major histocompatibility complex; OS: Overall Survival; OV: ovarian serous cystadenocarcinoma; PAAD: pancreatic adenocarcinoma; PCPG: pheochromocytoma and paraganglioma; PRAD: prostate adenocarcinoma; *P*-value: Probability; READ: rectum adenocarcinoma; SARC: sarcoma; SKCM: skin cutaneous melanoma; STAD: stomach adenocarcinoma; TGCT: testicular germ cell tumor; THCA thyroid carcinoma; THYM: thymoma; UCEC: uterine corpus endometrioid carcinoma; UCS uterine carcinosarcoma; UVM: uveal melanoma; 95% High: 95% confidence interval upper limit; 95% Low: 95% confidence interval lower limit.

Supplementary Table 3. Association of SMARCD3 with DFS in 33 cancers by univariate COX analysis.

Cancer	HR	95% Low	95% High	*P*-value
ACC	1.04	0.37	2.89	0.94
BLCA	1.07	0.66	1.72	0.79
BRCA	0.84	0.64	1.11	0.23
CESC	1.17	0.68	2.02	0.58
CHOL	1.77	0.82	3.82	0.14
COAD	0.92	0.33	2.59	0.88
DLBC	2.78	0.15	52.41	0.49
ESCA	1.38	0.81	2.35	0.24
HNSC	1.60	1.06	2.44	0.03
KICH	0.52	0.04	6.75	0.62
KIRC	0.47	0.18	1.21	0.12
KIRP	0.62	0.40	0.97	0.04
LGG	3.46	1.22	9.75	0.02
LIHC	1.01	0.83	1.24	0.90
LUAD	1.02	0.78	1.32	0.90
LUSC	0.95	0.70	1.30	0.75
MESO	1.10	0.47	2.60	0.82
OV	1.01	0.85	1.19	0.91
PAAD	0.55	0.29	1.05	0.07
PCPG	1.64	0.45	5.94	0.45
PRAD	1.20	0.69	2.10	0.52
READ	0.82	0.12	5.76	0.85
SARC	0.97	0.77	1.22	0.80
STAD	1.72	1.09	2.72	0.02
TGCT	1.07	0.77	1.49	0.68
THCA	0.55	0.24	1.25	0.16
UCEC	1.21	0.92	1.60	0.17
UCS	0.66	0.27	1.62	0.37

Abbreviations: ACC: adrenocortical cancer; BLCA: bladder urothelial carcinoma; BRCA: breast invasive carcinoma; CESC: cervical and endocervical cancer; CHOL: cholangiocarcinoma; COAD: colon adenocarcinoma; DFS: Disease Free Survival; DLBC: diffuse large B-cell lymphoma; ESCA: esophageal carcinoma; GBM glioblastoma multiforme; GO: Gene Ontology; GSEA: Gene Set Enrichment; H: High; HNSC: head and neck squamous cell carcinoma; HR: Hazard Ratio; KEGG: Kyoto Encyclopedia of Genes and Genomes; KICH kidney chromophobe; KIRC: kidney clear cell carcinoma; KIRP: kidney papillary cell carcinoma; L: Low; LAML: acute myeloid leukemia; LGG: brain lower grade glioma; LIHC: liver hepatocellular carcinoma; LUAD: lung adenocarcinoma; LUSC: lung squamous cell carcinoma; MESO: mesothelioma; MHC: major histocompatibility complex; OV: ovarian serous cystadenocarcinoma; PAAD: pancreatic adenocarcinoma; PCPG: pheochromocytoma and paraganglioma; PRAD: prostate adenocarcinoma; *P*-value: Probability; READ: rectum adenocarcinoma; SARC: sarcoma; SKCM: skin cutaneous melanoma; STAD: stomach adenocarcinoma; TGCT: testicular germ cell tumor; THCA thyroid carcinoma; THYM: thymoma; UCEC: uterine corpus endometrioid carcinoma; UCS uterine carcinosarcoma; UVM: uveal melanoma; 95% High: 95% confidence interval upper limit; 95% Low: 95% confidence interval lower limit.

Supplementary Table 4. Association of SMARCD3 with DSS in 33 cancers by univariate COX analysis.

Cancer	HR	95% Low	95% High	*P*-value
ACC	0.64	0.38	1.09	0.10
BLCA	0.99	0.80	1.24	0.96
BRCA	0.82	0.63	1.08	0.15
CESC	1.05	0.73	1.51	0.80
CHOL	1.53	0.79	2.95	0.21
COAD	2.20	1.28	3.78	0.00
DLBC	1.03	0.14	7.65	0.98
ESCA	1.28	0.84	1.95	0.26
GBM	0.85	0.62	1.17	0.32
HNSC	1.20	0.94	1.53	0.15
KICH	0.94	0.21	4.19	0.94
KIRC	1.74	1.34	2.26	0.00
KIRP	0.61	0.40	0.94	0.02
LGG	0.64	0.43	0.95	0.03
LIHC	1.16	0.90	1.49	0.26
LUAD	0.70	0.55	0.90	0.01
LUSC	0.87	0.66	1.14	0.30
MESO	1.04	0.80	1.35	0.76
OV	1.01	0.88	1.16	0.89
PAAD	0.58	0.41	0.82	0.00
PCPG	3.61	1.36	9.60	0.01
PRAD	0.61	0.14	2.60	0.50
READ	1.10	0.36	3.42	0.86
SARC	0.93	0.75	1.16	0.52
SKCM	1.12	0.92	1.35	0.26
STAD	1.66	1.23	2.23	0.00
TGCT	1.14	0.38	3.45	0.82
THCA	2.42	0.53	11.02	0.25
THYM	0.38	0.05	3.06	0.36
UCEC	1.48	1.14	1.92	0.00
UCS	1.47	0.90	2.41	0.13
UVM	14.55	4.61	45.89	0.00

Abbreviations: ACC: adrenocortical cancer; BLCA: bladder urothelial carcinoma; BRCA: breast invasive carcinoma; CESC: cervical and endocervical cancer; CHOL: cholangiocarcinoma; COAD: colon adenocarcinoma; DLBC: diffuse large B-cell lymphoma; DSS: Disease-specific survival; ESCA: esophageal carcinoma; GBM glioblastoma multiforme; GO: Gene Ontology; GSEA: Gene Set Enrichment; H: High; HNSC: head and neck squamous cell carcinoma; HR: Hazard Ratio; KEGG: Kyoto Encyclopedia of Genes and Genomes; KICH kidney chromophobe; KIRC: kidney clear cell carcinoma; KIRP: kidney papillary cell carcinoma; L: Low; LAML: acute myeloid leukemia; LGG: brain lower grade glioma; LIHC: liver hepatocellular carcinoma; LUAD: lung adenocarcinoma; LUSC: lung squamous cell carcinoma; MESO: mesothelioma; MHC: major histocompatibility complex; OV: ovarian serous cystadenocarcinoma; PAAD: pancreatic adenocarcinoma; PCPG: pheochromocytoma and paraganglioma; PRAD: prostate adenocarcinoma; *P*-value: Probability; READ: rectum adenocarcinoma; SARC: sarcoma; SKCM: skin cutaneous melanoma; STAD: stomach adenocarcinoma; TGCT: testicular germ cell tumor; THCA thyroid carcinoma; THYM: thymoma; UCEC: uterine corpus endometrioid carcinoma; UCS uterine carcinosarcoma; UVM: uveal melanoma; 95% High: 95% confidence interval upper limit; 95% Low: 95% confidence interval lower limit.

Supplementary Table 5. Association of SMARCD3 with PFS in 33 cancers by univariate COX analysis.

Cancer	HR	95% Low	95% High	*P*-value
ACC	0.79	0.54	1.17	0.24
BLCA	0.99	0.82	1.20	0.92
BRCA	0.91	0.74	1.12	0.36
CESC	1.25	0.93	1.69	0.13
CHOL	1.31	0.75	2.29	0.35
COAD	1.62	1.07	2.43	0.02
DLBC	0.87	0.23	3.26	0.84
ESCA	1.17	0.85	1.61	0.34
GBM	0.85	0.61	1.19	0.35
HNSC	1.32	1.09	1.60	0.01
KICH	1.41	0.44	4.54	0.56
KIRC	1.30	1.03	1.64	0.03
KIRP	0.64	0.47	0.88	0.01
LGG	0.89	0.65	1.23	0.47
LIHC	1.00	0.84	1.20	0.97
LUAD	0.87	0.73	1.04	0.11
LUSC	0.99	0.81	1.22	0.94
MESO	1.16	0.92	1.46	0.20
OV	1.01	0.90	1.13	0.86
PAAD	0.57	0.42	0.78	0.00
PCPG	2.59	1.56	4.29	0.00
PRAD	1.25	0.92	1.72	0.16
READ	1.01	0.50	2.05	0.97
SARC	0.90	0.76	1.07	0.23
SKCM	0.96	0.83	1.11	0.60
STAD	1.42	1.10	1.83	0.01
TGCT	1.10	0.82	1.48	0.52
THCA	1.11	0.63	1.96	0.71
THYM	0.58	0.25	1.33	0.20
UCEC	1.27	1.06	1.52	0.01
UCS	1.05	0.71	1.56	0.81
UVM	21.04	7.35	60.20	0.00

Abbreviations: ACC: adrenocortical cancer; BLCA: bladder urothelial carcinoma; BRCA: breast invasive carcinoma; CESC: cervical and endocervical cancer; CHOL: cholangiocarcinoma; COAD: colon adenocarcinoma; DLBC: diffuse large B-cell lymphoma; ESCA: esophageal carcinoma; GBM glioblastoma multiforme; GO: Gene Ontology; GSEA: Gene Set Enrichment; H: High; HNSC: head and neck squamous cell carcinoma; HR: Hazard Ratio; KEGG: Kyoto Encyclopedia of Genes and Genomes; KICH kidney chromophobe; KIRC: kidney clear cell carcinoma; KIRP: kidney papillary cell carcinoma; L: Low; LAML: acute myeloid leukemia; LGG: brain lower grade glioma; LIHC: liver hepatocellular carcinoma; LUAD: lung adenocarcinoma; LUSC: lung squamous cell carcinoma; MESO: mesothelioma; MHC: major histocompatibility complex; OS: Overall Survival; OV: ovarian serous cystadenocarcinoma; PAAD: pancreatic adenocarcinoma; PCPG: pheochromocytoma and paraganglioma; PFS: Progression Free Survival; PRAD: prostate adenocarcinoma; *P*-value: Probability; READ: rectum adenocarcinoma; SARC: sarcoma; SKCM: skin cutaneous melanoma; STAD: stomach adenocarcinoma; TGCT: testicular germ cell tumor; THCA thyroid carcinoma; THYM: thymoma; UCEC: uterine corpus endometrioid carcinoma; UCS uterine carcinosarcoma; UVM: uveal melanoma; 95% High: 95% confidence interval upper limit; 95% Low: 95% confidence interval lower limit.

Supplementary Table 8. Correlation analysis between SMARCD3 expression and TMB.

Cancer	Cor	*P*-value
ACC	−0.219213618	0.052253821
BLCA	−0.172776378	0.000455573
BRCA	−0.142354916	7.69397E-06
CESC	−0.046638393	0.432043114
CHOL	−0.030014182	0.862047362
COAD	−0.077877246	0.12181763
DLBC	−0.309862494	0.062414348
ESCA	−0.259936582	0.000901999
GBM	−0.00996257	0.904017777
HNSC	−0.046002095	0.308526128
KICH	0.030429996	0.809841984
KIRC	−0.190879488	0.000470381
KIRP	0.016018958	0.790313889
LAML	−0.219775032	0.023595958
LGG	0.090456267	0.042992969
LIHC	−0.215801783	3.73505E-05
LUAD	−0.307127942	1.89596E-12
LUSC	0.001764432	0.968987937
MESO	0.051882115	0.649761046
OV	−0.08188152	0.178151807
PAAD	−0.382028646	1.29634E-06
PCPG	0.098641222	0.191475741
PRAD	−0.326014321	2.12969E-13
READ	−0.081284564	0.354165172
SARC	−0.248509866	0.000118196
SKCM	−0.028678591	0.537311572
STAD	−0.416348802	7.33174E-17
TGCT	0.009174245	0.912791093
THCA	0.081747701	0.072961439
THYM	0.128545008	0.167206427
UCEC	−0.130559092	0.002724785
UCS	−0.131062915	0.335635046
UVM	−0.210780095	0.06055276

Abbreviations: ACC: adrenocortical cancer; BLCA: bladder urothelial carcinoma; BRCA: breast invasive carcinoma; CESC: cervical and endocervical cancer; CHOL: cholangiocarcinoma; COAD: colon adenocarcinoma; Cor: Correlation Coefficient; DLBC: diffuse large B-cell lymphoma; ESCA: esophageal carcinoma; GBM glioblastoma multiforme; HNSC: head and neck squamous cell carcinoma; KICH kidney chromophobe; KIRC: kidney clear cell carcinoma; KIRP: kidney papillary cell carcinoma; LAML: acute myeloid leukemia; LGG: brain lower grade glioma; LIHC: liver hepatocellular carcinoma; LUAD: lung adenocarcinoma; LUSC: lung squamous cell carcinoma; MESO: mesothelioma; MHC: major histocompatibility complex; OV: ovarian serous cystadenocarcinoma; PAAD: pancreatic adenocarcinoma; PCPG: pheochromocytoma and paraganglioma; PRAD: prostate adenocarcinoma; *P*-value: Probability; READ: rectum adenocarcinoma; SARC: sarcoma; SKCM: skin cutaneous melanoma; STAD: stomach adenocarcinoma; TGCT: testicular germ cell tumor; THCA thyroid carcinoma; THYM: thymoma; TMB: Tumor Mutational Burden. UCEC: uterine corpus endometrioid carcinoma; UCS uterine carcinosarcoma; UVM: uveal melanoma.

Supplementary Table 9. Correlation analysis between SMARCD3 expression and MSI.

Cancer	Cor	*P*-value
ACC	0.389427817	0.00038925
BLCA	−0.006666261	0.893212601
BRCA	0.066280138	0.032829853
CESC	0.082075055	0.154794435
CHOL	0.063577864	0.711744092
COAD	−0.019202283	0.692349301
DLBC	−0.115185236	0.435631652
ESCA	−0.070510215	0.375616548
GBM	−0.094619809	0.246242253
HNSC	0.07985558	0.075597959
KICH	0.226617566	0.069478634
KIRC	−0.108636567	0.046941524
KIRP	0.005971051	0.920057875
LAML	0.022714749	0.8112551
LGG	0.106366941	0.016471796
LIHC	−0.053040044	0.309570081
LUAD	0.012778586	0.773217965
LUSC	−0.043470362	0.335445197
MESO	0.084083463	0.452622256
OV	−0.064526651	0.288960264
PAAD	−0.190126665	0.011730639
PCPG	−0.019295516	0.798225431
PRAD	0.003344828	0.940827544
READ	−0.097405826	0.232545481
SARC	−0.058408013	0.354848418
SKCM	0.200464993	1.24499E-05
STAD	−0.300430545	3.06459E-09
TGCT	0.146437471	0.073754348
THCA	0.144220848	0.001353713
THYM	−0.003316277	0.971568932
UCEC	−0.006561196	0.879318285
UCS	0.097918589	0.472783895
UVM	0.189538462	0.092200351

Abbreviations: ACC: adrenocortical cancer; BLCA: bladder urothelial carcinoma; BRCA: breast invasive carcinoma; CESC: cervical and endocervical cancer; CHOL: cholangiocarcinoma; COAD: colon adenocarcinoma; Cor: Correlation Coefficient; DLBC: diffuse large B-cell lymphoma; ESCA: esophageal carcinoma; GBM glioblastoma multiforme; GO: Gene Ontology; GSEA: Gene Set Enrichment; HNSC: head and neck squamous cell carcinoma; KEGG: Kyoto Encyclopedia of Genes and Genomes; KICH kidney chromophobe; KIRC: kidney clear cell carcinoma; KIRP: kidney papillary cell carcinoma; LAML: acute myeloid leukemia; LGG: brain lower grade glioma; LIHC: liver hepatocellular carcinoma; LUAD: lung adenocarcinoma; LUSC: lung squamous cell carcinoma; MESO: mesothelioma; MHC: major histocompatibility complex; MSI: Microsatellite Instability; OV: ovarian serous cystadenocarcinoma; PAAD: pancreatic adenocarcinoma; PCPG: pheochromocytoma and paraganglioma; PRAD: prostate adenocarcinoma; *P*-value: Probability; READ: rectum adenocarcinoma; SARC: sarcoma; SKCM: skin cutaneous melanoma; STAD: stomach adenocarcinoma; TGCT: testicular germ cell tumor; THCA thyroid carcinoma; THYM: thymoma; UCEC: uterine corpus endometrioid carcinoma; UCS uterine carcinosarcoma; UVM: uveal melanoma.
