# Supplementary material for: Immunotherapy, prognostic, and tumor biomarker based on pancancer analysis, SMARCD3
Source: Aging (Albany NY). 2024 Jun 11;16(11):10074–107. doi: 10.18632/aging.205921 (PMC11210247; doi:10.18632/aging.205921)
Supplement: Supplementary Table 6 [file aging-16-205921-s003.docx]

**Supplementary Table 6. Screened GO database GSEA enriched pathways.**

| **ID** | **Enrichment Score** | **Cancer** |
| --- | --- | --- |
| GOBP_REGULATION OF TRANS SYNAPTIC SIGNALING | 0.446516091 | ACC |
| GOBP_RESPONSE TO CHEMOKINE | -0.759335567 | ACC |
| GOBP_NEUTROPHIL CHEMOTAXIS | -0.693675942 | ACC |
| GOBP_REGULATION OF CELL KILLING | -0.534426444 | ACC |
| GOBP_IMMUNE RESPONSE REGULATING CELL SURFACE RECEPTOR SIGNALING PATHWAY | -0.562306571 | ACC |
| GOBP_REGULATION OF MRNA SPLICING VIA SPLICEOSOME | 0.615533156 | ACC |
| GOMF_CHEMOKINE RECEPTOR BINDING | -0.715985362 | ACC |
| GOBP_INTERLEUKIN 12 PRODUCTION | -0.630624056 | ACC |
| GOBP_REGULATION OF ALPHA BETA T CELL ACTIVATION | -0.570137083 | ACC |
| GOBP_POSITIVE REGULATION OF LYMPHOCYTE ACTIVATION | -0.475984129 | ACC |
| GOCC_TRANSPORTER COMPLEX | 0.531882016 | BLCA |
| GOMF_VOLTAGE GATED CHANNEL ACTIVITY | 0.591635478 | BLCA |
| GOCC_INTERMEDIATE FILAMENT | 0.629814348 | BLCA |
| GOMF_TRANSLATION ACTIVATOR ACTIVITY | -0.943116199 | BLCA |
| GOBP_POSITIVE REGULATION OF TRANSLATIONAL INITIATION | -0.912328405 | BLCA |
| GOBP_ADENYLATE CYCLASE MODULATING G PROTEIN COUPLED RECEPTOR SIGNALING PATHWAY | 0.501345009 | BLCA |
| GOBP_POSITIVE REGULATION OF MRNA PROCESSING | 0.635984104 | BLCA |
| GOBP_POSITIVE REGULATION OF PHOSPHOLIPASE ACTIVITY | 0.651613509 | BLCA |
| GOBP_POSITIVE REGULATION OF CHROMOSOME SEGREGATION | -0.731365468 | BLCA |
| GOBP_CELL CYCLE DNA REPLICATION | -0.599457246 | BLCA |
| GOCC_INTERMEDIATE FILAMENT | -0.534007911 | BRCA |
| GOBP_CELL ACTIVATION INVOLVED IN IMMUNE RESPONSE | -0.435409629 | BRCA |
| GOBP_3 UTR MEDIATED MRNA STABILIZATION | -0.787639492 | BRCA |
| GOBP_IMMUNE RESPONSE REGULATING CELL SURFACE RECEPTOR SIGNALING PATHWAY | -0.420918899 | BRCA |
| GOBP_NATURAL KILLER CELL ACTIVATION INVOLVED IN IMMUNE RESPONSE | -0.73766765 | BRCA |
| GOBP_REGULATION OF LEUKOCYTE MEDIATED CYTOTOXICITY | -0.511320668 | BRCA |
| GOBP_NATURAL KILLER CELL ACTIVATION | -0.646428936 | BRCA |
| GOBP_B CELL PROLIFERATION | -0.640473174 | BRCA |
| GOBP_REGULATION OF NATURAL KILLER CELL MEDIATED IMMUNITY | -0.599427026 | BRCA |
| GOBP_REGULATION OF TRIGLYCERIDE METABOLIC PROCESS | 0.532804906 | BRCA |
| GOBP_REGULATION OF TRANS SYNAPTIC SIGNALING | 0.6181365 | CESC |
| GOCC_TRANSPORTER COMPLEX | 0.5793974 | CESC |
| GOMF_GATED CHANNEL ACTIVITY | 0.606836698 | CESC |
| GOBP_3 UTR MEDIATED MRNA STABILIZATION | 0.760272138 | CESC |
| GOBP_ADENYLATE CYCLASE MODULATING G PROTEIN COUPLED RECEPTOR SIGNALING PATHWAY | 0.584688685 | CESC |
| GOBP_GLUTAMATE RECEPTOR SIGNALING PATHWAY | 0.677544007 | CESC |
| GOBP_ADENYLATE CYCLASE ACTIVATING G PROTEIN COUPLED RECEPTOR SIGNALING PATHWAY | 0.600902626 | CESC |
| GOBP_REGULATION OF CELLULAR RESPONSE TO GROWTH FACTOR STIMULUS | 0.525611443 | CESC |
| GOBP_REGULATION OF TRANSMEMBRANE RECEPTOR PROTEIN SERINE THREONINE KINASE SIGNALING PATHWAY | 0.561878079 | CESC |
| GOBP_NEGATIVE REGULATION OF CELLULAR RESPONSE TO GROWTH FACTOR STIMULUS | 0.589313797 | CESC |
| GOCC_TRANSPORTER COMPLEX | 0.457745233 | CHOL |
| GOBP_POSITIVE REGULATION OF TRANSLATIONAL INITIATION | -0.845717087 | CHOL |
| GOMF_DNA BINDING TRANSCRIPTION ACTIVATOR ACTIVITY | 0.427523994 | CHOL |
| GOBP_REGULATION OF TRANSLATIONAL INITIATION | -0.748292449 | CHOL |
| GOBP_FATTY ACID METABOLIC PROCESS | -0.498337639 | CHOL |
| GOBP_REGULATION OF TRIGLYCERIDE METABOLIC PROCESS | -0.764959473 | CHOL |
| GOBP_TRIGLYCERIDE METABOLIC PROCESS | -0.685512449 | CHOL |
| GOBP_SMALL MOLECULE CATABOLIC PROCESS | -0.615681383 | CHOL |
| GOMF_NUCLEOSOMAL DNA BINDING | -0.823918278 | CHOL |
| GOBP_NEUTRAL LIPID METABOLIC PROCESS | -0.682384291 | CHOL |
| GOBP_POSITIVE REGULATION OF TRIGLYCERIDE METABOLIC PROCESS | -0.834245503 | CHOL |
| GOCC_TRANSPORTER COMPLEX | 0.598376523 | COAD |
| GOCC_KERATIN FILAMENT | 0.660559741 | COAD |
| GOBP_3 UTR MEDIATED MRNA STABILIZATION | 0.742198704 | COAD |
| GOBP_ADENYLATE CYCLASE MODULATING G PROTEIN COUPLED RECEPTOR SIGNALING PATHWAY | 0.633744115 | COAD |
| GOBP_NEUTROPHIL CHEMOTAXIS | 0.614905613 | COAD |
| GOBP_RESPONSE TO CHEMOKINE | 0.632110048 | COAD |
| GOBP_IMMUNE RESPONSE REGULATING CELL SURFACE RECEPTOR SIGNALING PATHWAY | 0.585676678 | COAD |
| GOBP_POSITIVE REGULATION OF T CELL PROLIFERATION | 0.598027792 | COAD |
| GOBP_LEUKOCYTE MEDIATED IMMUNITY | 0.571185686 | COAD |
| GOBP_REGULATION OF T CELL ACTIVATION | 0.577339613 | COAD |
| GOBP_KERATINOCYTE DIFFERENTIATION | 0.753590803 | DLBC |
| GOCC_INTERMEDIATE FILAMENT CYTOSKELETON | 0.749087817 | DLBC |
| GOMF_TRANSLATION ACTIVATOR ACTIVITY | 0.868199255 | DLBC |
| GOBP_REGULATION OF PEPTIDYL SERINE PHOSPHORYLATION OF STAT PROTEIN | 0.80735619 | DLBC |
| GOBP_POSITIVE REGULATION OF MRNA PROCESSING | 0.732833449 | DLBC |
| GOBP_REGULATION OF TRIGLYCERIDE METABOLIC PROCESS | 0.708596851 | DLBC |
| GOBP_INTERLEUKIN 17 MEDIATED SIGNALING PATHWAY | 0.875676957 | DLBC |
| GOBP_MRNA MODIFICATION | 0.766940528 | DLBC |
| GOCC_TRANSPORTER COMPLEX | 0.582438057 | ESCA |
| GOBP_REGULATION OF DEVELOPMENTAL GROWTH | 0.501460325 | ESCA |
| GOBP_DEVELOPMENTAL CELL GROWTH | 0.525680043 | ESCA |
| GOBP_REGULATION OF TRANSMEMBRANE RECEPTOR PROTEIN SERINE THREONINE KINASE SIGNALING PATHWAY | 0.527622178 | ESCA |
| GOBP_POSITIVE REGULATION OF MAPK CASCADE | 0.474365593 | ESCA |
| GOBP_NEGATIVE REGULATION OF TRANSMEMBRANE RECEPTOR PROTEIN SERINE THREONINE KINASE SIGNALING PATHWAY | 0.555355173 | ESCA |
| GOBP_NEGATIVE REGULATION OF TUMOR NECROSIS FACTOR SUPERFAMILY CYTOKINE PRODUCTION | 0.617084393 | ESCA |
| GOBP_POSITIVE REGULATION OF LIPASE ACTIVITY | 0.649430661 | ESCA |
| GOBP_NEGATIVE REGULATION OF DNA BIOSYNTHETIC PROCESS | 0.660026224 | ESCA |
| GOMF_DIACYLGLYCEROL KINASE ACTIVITY | 0.834713208 | ESCA |
| GOCC_INTERMEDIATE FILAMENT | -0.709824733 | GBM |
| GOCC_INTERMEDIATE FILAMENT CYTOSKELETON | -0.69823343 | GBM |
| GOBP_T CELL ACTIVATION INVOLVED IN IMMUNE RESPONSE | -0.610295881 | GBM |
| GOBP_POSITIVE REGULATION OF TRANSLATIONAL INITIATION | -0.853592854 | GBM |
| GOBP_3 UTR MEDIATED MRNA STABILIZATION | -0.82986289 | GBM |
| GOBP_RESPONSE TO CHEMOKINE | -0.716137337 | GBM |
| GOBP_REGULATION OF ALPHA BETA T CELL ACTIVATION | -0.603514227 | GBM |
| GOBP_ALPHA BETA T CELL ACTIVATION | -0.589810924 | GBM |
| GOBP_POSITIVE REGULATION OF LYMPHOCYTE ACTIVATION | -0.540028552 | GBM |
| GOBP_REGULATION OF CD4 POSITIVE ALPHA BETA T CELL ACTIVATION | -0.634420048 | GBM |
| GOCC_TRANSPORTER COMPLEX | 0.641636084 | HNSC |
| GOCC_ION CHANNEL COMPLEX | 0.655765448 | HNSC |
| GOBP_ADENYLATE CYCLASE MODULATING G PROTEIN COUPLED RECEPTOR SIGNALING PATHWAY | 0.600717798 | HNSC |
| GOBP_DEVELOPMENTAL GROWTH INVOLVED IN MORPHOGENESIS | 0.545541218 | HNSC |
| GOBP_REGULATION OF DEVELOPMENTAL GROWTH | 0.586089175 | HNSC |
| GOBP_REGULATION OF CELLULAR RESPONSE TO GROWTH FACTOR STIMULUS | 0.557607933 | HNSC |
| GOBP_REGULATION OF TRANSMEMBRANE RECEPTOR PROTEIN SERINE THREONINE KINASE SIGNALING PATHWAY | 0.568274319 | HNSC |
| GOBP_PHOSPHOLIPASE C ACTIVATING G PROTEIN COUPLED RECEPTOR SIGNALING PATHWAY | 0.623822349 | HNSC |
| GOBP_ERK1 AND ERK2 CASCADE | 0.520856333 | HNSC |
| GOMF_GROWTH FACTOR ACTIVITY | 0.629172817 | HNSC |
| GOBP_NEUTROPHIL MIGRATION | -0.562303922 | KICH |
| GOBP_POSITIVE REGULATION OF INFLAMMATORY RESPONSE | -0.516534568 | KICH |
| GOBP_GRANULOCYTE MIGRATION | -0.510723801 | KICH |
| GOBP_POSITIVE REGULATION OF EPITHELIAL CELL PROLIFERATION | -0.538387354 | KICH |
| GOCC_TRANSCRIPTION FACTOR TFIID COMPLEX | -0.744353533 | KICH |
| GOBP_REGULATION OF INFLAMMATORY RESPONSE | -0.480225917 | KICH |
| GOCC_BBAF COMPLEX | 0.898310648 | KICH |
| GOMF_INTERLEUKIN 1 RECEPTOR BINDING | -0.85326086 | KICH |
| GOBP_CHROMOSOME CONDENSATION | -0.704963087 | KICH |
| GOMF_GROWTH FACTOR RECEPTOR BINDING | -0.555383451 | KICH |
| GOCC_TRANSPORTER COMPLEX | 0.597790481 | KIRC |
| GOCC_ION CHANNEL COMPLEX | 0.620891536 | KIRC |
| GOCC_INTERMEDIATE FILAMENT CYTOSKELETON | 0.726765789 | KIRC |
| GOCC_INTERMEDIATE FILAMENT | 0.743845546 | KIRC |
| GOBP_ADENYLATE CYCLASE MODULATING G PROTEIN COUPLED RECEPTOR SIGNALING PATHWAY | 0.600394578 | KIRC |
| GOBP_INTERMEDIATE FILAMENT ORGANIZATION | 0.693148421 | KIRC |
| GOBP_ADENYLATE CYCLASE ACTIVATING G PROTEIN COUPLED RECEPTOR SIGNALING PATHWAY | 0.663179472 | KIRC |
| GOBP_G PROTEIN COUPLED RECEPTOR SIGNALING PATHWAY COUPLED TO CYCLIC NUCLEOTIDE SECOND MESSENGER | 0.676019383 | KIRC |
| GOBP_POSITIVE REGULATION OF RNA SPLICING | 0.758135316 | KIRC |
| GOBP_POSITIVE REGULATION OF MRNA SPLICING VIA SPLICEOSOME | 0.788969266 | KIRC |
| GOMF_TRANSLATION ACTIVATOR ACTIVITY | -0.845092358 | KIRP |
| GOBP_3 UTR MEDIATED MRNA STABILIZATION | -0.807219898 | KIRP |
| GOBP_INTERMEDIATE FILAMENT BASED PROCESS | -0.564957322 | KIRP |
| GOBP_INTERMEDIATE FILAMENT ORGANIZATION | -0.601177412 | KIRP |
| GOMF_CHEMOKINE RECEPTOR BINDING | -0.602228476 | KIRP |
| GOBP_REGULATION OF TRANSLATIONAL INITIATION | -0.56971202 | KIRP |
| GOBP_ARACHIDONIC ACID METABOLIC PROCESS | -0.583147999 | KIRP |
| GOBP_REGULATION OF TRIGLYCERIDE METABOLIC PROCESS | -0.639160418 | KIRP |
| GOBP_LONG CHAIN FATTY ACID METABOLIC PROCESS | -0.5281515 | KIRP |
| GOBP_LIPID CATABOLIC PROCESS | -0.50961047 | KIRP |
| GOBP_NEUTROPHIL CHEMOTAXIS | 0.487289542 | LAML |
| GOBP_GRANULOCYTE CHEMOTAXIS | 0.432355587 | LAML |
| GOBP_CELLULAR RESPONSE TO INTERLEUKIN 1 | 0.423652436 | LAML |
| GOBP_POSITIVE REGULATION OF TUMOR NECROSIS FACTOR SUPERFAMILY CYTOKINE PRODUCTION | 0.489057536 | LAML |
| GOBP_LEUKOCYTE APOPTOTIC PROCESS | 0.451710713 | LAML |
| GOBP_REGULATION OF LEUKOCYTE APOPTOTIC PROCESS | 0.475047025 | LAML |
| GOBP_POSITIVE REGULATION OF REGULATORY T CELL DIFFERENTIATION | 0.631844259 | LAML |
| GOBP_DENDRITIC CELL ANTIGEN PROCESSING AND PRESENTATION | 0.823944755 | LAML |
| GOBP_REGULATION OF DENDRITIC CELL ANTIGEN PROCESSING AND PRESENTATION | 0.855435068 | LAML |
| GOMF_LIPASE ACTIVITY | 0.421290384 | LAML |
| GOBP_POSITIVE REGULATION OF CELL CELL ADHESION | -0.538076105 | LGG |
| GOCC_INTERMEDIATE FILAMENT | 0.512498353 | LGG |
| GOBP_POSITIVE REGULATION OF TRANSLATIONAL INITIATION | 0.698790808 | LGG |
| GOBP_3 UTR MEDIATED MRNA STABILIZATION | 0.718604623 | LGG |
| GOBP_NEUTROPHIL MIGRATION | -0.627590589 | LGG |
| GOBP_GRANULOCYTE CHEMOTAXIS | -0.645306716 | LGG |
| GOBP_POSITIVE REGULATION OF T CELL PROLIFERATION | -0.612809361 | LGG |
| GOBP_ALPHA BETA T CELL ACTIVATION | -0.582047779 | LGG |
| GOBP_POSITIVE REGULATION OF LYMPHOCYTE ACTIVATION | -0.549299492 | LGG |
| GOBP_POSITIVE REGULATION OF LIPASE ACTIVITY | -0.60222408 | LGG |
| GOCC_TRANSPORTER COMPLEX | 0.589396817 | LIHC |
| GOCC_ION CHANNEL COMPLEX | 0.602977324 | LIHC |
| GOBP_ADENYLATE CYCLASE MODULATING G PROTEIN COUPLED RECEPTOR SIGNALING PATHWAY | 0.523086391 | LIHC |
| GOBP_INTERMEDIATE FILAMENT ORGANIZATION | 0.655647316 | LIHC |
| GOBP_ADENYLATE CYCLASE ACTIVATING G PROTEIN COUPLED RECEPTOR SIGNALING PATHWAY | 0.540272976 | LIHC |
| GOBP_REGULATION OF CELL KILLING | 0.558859471 | LIHC |
| GOBP_NEUTROPHIL CHEMOTAXIS | 0.562457919 | LIHC |
| GOBP_PHOSPHOLIPASE C ACTIVATING G PROTEIN COUPLED RECEPTOR SIGNALING PATHWAY | 0.553931103 | LIHC |
| GOBP_ADENYLATE CYCLASE INHIBITING G PROTEIN COUPLED RECEPTOR SIGNALING PATHWAY | 0.591791757 | LIHC |
| GOMF_DNA BINDING TRANSCRIPTION ACTIVATOR ACTIVITY | 0.522536266 | LIHC |
| GOCC_INTERMEDIATE FILAMENT | -0.576977082 | LUAD |
| GOCC_INTERMEDIATE FILAMENT CYTOSKELETON | -0.571978962 | LUAD |
| GOMF_TRANSLATION ACTIVATOR ACTIVITY | -0.899790471 | LUAD |
| GOBP_3 UTR MEDIATED MRNA STABILIZATION | -0.864567256 | LUAD |
| GOBP_POSITIVE REGULATION OF TRANSLATIONAL INITIATION | -0.840455912 | LUAD |
| GOBP_INTERMEDIATE FILAMENT BASED PROCESS | -0.623007519 | LUAD |
| GOBP_REGULATION OF TRANSLATIONAL INITIATION | -0.671452326 | LUAD |
| GOBP_NEGATIVE REGULATION OF MRNA METABOLIC PROCESS | -0.70623416 | LUAD |
| GOBP_CELL CYCLE DNA REPLICATION | -0.653922249 | LUAD |
| GOBP_RESPONSE TO MACROPHAGE COLONY STIMULATING FACTOR | 0.710664951 | LUAD |
| GOMF_GATED CHANNEL ACTIVITY | 0.530889323 | LUSC |
| GOCC_INTERMEDIATE FILAMENT | -0.727158157 | LUSC |
| GOCC_INTERMEDIATE FILAMENT CYTOSKELETON | -0.704160687 | LUSC |
| GOMF_TRANSLATION ACTIVATOR ACTIVITY | -0.928619302 | LUSC |
| GOBP_POSITIVE REGULATION OF TRANSLATIONAL INITIATION | -0.89990054 | LUSC |
| GOBP_3 UTR MEDIATED MRNA STABILIZATION | -0.864532935 | LUSC |
| GOBP_ADENYLATE CYCLASE MODULATING G PROTEIN COUPLED RECEPTOR SIGNALING PATHWAY | 0.472607357 | LUSC |
| GOBP_PHOSPHOLIPASE C ACTIVATING G PROTEIN COUPLED RECEPTOR SIGNALING PATHWAY | 0.546977267 | LUSC |
| GOBP_NATURAL KILLER CELL ACTIVATION INVOLVED IN IMMUNE RESPONSE | -0.831337849 | LUSC |
| GOBP_NEGATIVE REGULATION OF MRNA METABOLIC PROCESS | -0.683918753 | LUSC |
| GOCC_TRANSPORTER COMPLEX | 0.579953017 | MESO |
| GOMF_TRANSLATION ACTIVATOR ACTIVITY | -0.860006594 | MESO |
| GOBP_POSITIVE REGULATION OF TRANSLATIONAL INITIATION | -0.761804215 | MESO |
| GOBP_3 UTR MEDIATED MRNA STABILIZATION | -0.756821766 | MESO |
| GOMF_DNA BINDING TRANSCRIPTION ACTIVATOR ACTIVITY | 0.576837942 | MESO |
| GOBP_REGULATION OF T HELPER 1 TYPE IMMUNE RESPONSE | -0.773057983 | MESO |
| GOBP_POSITIVE REGULATION OF NATURAL KILLER CELL MEDIATED IMMUNITY | -0.872289484 | MESO |
| GOBP_NEGATIVE REGULATION OF NATURAL KILLER CELL MEDIATED IMMUNITY | -0.808025441 | MESO |
| GOCC_T CELL RECEPTOR COMPLEX | -0.903614349 | MESO |
| GOBP_CD4 POSITIVE ALPHA BETA T CELL PROLIFERATION | -0.76053188 | MESO |
| GOBP_POSITIVE REGULATION OF TRANSLATIONAL INITIATION | 0.780870578 | OV |
| GOBP_3 UTR MEDIATED MRNA STABILIZATION | 0.804460257 | OV |
| GOMF_TRANSLATION ACTIVATOR ACTIVITY | 0.865230885 | OV |
| GOMF_DNA BINDING TRANSCRIPTION ACTIVATOR ACTIVITY | -0.459874998 | OV |
| GOBP_G PROTEIN COUPLED RECEPTOR SIGNALING PATHWAY COUPLED TO CYCLIC NUCLEOTIDE SECOND MESSENGER | -0.654343808 | OV |
| GOBP_NEGATIVE REGULATION OF NATURAL KILLER CELL MEDIATED IMMUNITY | 0.521994506 | OV |
| GOBP_POSITIVE REGULATION OF T HELPER CELL DIFFERENTIATION | 0.62954873 | OV |
| GOMF_MHC CLASS I RECEPTOR ACTIVITY | 0.686994201 | OV |
| GOCC_MHC CLASS II PROTEIN COMPLEX | 0.870852117 | OV |
| GOBP_LONG CHAIN FATTY ACID TRANSPORT | -0.583593099 | OV |
| GOCC_TRANSPORTER COMPLEX | 0.605430327 | PAAD |
| GOCC_ION CHANNEL COMPLEX | 0.62965584 | PAAD |
| GOCC_INTERMEDIATE FILAMENT CYTOSKELETON | 0.587142338 | PAAD |
| GOCC_INTERMEDIATE FILAMENT | 0.606046741 | PAAD |
| GOMF_TRANSLATION ACTIVATOR ACTIVITY | -0.861714433 | PAAD |
| GOBP_POSITIVE REGULATION OF TRANSLATIONAL INITIATION | -0.780934347 | PAAD |
| GOBP_3 UTR MEDIATED MRNA STABILIZATION | -0.659952266 | PAAD |
| GOBP_ADENYLATE CYCLASE MODULATING G PROTEIN COUPLED RECEPTOR SIGNALING PATHWAY | 0.604291774 | PAAD |
| GOBP_CELL CYCLE DNA REPLICATION | -0.607633265 | PAAD |
| GOBP_ANTIGEN PROCESSING AND PRESENTATION OF PEPTIDE ANTIGEN VIA MHC CLASS IB | -0.743610098 | PAAD |
| GOMF_CHEMOKINE RECEPTOR BINDING | 0.650526661 | PCPG |
| GOMF_CCR CHEMOKINE RECEPTOR BINDING | 0.734864294 | PCPG |
| GOBP_NATURAL KILLER CELL MEDIATED IMMUNITY | -0.585065433 | PCPG |
| GOBP_POSITIVE CHEMOTAXIS | 0.711918034 | PCPG |
| GOBP_DNA METHYLATION DEPENDENT HETEROCHROMATIN FORMATION | -0.754929192 | PCPG |
| GOBP_ARACHIDONIC ACID METABOLIC PROCESS | 0.688588673 | PCPG |
| GOMF_ARACHIDONIC ACID MONOOXYGENASE ACTIVITY | 0.800819635 | PCPG |
| GOBP_LONG CHAIN FATTY ACID METABOLIC PROCESS | 0.61982562 | PCPG |
| GOBP_MEIOTIC CELL CYCLE PHASE TRANSITION | 0.852194778 | PCPG |
| GOMF_DNA BINDING TRANSCRIPTION REPRESSOR ACTIVITY | 0.534349796 | PCPG |
| GOCC_TRANSPORTER COMPLEX | 0.514257714 | PRAD |
| GOCC_ION CHANNEL COMPLEX | 0.53620034 | PRAD |
| GOCC_INTERMEDIATE FILAMENT | 0.513293717 | PRAD |
| GOBP_ADENYLATE CYCLASE MODULATING G PROTEIN COUPLED RECEPTOR SIGNALING PATHWAY | 0.549799816 | PRAD |
| GOBP_INTERMEDIATE FILAMENT BASED PROCESS | 0.589581157 | PRAD |
| GOBP_NEUTROPHIL MIGRATION | 0.631495182 | PRAD |
| GOBP_REGULATION OF CELL KILLING | 0.57820763 | PRAD |
| GOBP_RESPONSE TO CHEMOKINE | 0.662992914 | PRAD |
| GOBP_POSITIVE REGULATION OF T CELL PROLIFERATION | 0.619434946 | PRAD |
| GOBP_UNSATURATED FATTY ACID METABOLIC PROCESS | 0.582403085 | PRAD |
| GOCC_TRANSPORTER COMPLEX | 0.503879798 | READ |
| GOCC_ION CHANNEL COMPLEX | 0.514681456 | READ |
| GOBP_CELL ACTIVATION INVOLVED IN IMMUNE RESPONSE | 0.533249183 | READ |
| GOBP_ADENYLATE CYCLASE MODULATING G PROTEIN COUPLED RECEPTOR SIGNALING PATHWAY | 0.5506373 | READ |
| GOBP_NEUTROPHIL CHEMOTAXIS | 0.638709033 | READ |
| GOBP_RESPONSE TO CHEMOKINE | 0.652300607 | READ |
| GOBP_POSITIVE REGULATION OF T CELL PROLIFERATION | 0.579501726 | READ |
| GOBP_ALPHA BETA T CELL ACTIVATION | 0.536002507 | READ |
| GOBP_LEUKOCYTE MIGRATION | 0.531166035 | READ |
| GOBP_FATTY ACID METABOLIC PROCESS | 0.523880025 | READ |
| GOCC_TRANSPORTER COMPLEX | 0.472156696 | SARC |
| GOCC_ION CHANNEL COMPLEX | 0.483916587 | SARC |
| GOCC_INTERMEDIATE FILAMENT | 0.547028014 | SARC |
| GOBP_POSITIVE REGULATION OF TRANSLATIONAL INITIATION | 0.738216925 | SARC |
| GOBP_3 UTR MEDIATED MRNA STABILIZATION | 0.756979148 | SARC |
| GOBP_ADENYLATE CYCLASE MODULATING G PROTEIN COUPLED RECEPTOR SIGNALING PATHWAY | 0.529148283 | SARC |
| GOMF_DNA BINDING TRANSCRIPTION ACTIVATOR ACTIVITY | 0.566595871 | SARC |
| GOBP_REGULATION OF T HELPER 1 TYPE IMMUNE RESPONSE | -0.800364965 | SARC |
| GOBP_NEGATIVE REGULATION OF INTERLEUKIN 12 PRODUCTION | -0.79131148 | SARC |
| GOBP_POSITIVE REGULATION OF FATTY ACID BETA OXIDATION | 0.754757108 | SARC |
| GOCC_TRANSPORTER COMPLEX | 0.446314065 | SKCM |
| GOCC_ION CHANNEL COMPLEX | 0.472898398 | SKCM |
| GOCC_INTERMEDIATE FILAMENT | -0.881643303 | SKCM |
| GOBP_T CELL ACTIVATION INVOLVED IN IMMUNE RESPONSE | -0.558283965 | SKCM |
| GOBP_INTERMEDIATE FILAMENT BASED PROCESS | -0.812038896 | SKCM |
| GOBP_INTERMEDIATE FILAMENT ORGANIZATION | -0.831283802 | SKCM |
| GOBP_POSITIVE REGULATION OF T CELL PROLIFERATION | -0.613254985 | SKCM |
| GOMF_IMMUNE RECEPTOR ACTIVITY | -0.612694376 | SKCM |
| GOBP_IMMUNE RESPONSE REGULATING CELL SURFACE RECEPTOR SIGNALING PATHWAY | -0.555957203 | SKCM |
| GOBP_REGULATION OF CD4 POSITIVE ALPHA BETA T CELL ACTIVATION | -0.611193598 | SKCM |
| GOCC_TRANSPORTER COMPLEX | 0.618338033 | STAD |
| GOCC_ION CHANNEL COMPLEX | 0.64630938 | STAD |
| GOBP_ADENYLATE CYCLASE MODULATING G PROTEIN COUPLED RECEPTOR SIGNALING PATHWAY | 0.619474699 | STAD |
| GOBP_REGULATION OF TRANSMEMBRANE RECEPTOR PROTEIN SERINE THREONINE KINASE SIGNALING PATHWAY | 0.562253338 | STAD |
| GOBP_PHOSPHOLIPASE C ACTIVATING G PROTEIN COUPLED RECEPTOR SIGNALING PATHWAY | 0.608922749 | STAD |
| GOBP_REGULATION OF MRNA PROCESSING | 0.546098453 | STAD |
| GOBP_G PROTEIN COUPLED RECEPTOR SIGNALING PATHWAY COUPLED TO CYCLIC NUCLEOTIDE SECOND MESSENGER | 0.633381415 | STAD |
| GOBP_REGULATION OF CANONICAL WNT SIGNALING PATHWAY | 0.536615872 | STAD |
| GOBP_REGULATION OF PHOSPHOLIPASE C ACTIVITY | 0.630476648 | STAD |
| GOBP_POSITIVE REGULATION OF LIPID METABOLIC PROCESS | 0.53690494 | STAD |
| GOCC_TRANSPORTER COMPLEX | 0.504658633 | TGCT |
| GOCC_ION CHANNEL COMPLEX | 0.516924297 | TGCT |
| GOCC_INTERMEDIATE FILAMENT | 0.63809339 | TGCT |
| GOBP_ADENYLATE CYCLASE MODULATING G PROTEIN COUPLED RECEPTOR SIGNALING PATHWAY | 0.581693971 | TGCT |
| GOBP_NEUTROPHIL MIGRATION | 0.565092679 | TGCT |
| GOBP_ADENYLATE CYCLASE INHIBITING G PROTEIN COUPLED RECEPTOR SIGNALING PATHWAY | 0.60683983 | TGCT |
| GOBP_PHOSPHOLIPASE C ACTIVATING G PROTEIN COUPLED RECEPTOR SIGNALING PATHWAY | 0.620022175 | TGCT |
| GOMF_DNA BINDING TRANSCRIPTION ACTIVATOR ACTIVITY | 0.533631786 | TGCT |
| GOBP_FATTY ACID METABOLIC PROCESS | 0.508187197 | TGCT |
| GOBP_TRIGLYCERIDE METABOLIC PROCESS | 0.607794212 | TGCT |
| GOCC_INTERMEDIATE FILAMENT CYTOSKELETON | 0.59651386 | THCA |
| GOCC_INTERMEDIATE FILAMENT | 0.616390021 | THCA |
| GOBP_NEUTROPHIL MIGRATION | -0.522814094 | THCA |
| GOBP_REGULATION OF CELL KILLING | -0.635821155 | THCA |
| GOBP_NEUTROPHIL CHEMOTAXIS | -0.558075009 | THCA |
| GOBP_ADENYLATE CYCLASE ACTIVATING G PROTEIN COUPLED RECEPTOR SIGNALING PATHWAY | -0.404248912 | THCA |
| GOBP_POSITIVE REGULATION OF T CELL PROLIFERATION | -0.487174066 | THCA |
| GOBP_REGULATION OF LEUKOCYTE MEDIATED CYTOTOXICITY | -0.638467454 | THCA |
| GOBP_PHOSPHATIDYLINOSITOL 3 KINASE SIGNALING | -0.428003793 | THCA |
| GOBP_REGULATION OF ALPHA BETA T CELL ACTIVATION | -0.41196058 | THCA |
| GOBP_3 UTR MEDIATED MRNA STABILIZATION | -0.840536172 | THYM |
| GOBP_INTERMEDIATE FILAMENT BASED PROCESS | -0.648274345 | THYM |
| GOBP_INTERMEDIATE FILAMENT ORGANIZATION | -0.666651656 | THYM |
| GOBP_REGULATION OF MRNA SPLICING VIA SPLICEOSOME | -0.678233763 | THYM |
| GOBP_REGULATION OF CELLULAR RESPONSE TO GROWTH FACTOR STIMULUS | 0.467605594 | THYM |
| GOBP_NATURAL KILLER CELL ACTIVATION INVOLVED IN IMMUNE RESPONSE | -0.728517137 | THYM |
| GOBP_REGULATION OF CANONICAL WNT SIGNALING PATHWAY | 0.49266432 | THYM |
| GOBP_PROTEOGLYCAN METABOLIC PROCESS | 0.525767788 | THYM |
| GOBP_TRANSFORMING GROWTH FACTOR BETA RECEPTOR SIGNALING PATHWAY | 0.455559572 | THYM |
| GOBP_REGULATION OF APOPTOTIC DNA FRAGMENTATION | -0.902121816 | THYM |
| GOCC_TRANSPORTER COMPLEX | 0.532972664 | UCEC |
| GOCC_ION CHANNEL COMPLEX | 0.552065384 | UCEC |
| GOBP_T CELL ACTIVATION INVOLVED IN IMMUNE RESPONSE | -0.563782146 | UCEC |
| GOBP_3 UTR MEDIATED MRNA STABILIZATION | 0.725296269 | UCEC |
| GOBP_POSITIVE REGULATION OF TRANSLATIONAL INITIATION | 0.766160149 | UCEC |
| GOBP_INTERMEDIATE FILAMENT BASED PROCESS | -0.469455768 | UCEC |
| GOBP_INTERMEDIATE FILAMENT ORGANIZATION | -0.502690129 | UCEC |
| GOBP_NEUTROPHIL CHEMOTAXIS | -0.500573879 | UCEC |
| GOBP_ADENYLATE CYCLASE ACTIVATING G PROTEIN COUPLED RECEPTOR SIGNALING PATHWAY | 0.519240498 | UCEC |
| GOBP_REGULATION OF ALPHA BETA T CELL ACTIVATION | -0.477816042 | UCEC |
| GOBP_T CELL ACTIVATION INVOLVED IN IMMUNE RESPONSE | -0.695489868 | UCS |
| GOBP_NEUTROPHIL MIGRATION | -0.629807575 | UCS |
| GOBP_RESPONSE TO CHEMOKINE | -0.685992117 | UCS |
| GOBP_NEUTROPHIL CHEMOTAXIS | -0.660885867 | UCS |
| GOBP_POSITIVE REGULATION OF T CELL PROLIFERATION | -0.645676429 | UCS |
| GOBP_GRANULOCYTE CHEMOTAXIS | -0.619708245 | UCS |
| GOBP_GRANULOCYTE MIGRATION | -0.602457663 | UCS |
| GOBP_ALPHA BETA T CELL ACTIVATION | -0.662763919 | UCS |
| GOBP_REGULATION OF ALPHA BETA T CELL ACTIVATION | -0.657846832 | UCS |
| GOBP_LIPID CATABOLIC PROCESS | -0.449039949 | UCS |
| GOCC_TRANSPORTER COMPLEX | 0.616270463 | UVM |
| GOCC_ION CHANNEL COMPLEX | 0.644284528 | UVM |
| GOBP_CELL ACTIVATION INVOLVED IN IMMUNE RESPONSE | 0.515329318 | UVM |
| GOCC_INTERMEDIATE FILAMENT | 0.544808077 | UVM |
| GOBP_INTERMEDIATE FILAMENT BASED PROCESS | 0.680085243 | UVM |
| GOBP_NEUTROPHIL MIGRATION | 0.626698471 | UVM |
| GOBP_NEUTROPHIL CHEMOTAXIS | 0.635565138 | UVM |
| GOBP_RESPONSE TO CHEMOKINE | 0.64106771 | UVM |
| GOBP_IMMUNE RESPONSE REGULATING CELL SURFACE RECEPTOR SIGNALING PATHWAY | 0.557471968 | UVM |
| GOBP_POSITIVE REGULATION OF T CELL PROLIFERATION | 0.58848224 | UVM |

**Abbreviations:** ACC: adrenocortical cancer; BLCA: bladder urothelial carcinoma; BRCA: breast invasive carcinoma; CESC: cervical & endocervical cancer; CHOL: cholangiocarcinoma; COAD: colon adenocarcinoma; DLBC: diffuse large B-cell lymphoma; ESCA: esophageal carcinoma; GBM glioblastoma multiforme; GO: Gene Ontology; GSEA: Gene Set Enrichment; HNSC: head & neck squamous cell carcinoma; KICH kidney chromophobe; KIRC: kidney clear cell carcinoma; KIRP: kidney papillary cell carcinoma; LAML: acute myeloid leukemia; LGG: brain lower grade glioma; LIHC: liver hepatocellular carcinoma; LUAD: lung adenocarcinoma; LUSC: lung squamous cell carcinoma; MESO: mesothelioma; MHC: major histocompatibility complex; OV: ovarian serous cystadenocarcinoma; PAAD: pancreatic adenocarcinoma; PCPG: pheochromocytoma & paraganglioma; PRAD: prostate adenocarcinoma; READ: rectum adenocarcinoma; SARC: sarcoma; SKCM: skin cutaneous melanoma; STAD: stomach adenocarcinoma; TGCT: testicular germ cell tumor; THCA thyroid carcinoma; THYM: thymoma; UCEC: uterine corpus endometrioid carcinoma; UCS uterine carcinosarcoma; UVM: uveal melanoma.
