# Supplementary material for: Immunotherapy, prognostic, and tumor biomarker based on pancancer analysis, SMARCD3
Source: Aging (Albany NY). 2024 Jun 11;16(11):10074–107. doi: 10.18632/aging.205921 (PMC11210247; doi:10.18632/aging.205921)
Supplement: Supplementary Table 7 [file aging-16-205921-s004.docx]

**Supplementary Table 7. All KEGG database GSEA enriched pathways.**

| **ID** | **Enrichment Score** | **Cancer** |
| --- | --- | --- |
| KEGG_PENTOSE AND GLUCURONATE INTERCONVERSIONS | -0.822015619 | ACC |
| KEGG_CHEMOKINE SIGNALING PATHWAY | -0.628756605 | ACC |
| KEGG_PORPHYRIN AND CHLOROPHYLL METABOLISM | -0.776195501 | ACC |
| KEGG_ASCORBATE AND ALDARATE METABOLISM | -0.82384476 | ACC |
| KEGG_METABOLISM OF XENOBIOTICS BY CYTOCHROME P450 | -0.695055364 | ACC |
| KEGG_LEISHMANIA INFECTION | -0.68497251 | ACC |
| KEGG_CYTOKINE CYTOKINE RECEPTOR INTERACTION | -0.543540641 | ACC |
| KEGG_OLFACTORY TRANSDUCTION | -0.450299112 | ACC |
| KEGG_TYROSINE METABOLISM | -0.734933541 | ACC |
| KEGG_STARCH AND SUCROSE METABOLISM | -0.706477471 | ACC |
| KEGG_DRUG METABOLISM CYTOCHROME P450 | -0.642609376 | ACC |
| KEGG_FATTY ACID METABOLISM | -0.658125036 | ACC |
| KEGG_GRAFT VERSUS HOST DISEASE | -0.678913861 | ACC |
| KEGG_NEUROACTIVE LIGAND RECEPTOR INTERACTION | 0.457500274 | ACC |
| KEGG_REGULATION OF ACTIN CYTOSKELETON | -0.433797759 | ACC |
| KEGG_DILATED CARDIOMYOPATHY | 0.645506783 | BLCA |
| KEGG_HYPERTROPHIC CARDIOMYOPATHY HCM | 0.627812052 | BLCA |
| KEGG_OLFACTORY TRANSDUCTION | 0.561214255 | BLCA |
| KEGG_CALCIUM SIGNALING PATHWAY | 0.56735118 | BLCA |
| KEGG_NEUROACTIVE LIGAND RECEPTOR INTERACTION | 0.535731362 | BLCA |
| KEGG_CARDIAC MUSCLE CONTRACTION | 0.651897637 | BLCA |
| KEGG_MATURITY ONSET DIABETES OF THE YOUNG | -0.683727205 | BLCA |
| KEGG_CYTOSOLIC DNA SENSING PATHWAY | -0.564587369 | BLCA |
| KEGG_ARRHYTHMOGENIC RIGHT VENTRICULAR CARDIOMYOPATHY ARVC | 0.635861943 | BLCA |
| KEGG_ADIPOCYTOKINE SIGNALING PATHWAY | 0.588612336 | BLCA |
| KEGG_VIRAL MYOCARDITIS | 0.570281529 | BLCA |
| KEGG_OLFACTORY TRANSDUCTION | -0.791698667 | BRCA |
| KEGG_ANTIGEN PROCESSING AND PRESENTATION | -0.762907351 | BRCA |
| KEGG_REGULATION OF AUTOPHAGY | -0.787092025 | BRCA |
| KEGG_RIG I LIKE RECEPTOR SIGNALING PATHWAY | -0.657859006 | BRCA |
| KEGG_CYTOSOLIC DNA SENSING PATHWAY | -0.708370814 | BRCA |
| KEGG_SYSTEMIC LUPUS ERYTHEMATOSUS | -0.599988475 | BRCA |
| KEGG_AUTOIMMUNE THYROID DISEASE | -0.709013095 | BRCA |
| KEGG_NATURAL KILLER CELL MEDIATED CYTOTOXICITY | -0.59034115 | BRCA |
| KEGG_TOLL LIKE RECEPTOR SIGNALING PATHWAY | -0.595761268 | BRCA |
| KEGG_PORPHYRIN AND CHLOROPHYLL METABOLISM | -0.683237929 | BRCA |
| KEGG_GRAFT VERSUS HOST DISEASE | -0.679240635 | BRCA |
| KEGG_JAK STAT SIGNALING PATHWAY | -0.538914634 | BRCA |
| KEGG_STARCH AND SUCROSE METABOLISM | -0.597006353 | BRCA |
| KEGG_TASTE TRANSDUCTION | -0.560816085 | BRCA |
| KEGG_NEUROACTIVE LIGAND RECEPTOR INTERACTION | 0.398871853 | BRCA |
| KEGG_MAPK SIGNALING PATHWAY | 0.394082054 | BRCA |
| KEGG_PROXIMAL TUBULE BICARBONATE RECLAMATION | 0.698770105 | BRCA |
| KEGG_ASCORBATE AND ALDARATE METABOLISM | -0.729644642 | BRCA |
| KEGG_NEUROACTIVE LIGAND RECEPTOR INTERACTION | 0.623812752 | CESC |
| KEGG_PROXIMAL TUBULE BICARBONATE RECLAMATION | 0.763282415 | CESC |
| KEGG_DILATED CARDIOMYOPATHY | 0.643764689 | CESC |
| KEGG_ARRHYTHMOGENIC RIGHT VENTRICULAR CARDIOMYOPATHY ARVC | 0.645261023 | CESC |
| KEGG_GAP JUNCTION | 0.635397853 | CESC |
| KEGG_HYPERTROPHIC CARDIOMYOPATHY HCM | 0.629379671 | CESC |
| KEGG_CALCIUM SIGNALING PATHWAY | 0.590326248 | CESC |
| KEGG_CARDIAC MUSCLE CONTRACTION | 0.630767037 | CESC |
| KEGG_OOCYTE MEIOSIS | 0.606935398 | CESC |
| KEGG_OLFACTORY TRANSDUCTION | 0.528362548 | CESC |
| KEGG_LONG TERM DEPRESSION | 0.627451465 | CESC |
| KEGG_LONG TERM POTENTIATION | 0.626719544 | CESC |
| KEGG_ALDOSTERONE REGULATED SODIUM REABSORPTION | 0.654692631 | CESC |
| KEGG_SYSTEMIC LUPUS ERYTHEMATOSUS | -0.721694132 | CHOL |
| KEGG_DRUG METABOLISM OTHER ENZYMES | -0.799369563 | CHOL |
| KEGG_DRUG METABOLISM CYTOCHROME P450 | -0.769755933 | CHOL |
| KEGG_COMPLEMENT AND COAGULATION CASCADES | -0.750688737 | CHOL |
| KEGG_RETINOL METABOLISM | -0.742062418 | CHOL |
| KEGG_METABOLISM OF XENOBIOTICS BY CYTOCHROME P450 | -0.765914383 | CHOL |
| KEGG_PORPHYRIN AND CHLOROPHYLL METABOLISM | -0.766731337 | CHOL |
| KEGG_VALINE LEUCINE AND ISOLEUCINE BIOSYNTHESIS | -0.907087809 | CHOL |
| KEGG_OLFACTORY TRANSDUCTION | -0.504779828 | CHOL |
| KEGG_NEUROACTIVE LIGAND RECEPTOR INTERACTION | 0.478154379 | CHOL |
| KEGG_NEUROACTIVE LIGAND RECEPTOR INTERACTION | 0.640212064 | COAD |
| KEGG_CELL ADHESION MOLECULES CAMS | 0.650302514 | COAD |
| KEGG_CALCIUM SIGNALING PATHWAY | 0.622292471 | COAD |
| KEGG_CARDIAC MUSCLE CONTRACTION | 0.654253548 | COAD |
| KEGG_HEDGEHOG SIGNALING PATHWAY | 0.66572079 | COAD |
| KEGG_DILATED CARDIOMYOPATHY | 0.642392293 | COAD |
| KEGG_ECM RECEPTOR INTERACTION | 0.641218649 | COAD |
| KEGG_HEMATOPOIETIC CELL LINEAGE | 0.634438126 | COAD |
| KEGG_CHEMOKINE SIGNALING PATHWAY | 0.59433486 | COAD |
| KEGG_TIGHT JUNCTION | 0.600937179 | COAD |
| KEGG_VASCULAR SMOOTH MUSCLE CONTRACTION | 0.608140363 | COAD |
| KEGG_FOCAL ADHESION | 0.577135437 | COAD |
| KEGG_LEUKOCYTE TRANSENDOTHELIAL MIGRATION | 0.59968885 | COAD |
| KEGG_PATHWAYS IN CANCER | 0.541535019 | COAD |
| KEGG_HYPERTROPHIC CARDIOMYOPATHY HCM | 0.634243556 | COAD |
| KEGG_B CELL RECEPTOR SIGNALING PATHWAY | 0.626073741 | COAD |
| KEGG_CYTOKINE CYTOKINE RECEPTOR INTERACTION | 0.551864276 | COAD |
| KEGG_OLFACTORY TRANSDUCTION | 0.824618981 | DLBC |
| KEGG_AUTOIMMUNE THYROID DISEASE | 0.772430615 | DLBC |
| KEGG_REGULATION OF AUTOPHAGY | 0.77823393 | DLBC |
| KEGG_RIG I LIKE RECEPTOR SIGNALING PATHWAY | 0.735393972 | DLBC |
| KEGG_ANTIGEN PROCESSING AND PRESENTATION | 0.721149159 | DLBC |
| KEGG_OLFACTORY TRANSDUCTION | 0.685253344 | ESCA |
| KEGG_PROTEASOME | 0.690783436 | ESCA |
| KEGG_DILATED CARDIOMYOPATHY | 0.619488615 | ESCA |
| KEGG_MELANOMA | 0.627232472 | ESCA |
| KEGG_CARDIAC MUSCLE CONTRACTION | 0.620427873 | ESCA |
| KEGG_HYPERTROPHIC CARDIOMYOPATHY HCM | 0.61573506 | ESCA |
| KEGG_HEDGEHOG SIGNALING PATHWAY | 0.62303849 | ESCA |
| KEGG_CALCIUM SIGNALING PATHWAY | 0.578705378 | ESCA |
| KEGG_VASCULAR SMOOTH MUSCLE CONTRACTION | 0.558534627 | ESCA |
| KEGG_CELL ADHESION MOLECULES CAMS | 0.553707678 | ESCA |
| KEGG_NEUROACTIVE LIGAND RECEPTOR INTERACTION | 0.536454858 | ESCA |
| KEGG_REGULATION OF ACTIN CYTOSKELETON | 0.534385721 | ESCA |
| KEGG_FOCAL ADHESION | 0.532431627 | ESCA |
| KEGG_MAPK SIGNALING PATHWAY | 0.495870735 | ESCA |
| KEGG_BASAL CELL CARCINOMA | 0.634848835 | ESCA |
| KEGG_STEROID HORMONE BIOSYNTHESIS | 0.625874829 | ESCA |
| KEGG_PORPHYRIN AND CHLOROPHYLL METABOLISM | 0.619716602 | ESCA |
| KEGG_PATHWAYS IN CANCER | 0.501631556 | ESCA |
| KEGG_ARRHYTHMOGENIC RIGHT VENTRICULAR CARDIOMYOPATHY ARVC | 0.592917386 | ESCA |
| KEGG_ECM RECEPTOR INTERACTION | 0.564490872 | ESCA |
| KEGG_CYTOKINE CYTOKINE RECEPTOR INTERACTION | -0.648370264 | GBM |
| KEGG_CHEMOKINE SIGNALING PATHWAY | -0.593175003 | GBM |
| KEGG_JAK STAT SIGNALING PATHWAY | -0.587382253 | GBM |
| KEGG_OLFACTORY TRANSDUCTION | -0.561641085 | GBM |
| KEGG_ASCORBATE AND ALDARATE METABOLISM | -0.799829535 | GBM |
| KEGG_PORPHYRIN AND CHLOROPHYLL METABOLISM | -0.748177091 | GBM |
| KEGG_PENTOSE AND GLUCURONATE INTERCONVERSIONS | -0.779223421 | GBM |
| KEGG_RENIN ANGIOTENSIN SYSTEM | -0.758373686 | GBM |
| KEGG_HEMATOPOIETIC CELL LINEAGE | -0.626178781 | GBM |
| KEGG_METABOLISM OF XENOBIOTICS BY CYTOCHROME P450 | -0.674814594 | GBM |
| KEGG_CARDIAC MUSCLE CONTRACTION | 0.722738072 | HNSC |
| KEGG_ARRHYTHMOGENIC RIGHT VENTRICULAR CARDIOMYOPATHY ARVC | 0.67673946 | HNSC |
| KEGG_HYPERTROPHIC CARDIOMYOPATHY HCM | 0.665133194 | HNSC |
| KEGG_DILATED CARDIOMYOPATHY | 0.658124 | HNSC |
| KEGG_MELANOMA | 0.664799978 | HNSC |
| KEGG_NEUROACTIVE LIGAND RECEPTOR INTERACTION | 0.630120647 | HNSC |
| KEGG_CALCIUM SIGNALING PATHWAY | 0.622454264 | HNSC |
| KEGG_TIGHT JUNCTION | 0.593384708 | HNSC |
| KEGG_MAPK SIGNALING PATHWAY | 0.552855989 | HNSC |
| KEGG_VIRAL MYOCARDITIS | 0.640941977 | HNSC |
| KEGG_PURINE METABOLISM | 0.575644631 | HNSC |
| KEGG_LONG TERM POTENTIATION | 0.621294371 | HNSC |
| KEGG_MTOR SIGNALING PATHWAY | 0.616778803 | HNSC |
| KEGG_PROXIMAL TUBULE BICARBONATE RECLAMATION | 0.678628823 | HNSC |
| KEGG_GLYCOSAMINOGLYCAN BIOSYNTHESIS HEPARAN SULFATE | 0.669495418 | HNSC |
| KEGG_PORPHYRIN AND CHLOROPHYLL METABOLISM | 0.639139091 | HNSC |
| KEGG_REGULATION OF ACTIN CYTOSKELETON | 0.54859454 | HNSC |
| KEGG_OLFACTORY TRANSDUCTION | 0.517460362 | HNSC |
| KEGG_FOLATE BIOSYNTHESIS | -0.89487132 | KICH |
| KEGG_OLFACTORY TRANSDUCTION | 0.755916034 | KIRC |
| KEGG_NEUROACTIVE LIGAND RECEPTOR INTERACTION | 0.622111001 | KIRC |
| KEGG_VIBRIO CHOLERAE INFECTION | 0.669109277 | KIRC |
| KEGG_RETINOL METABOLISM | -0.701104165 | KIRP |
| KEGG_PPAR SIGNALING PATHWAY | -0.660209719 | KIRP |
| KEGG_DRUG METABOLISM CYTOCHROME P450 | -0.647124062 | KIRP |
| KEGG_PENTOSE AND GLUCURONATE INTERCONVERSIONS | -0.747674992 | KIRP |
| KEGG_METABOLISM OF XENOBIOTICS BY CYTOCHROME P450 | -0.645307579 | KIRP |
| KEGG_COMPLEMENT AND COAGULATION CASCADES | -0.626474234 | KIRP |
| KEGG_ASCORBATE AND ALDARATE METABOLISM | -0.76438347 | KIRP |
| KEGG_PROXIMAL TUBULE BICARBONATE RECLAMATION | -0.738888718 | KIRP |
| KEGG_OLFACTORY TRANSDUCTION | -0.45857938 | KIRP |
| KEGG_PORPHYRIN AND CHLOROPHYLL METABOLISM | -0.705279745 | KIRP |
| KEGG_DRUG METABOLISM OTHER ENZYMES | -0.678023506 | KIRP |
| KEGG_STARCH AND SUCROSE METABOLISM | -0.632406225 | KIRP |
| KEGG_NEUROACTIVE LIGAND RECEPTOR INTERACTION | -0.45825398 | KIRP |
| KEGG_CALCIUM SIGNALING PATHWAY | -0.473534298 | KIRP |
| KEGG_SYSTEMIC LUPUS ERYTHEMATOSUS | -0.457550812 | KIRP |
| KEGG_AUTOIMMUNE THYROID DISEASE | -0.630003906 | LAML |
| KEGG_RENIN ANGIOTENSIN SYSTEM | -0.727245835 | LAML |
| KEGG_OLFACTORY TRANSDUCTION | -0.486483601 | LAML |
| KEGG_CYTOSOLIC DNA SENSING PATHWAY | -0.602815342 | LAML |
| KEGG_REGULATION OF AUTOPHAGY | -0.62876958 | LAML |
| KEGG_NOD LIKE RECEPTOR SIGNALING PATHWAY | 0.56574347 | LAML |
| KEGG_RIG I LIKE RECEPTOR SIGNALING PATHWAY | -0.585210461 | LAML |
| KEGG_NEUROACTIVE LIGAND RECEPTOR INTERACTION | -0.446195498 | LAML |
| KEGG_CHEMOKINE SIGNALING PATHWAY | -0.614791845 | LGG |
| KEGG_CYTOKINE CYTOKINE RECEPTOR INTERACTION | -0.574950523 | LGG |
| KEGG_PENTOSE AND GLUCURONATE INTERCONVERSIONS | 0.774523396 | LGG |
| KEGG_ASCORBATE AND ALDARATE METABOLISM | 0.806827929 | LGG |
| KEGG_HEMATOPOIETIC CELL LINEAGE | -0.616732249 | LGG |
| KEGG_CELL ADHESION MOLECULES CAMS | -0.599419687 | LGG |
| KEGG_T CELL RECEPTOR SIGNALING PATHWAY | -0.605838163 | LGG |
| KEGG_RETINOL METABOLISM | 0.688344945 | LGG |
| KEGG_STARCH AND SUCROSE METABOLISM | 0.688102009 | LGG |
| KEGG_PORPHYRIN AND CHLOROPHYLL METABOLISM | 0.722179397 | LGG |
| KEGG_DRUG METABOLISM CYTOCHROME P450 | 0.641829897 | LGG |
| KEGG_METABOLISM OF XENOBIOTICS BY CYTOCHROME P450 | 0.666332892 | LGG |
| KEGG_DRUG METABOLISM OTHER ENZYMES | 0.63544854 | LGG |
| KEGG_RIBOSOME | 0.558182628 | LGG |
| KEGG_STEROID HORMONE BIOSYNTHESIS | 0.622476301 | LGG |
| KEGG_OLFACTORY TRANSDUCTION | -0.42857793 | LGG |
| KEGG_CARDIAC MUSCLE CONTRACTION | 0.510167475 | LGG |
| KEGG_TYPE I DIABETES MELLITUS | -0.709696808 | LGG |
| KEGG_ALLOGRAFT REJECTION | -0.711708154 | LGG |
| KEGG_GRAFT VERSUS HOST DISEASE | -0.777497074 | LGG |
| KEGG_NOD LIKE RECEPTOR SIGNALING PATHWAY | -0.566551993 | LGG |
| KEGG_INTESTINAL IMMUNE NETWORK FOR IGA PRODUCTION | -0.654576579 | LGG |
| KEGG_O GLYCAN BIOSYNTHESIS | 0.714032571 | LIHC |
| KEGG_HYPERTROPHIC CARDIOMYOPATHY HCM | 0.632780656 | LIHC |
| KEGG_DILATED CARDIOMYOPATHY | 0.613927931 | LIHC |
| KEGG_NEUROACTIVE LIGAND RECEPTOR INTERACTION | 0.585211012 | LIHC |
| KEGG_CYTOKINE CYTOKINE RECEPTOR INTERACTION | 0.569751131 | LIHC |
| KEGG_HEDGEHOG SIGNALING PATHWAY | 0.641741171 | LIHC |
| KEGG_GLYCOSPHINGOLIPID BIOSYNTHESIS LACTO AND NEOLACTO SERIES | 0.687313749 | LIHC |
| KEGG_BASAL CELL CARCINOMA | 0.620113653 | LIHC |
| KEGG_AXON GUIDANCE | 0.574422186 | LIHC |
| KEGG_CARDIAC MUSCLE CONTRACTION | 0.587987521 | LIHC |
| KEGG_JAK STAT SIGNALING PATHWAY | 0.560429711 | LIHC |
| KEGG_CALCIUM SIGNALING PATHWAY | 0.545170274 | LIHC |
| KEGG_OLFACTORY TRANSDUCTION | -0.750313866 | LUAD |
| KEGG_ASCORBATE AND ALDARATE METABOLISM | -0.808780696 | LUAD |
| KEGG_PORPHYRIN AND CHLOROPHYLL METABOLISM | -0.771721423 | LUAD |
| KEGG_STEROID HORMONE BIOSYNTHESIS | -0.704755804 | LUAD |
| KEGG_CELL CYCLE | -0.586174559 | LUAD |
| KEGG_ALPHA LINOLENIC ACID METABOLISM | 0.705347549 | LUAD |
| KEGG_RETINOL METABOLISM | -0.602384306 | LUAD |
| KEGG_PENTOSE AND GLUCURONATE INTERCONVERSIONS | -0.768414944 | LUAD |
| KEGG_METABOLISM OF XENOBIOTICS BY CYTOCHROME P450 | -0.60153821 | LUAD |
| KEGG_OLFACTORY TRANSDUCTION | -0.871854215 | LUSC |
| KEGG_CYTOSOLIC DNA SENSING PATHWAY | -0.807762249 | LUSC |
| KEGG_RIG I LIKE RECEPTOR SIGNALING PATHWAY | -0.768789071 | LUSC |
| KEGG_CARDIAC MUSCLE CONTRACTION | 0.670954114 | LUSC |
| KEGG_DILATED CARDIOMYOPATHY | 0.652846352 | LUSC |
| KEGG_REGULATION OF AUTOPHAGY | -0.823508423 | LUSC |
| KEGG_HYPERTROPHIC CARDIOMYOPATHY HCM | 0.65258522 | LUSC |
| KEGG_ABC TRANSPORTERS | 0.670177459 | LUSC |
| KEGG_CALCIUM SIGNALING PATHWAY | 0.537840367 | LUSC |
| KEGG_ANTIGEN PROCESSING AND PRESENTATION | -0.663665015 | LUSC |
| KEGG_ARRHYTHMOGENIC RIGHT VENTRICULAR CARDIOMYOPATHY ARVC | 0.607435565 | LUSC |
| KEGG_LONG TERM DEPRESSION | 0.577511449 | LUSC |
| KEGG_HEDGEHOG SIGNALING PATHWAY | 0.593687599 | LUSC |
| KEGG_FOCAL ADHESION | 0.484538452 | LUSC |
| KEGG_TOLL LIKE RECEPTOR SIGNALING PATHWAY | -0.625330537 | LUSC |
| KEGG_AUTOIMMUNE THYROID DISEASE | -0.645269269 | LUSC |
| KEGG_VIRAL MYOCARDITIS | 0.527812421 | LUSC |
| KEGG_INTESTINAL IMMUNE NETWORK FOR IGA PRODUCTION | 0.573789123 | LUSC |
| KEGG_TIGHT JUNCTION | 0.489997484 | LUSC |
| KEGG_BASAL CELL CARCINOMA | 0.535723949 | LUSC |
| KEGG_AXON GUIDANCE | 0.472042302 | LUSC |
| KEGG_NATURAL KILLER CELL MEDIATED CYTOTOXICITY | -0.530949122 | LUSC |
| KEGG_ECM RECEPTOR INTERACTION | 0.487252147 | LUSC |
| KEGG_MELANOGENESIS | 0.484380475 | LUSC |
| KEGG_LONG TERM POTENTIATION | 0.477764066 | LUSC |
| KEGG_CHEMOKINE SIGNALING PATHWAY | 0.402079602 | LUSC |
| KEGG_MAPK SIGNALING PATHWAY | 0.422309754 | LUSC |
| KEGG_PATHWAYS IN CANCER | 0.378386234 | LUSC |
| KEGG_AUTOIMMUNE THYROID DISEASE | -0.611084025 | MESO |
| KEGG_INTESTINAL IMMUNE NETWORK FOR IGA PRODUCTION | -0.575459036 | MESO |
| KEGG_CITRATE CYCLE TCA CYCLE | 0.73338583 | MESO |
| KEGG_GLYCOSAMINOGLYCAN DEGRADATION | 0.771736055 | MESO |
| KEGG_HYPERTROPHIC CARDIOMYOPATHY HCM | 0.658105134 | MESO |
| KEGG_NEUROACTIVE LIGAND RECEPTOR INTERACTION | 0.616159162 | MESO |
| KEGG_ASCORBATE AND ALDARATE METABOLISM | -0.891092212 | OV |
| KEGG_PENTOSE AND GLUCURONATE INTERCONVERSIONS | -0.873266348 | OV |
| KEGG_PORPHYRIN AND CHLOROPHYLL METABOLISM | -0.831695312 | OV |
| KEGG_STARCH AND SUCROSE METABOLISM | -0.743535274 | OV |
| KEGG_STEROID HORMONE BIOSYNTHESIS | -0.73241228 | OV |
| KEGG_MATURITY ONSET DIABETES OF THE YOUNG | -0.811273529 | OV |
| KEGG_DRUG METABOLISM OTHER ENZYMES | -0.725818212 | OV |
| KEGG_METABOLISM OF XENOBIOTICS BY CYTOCHROME P450 | -0.685818755 | OV |
| KEGG_RETINOL METABOLISM | -0.647522107 | OV |
| KEGG_DRUG METABOLISM CYTOCHROME P450 | -0.627545948 | OV |
| KEGG_NEUROACTIVE LIGAND RECEPTOR INTERACTION | -0.54060432 | OV |
| KEGG_GRAFT VERSUS HOST DISEASE | 0.771259326 | OV |
| KEGG_REGULATION OF AUTOPHAGY | 0.767155152 | OV |
| KEGG_ASTHMA | 0.738601195 | OV |
| KEGG_ALLOGRAFT REJECTION | 0.644696558 | OV |
| KEGG_TYPE I DIABETES MELLITUS | 0.549474291 | OV |
| KEGG_CARDIAC MUSCLE CONTRACTION | -0.565795963 | OV |
| KEGG_GLUTATHIONE METABOLISM | -0.666045422 | PAAD |
| KEGG_REGULATION OF AUTOPHAGY | -0.672572388 | PAAD |
| KEGG_ANTIGEN PROCESSING AND PRESENTATION | -0.590829391 | PAAD |
| KEGG_CYTOSOLIC DNA SENSING PATHWAY | -0.654175158 | PAAD |
| KEGG_CELL CYCLE | -0.529148498 | PAAD |
| KEGG_NEUROACTIVE LIGAND RECEPTOR INTERACTION | 0.586998552 | PAAD |
| KEGG_DNA REPLICATION | -0.634054837 | PAAD |
| KEGG_HOMOLOGOUS RECOMBINATION | -0.643450323 | PAAD |
| KEGG_RIG I LIKE RECEPTOR SIGNALING PATHWAY | -0.589164354 | PAAD |
| KEGG_TOLL LIKE RECEPTOR SIGNALING PATHWAY | -0.510258989 | PAAD |
| KEGG_SYSTEMIC LUPUS ERYTHEMATOSUS | -0.481426646 | PAAD |
| KEGG_TYPE II DIABETES MELLITUS | 0.646388768 | PAAD |
| KEGG_OLFACTORY TRANSDUCTION | 0.523690041 | PAAD |
| KEGG_OTHER GLYCAN DEGRADATION | -0.682502427 | PAAD |
| KEGG_NATURAL KILLER CELL MEDIATED CYTOTOXICITY | -0.441315258 | PAAD |
| KEGG_CALCIUM SIGNALING PATHWAY | 0.531696751 | PAAD |
| KEGG_CITRATE CYCLE TCA CYCLE | -0.759483591 | PCPG |
| KEGG_OTHER GLYCAN DEGRADATION | -0.85941779 | PCPG |
| KEGG_GRAFT VERSUS HOST DISEASE | -0.671124707 | PCPG |
| KEGG_PORPHYRIN AND CHLOROPHYLL METABOLISM | -0.629180852 | PCPG |
| KEGG_PYRUVATE METABOLISM | -0.600325359 | PCPG |
| KEGG_OLFACTORY TRANSDUCTION | 0.528241892 | PCPG |
| KEGG_REGULATION OF AUTOPHAGY | 0.880027888 | PRAD |
| KEGG_RIG I LIKE RECEPTOR SIGNALING PATHWAY | 0.775027575 | PRAD |
| KEGG_CYTOSOLIC DNA SENSING PATHWAY | 0.741474576 | PRAD |
| KEGG_CYTOKINE CYTOKINE RECEPTOR INTERACTION | 0.655894696 | PRAD |
| KEGG_AUTOIMMUNE THYROID DISEASE | 0.685100863 | PRAD |
| KEGG_JAK STAT SIGNALING PATHWAY | 0.637110299 | PRAD |
| KEGG_ANTIGEN PROCESSING AND PRESENTATION | 0.648404681 | PRAD |
| KEGG_TOLL LIKE RECEPTOR SIGNALING PATHWAY | 0.633760869 | PRAD |
| KEGG_PRIMARY IMMUNODEFICIENCY | 0.67599475 | PRAD |
| KEGG_HEMATOPOIETIC CELL LINEAGE | 0.620170768 | PRAD |
| KEGG_COMPLEMENT AND COAGULATION CASCADES | 0.638403819 | PRAD |
| KEGG_CHEMOKINE SIGNALING PATHWAY | 0.589871345 | PRAD |
| KEGG_CELL ADHESION MOLECULES CAMS | 0.591180767 | PRAD |
| KEGG_CARDIAC MUSCLE CONTRACTION | 0.605041316 | PRAD |
| KEGG_NATURAL KILLER CELL MEDIATED CYTOTOXICITY | 0.587596967 | PRAD |
| KEGG_OLFACTORY TRANSDUCTION | 0.567205181 | PRAD |
| KEGG_HYPERTROPHIC CARDIOMYOPATHY HCM | 0.593129417 | PRAD |
| KEGG_DILATED CARDIOMYOPATHY | 0.586069851 | PRAD |
| KEGG_CALCIUM SIGNALING PATHWAY | 0.563716507 | PRAD |
| KEGG_NEUROACTIVE LIGAND RECEPTOR INTERACTION | 0.525387736 | PRAD |
| KEGG_TIGHT JUNCTION | 0.532107314 | PRAD |
| KEGG_METABOLISM OF XENOBIOTICS BY CYTOCHROME P450 | 0.607994271 | PRAD |
| KEGG_INTESTINAL IMMUNE NETWORK FOR IGA PRODUCTION | 0.644402702 | PRAD |
| KEGG_NOD LIKE RECEPTOR SIGNALING PATHWAY | 0.610373219 | PRAD |
| KEGG_DRUG METABOLISM CYTOCHROME P450 | 0.599469754 | PRAD |
| KEGG_LEUKOCYTE TRANSENDOTHELIAL MIGRATION | 0.54995972 | PRAD |
| KEGG_ASTHMA | 0.646349663 | PRAD |
| KEGG_TASTE TRANSDUCTION | 0.592641119 | PRAD |
| KEGG_MELANOGENESIS | 0.547021822 | PRAD |
| KEGG_CELL ADHESION MOLECULES CAMS | 0.650239493 | READ |
| KEGG_COMPLEMENT AND COAGULATION CASCADES | 0.694366946 | READ |
| KEGG_CYTOKINE CYTOKINE RECEPTOR INTERACTION | 0.604169008 | READ |
| KEGG_FOCAL ADHESION | 0.605293939 | READ |
| KEGG_NEUROACTIVE LIGAND RECEPTOR INTERACTION | 0.574928153 | READ |
| KEGG_CALCIUM SIGNALING PATHWAY | 0.599644363 | READ |
| KEGG_CHEMOKINE SIGNALING PATHWAY | 0.593094155 | READ |
| KEGG_FATTY ACID METABOLISM | 0.728888608 | READ |
| KEGG_AXON GUIDANCE | 0.613144025 | READ |
| KEGG_ECM RECEPTOR INTERACTION | 0.644178712 | READ |
| KEGG_LEUKOCYTE TRANSENDOTHELIAL MIGRATION | 0.59567042 | READ |
| KEGG_RIG I LIKE RECEPTOR SIGNALING PATHWAY | 0.628364494 | READ |
| KEGG_TGF BETA SIGNALING PATHWAY | 0.612447594 | READ |
| KEGG_ARRHYTHMOGENIC RIGHT VENTRICULAR CARDIOMYOPATHY ARVC | 0.620889899 | READ |
| KEGG_LEISHMANIA INFECTION | 0.62094472 | READ |
| KEGG_INTESTINAL IMMUNE NETWORK FOR IGA PRODUCTION | 0.64672886 | READ |
| KEGG_JAK STAT SIGNALING PATHWAY | 0.558869621 | READ |
| KEGG_ERBB SIGNALING PATHWAY | 0.595509854 | READ |
| KEGG_PATHWAYS IN CANCER | 0.516644213 | READ |
| KEGG_HEMATOPOIETIC CELL LINEAGE | 0.582608409 | READ |
| KEGG_GAP JUNCTION | 0.579781999 | READ |
| KEGG_REGULATION OF ACTIN CYTOSKELETON | 0.514815866 | READ |
| KEGG_FOLATE BIOSYNTHESIS | 0.856421503 | SARC |
| KEGG_CALCIUM SIGNALING PATHWAY | 0.575103181 | SARC |
| KEGG_VASCULAR SMOOTH MUSCLE CONTRACTION | 0.575602431 | SARC |
| KEGG_TYPE II DIABETES MELLITUS | 0.644833721 | SARC |
| KEGG_INSULIN SIGNALING PATHWAY | 0.554157309 | SARC |
| KEGG_GNRH SIGNALING PATHWAY | 0.54587647 | SARC |
| KEGG_TIGHT JUNCTION | 0.524009186 | SARC |
| KEGG_NEUROACTIVE LIGAND RECEPTOR INTERACTION | 0.517929361 | SARC |
| KEGG_TYROSINE METABOLISM | 0.636657186 | SARC |
| KEGG_STARCH AND SUCROSE METABOLISM | 0.607750695 | SARC |
| KEGG_LONG TERM POTENTIATION | 0.54863197 | SARC |
| KEGG_DRUG METABOLISM CYTOCHROME P450 | 0.552139945 | SARC |
| KEGG_MAPK SIGNALING PATHWAY | 0.474683683 | SARC |
| KEGG_GRAFT VERSUS HOST DISEASE | -0.783774951 | SARC |
| KEGG_ALLOGRAFT REJECTION | -0.694014475 | SARC |
| KEGG_TYPE I DIABETES MELLITUS | -0.603488314 | SARC |
| KEGG_PRION DISEASES | -0.57881898 | SARC |
| KEGG_PRIMARY IMMUNODEFICIENCY | -0.547466128 | SARC |
| KEGG_DILATED CARDIOMYOPATHY | 0.514117452 | SARC |
| KEGG_LONG TERM DEPRESSION | 0.519147437 | SARC |
| KEGG_MELANOMA | 0.509683921 | SARC |
| KEGG_PRIMARY IMMUNODEFICIENCY | -0.774927981 | SKCM |
| KEGG_B CELL RECEPTOR SIGNALING PATHWAY | -0.623552867 | SKCM |
| KEGG_GRAFT VERSUS HOST DISEASE | -0.697496375 | SKCM |
| KEGG_TASTE TRANSDUCTION | 0.703699893 | SKCM |
| KEGG_MATURITY ONSET DIABETES OF THE YOUNG | 0.755345109 | SKCM |
| KEGG_GALACTOSE METABOLISM | 0.742891137 | SKCM |
| KEGG_CYTOKINE CYTOKINE RECEPTOR INTERACTION | -0.500966325 | SKCM |
| KEGG_STEROID HORMONE BIOSYNTHESIS | 0.678677129 | SKCM |
| KEGG_COMPLEMENT AND COAGULATION CASCADES | 0.639853515 | SKCM |
| KEGG_HEMATOPOIETIC CELL LINEAGE | -0.567361055 | SKCM |
| KEGG_ANTIGEN PROCESSING AND PRESENTATION | -0.5454921 | SKCM |
| KEGG_NATURAL KILLER CELL MEDIATED CYTOTOXICITY | -0.5024274 | SKCM |
| KEGG_NEUROACTIVE LIGAND RECEPTOR INTERACTION | 0.507237027 | SKCM |
| KEGG_JAK STAT SIGNALING PATHWAY | -0.42553094 | SKCM |
| KEGG_CHEMOKINE SIGNALING PATHWAY | -0.423225067 | SKCM |
| KEGG_INTESTINAL IMMUNE NETWORK FOR IGA PRODUCTION | -0.633136406 | SKCM |
| KEGG_CITRATE CYCLE TCA CYCLE | 0.654874351 | SKCM |
| KEGG_DRUG METABOLISM CYTOCHROME P450 | 0.585166479 | SKCM |
| KEGG_METABOLISM OF XENOBIOTICS BY CYTOCHROME P450 | 0.58699379 | SKCM |
| KEGG_AMYOTROPHIC LATERAL SCLEROSIS ALS | 0.688573864 | STAD |
| KEGG_DILATED CARDIOMYOPATHY | 0.665596835 | STAD |
| KEGG_ARRHYTHMOGENIC RIGHT VENTRICULAR CARDIOMYOPATHY ARVC | 0.676303025 | STAD |
| KEGG_HYPERTROPHIC CARDIOMYOPATHY HCM | 0.661945689 | STAD |
| KEGG_NEUROACTIVE LIGAND RECEPTOR INTERACTION | 0.645570893 | STAD |
| KEGG_ADIPOCYTOKINE SIGNALING PATHWAY | 0.646406843 | STAD |
| KEGG_CALCIUM SIGNALING PATHWAY | 0.621676937 | STAD |
| KEGG_CARDIAC MUSCLE CONTRACTION | 0.639185591 | STAD |
| KEGG_BASAL CELL CARCINOMA | 0.624287612 | STAD |
| KEGG_AXON GUIDANCE | 0.568857689 | STAD |
| KEGG_CELL ADHESION MOLECULES CAMS | 0.557487138 | STAD |
| KEGG_MAPK SIGNALING PATHWAY | 0.518789563 | STAD |
| KEGG_COMPLEMENT AND COAGULATION CASCADES | 0.584882627 | STAD |
| KEGG_ECM RECEPTOR INTERACTION | 0.569932232 | STAD |
| KEGG_VASCULAR SMOOTH MUSCLE CONTRACTION | 0.548175299 | STAD |
| KEGG_FOCAL ADHESION | 0.527320107 | STAD |
| KEGG_OLFACTORY TRANSDUCTION | 0.495305071 | STAD |
| KEGG_STEROID HORMONE BIOSYNTHESIS | 0.773172131 | TGCT |
| KEGG_RETINOL METABOLISM | 0.7488136 | TGCT |
| KEGG_PENTOSE AND GLUCURONATE INTERCONVERSIONS | 0.806327757 | TGCT |
| KEGG_METABOLISM OF XENOBIOTICS BY CYTOCHROME P450 | 0.720059558 | TGCT |
| KEGG_DRUG METABOLISM CYTOCHROME P450 | 0.719950048 | TGCT |
| KEGG_DRUG METABOLISM OTHER ENZYMES | 0.738681077 | TGCT |
| KEGG_ASCORBATE AND ALDARATE METABOLISM | 0.79702449 | TGCT |
| KEGG_PORPHYRIN AND CHLOROPHYLL METABOLISM | 0.749114449 | TGCT |
| KEGG_STARCH AND SUCROSE METABOLISM | 0.718632278 | TGCT |
| KEGG_COMPLEMENT AND COAGULATION CASCADES | 0.690316778 | TGCT |
| KEGG_NEUROACTIVE LIGAND RECEPTOR INTERACTION | 0.618798486 | TGCT |
| KEGG_VASCULAR SMOOTH MUSCLE CONTRACTION | 0.602785711 | TGCT |
| KEGG_MATURITY ONSET DIABETES OF THE YOUNG | 0.790286403 | TGCT |
| KEGG_TYROSINE METABOLISM | 0.697944867 | TGCT |
| KEGG_LINOLEIC ACID METABOLISM | 0.705628521 | TGCT |
| KEGG_PPAR SIGNALING PATHWAY | 0.637047306 | TGCT |
| KEGG_CALCIUM SIGNALING PATHWAY | 0.580177353 | TGCT |
| KEGG_ARACHIDONIC ACID METABOLISM | 0.660802521 | TGCT |
| KEGG_RENIN ANGIOTENSIN SYSTEM | 0.742409836 | TGCT |
| KEGG_GLYCOLYSIS GLUCONEOGENESIS | 0.630206161 | TGCT |
| KEGG_TGF BETA SIGNALING PATHWAY | 0.603580758 | TGCT |
| KEGG_FOCAL ADHESION | 0.55979698 | TGCT |
| KEGG_HYPERTROPHIC CARDIOMYOPATHY HCM | 0.597416693 | TGCT |
| KEGG_AXON GUIDANCE | 0.571795622 | TGCT |
| KEGG_PRIMARY BILE ACID BIOSYNTHESIS | 0.713547561 | TGCT |
| KEGG_OLFACTORY TRANSDUCTION | 0.709732876 | THCA |
| KEGG_REGULATION OF AUTOPHAGY | 0.77132247 | THCA |
| KEGG_MATURITY ONSET DIABETES OF THE YOUNG | 0.765163097 | THCA |
| KEGG_VIRAL MYOCARDITIS | -0.573068478 | THCA |
| KEGG_ECM RECEPTOR INTERACTION | -0.525935811 | THCA |
| KEGG_ARACHIDONIC ACID METABOLISM | 0.638695024 | THCA |
| KEGG_CYTOSOLIC DNA SENSING PATHWAY | 0.645528191 | THCA |
| KEGG_HEMATOPOIETIC CELL LINEAGE | -0.521906343 | THCA |
| KEGG_ANTIGEN PROCESSING AND PRESENTATION | 0.621670053 | THCA |
| KEGG_RIG I LIKE RECEPTOR SIGNALING PATHWAY | 0.587269209 | THCA |
| KEGG_AUTOIMMUNE THYROID DISEASE | 0.583573482 | THCA |
| KEGG_ALLOGRAFT REJECTION | -0.577849816 | THCA |
| KEGG_TYROSINE METABOLISM | -0.564194518 | THCA |
| KEGG_LEISHMANIA INFECTION | -0.486425888 | THCA |
| KEGG_OXIDATIVE PHOSPHORYLATION | 0.497114351 | THCA |
| KEGG_PRIMARY IMMUNODEFICIENCY | -0.552669519 | THCA |
| KEGG_SYSTEMIC LUPUS ERYTHEMATOSUS | 0.476685272 | THCA |
| KEGG_NOD LIKE RECEPTOR SIGNALING PATHWAY | -0.45509689 | THCA |
| KEGG_GRAFT VERSUS HOST DISEASE | -0.603297748 | THCA |
| KEGG_STEROID HORMONE BIOSYNTHESIS | 0.542486201 | THCA |
| KEGG_GLYCEROLIPID METABOLISM | 0.563645681 | THCA |
| KEGG_CHEMOKINE SIGNALING PATHWAY | -0.525894169 | THCA |
| KEGG_CELL ADHESION MOLECULES CAMS | -0.53864832 | THCA |
| KEGG_LINOLEIC ACID METABOLISM | 0.647049275 | THCA |
| KEGG_ASTHMA | -0.590540656 | THCA |
| KEGG_RIBOSOME | 0.52612328 | THCA |
| KEGG_CALCIUM SIGNALING PATHWAY | -0.404611005 | THCA |
| KEGG_RETINOL METABOLISM | 0.517099918 | THCA |
| KEGG_FOCAL ADHESION | -0.365675826 | THCA |
| KEGG_PENTOSE AND GLUCURONATE INTERCONVERSIONS | -0.838450817 | THYM |
| KEGG_ASCORBATE AND ALDARATE METABOLISM | -0.849145351 | THYM |
| KEGG_REGULATION OF AUTOPHAGY | -0.788995263 | THYM |
| KEGG_PORPHYRIN AND CHLOROPHYLL METABOLISM | -0.762755405 | THYM |
| KEGG_CYTOSOLIC DNA SENSING PATHWAY | -0.730527266 | THYM |
| KEGG_STARCH AND SUCROSE METABOLISM | -0.726894082 | THYM |
| KEGG_DRUG METABOLISM OTHER ENZYMES | -0.719580928 | THYM |
| KEGG_RIG I LIKE RECEPTOR SIGNALING PATHWAY | -0.670071685 | THYM |
| KEGG_TOLL LIKE RECEPTOR SIGNALING PATHWAY | -0.602138016 | THYM |
| KEGG_METABOLISM OF XENOBIOTICS BY CYTOCHROME P450 | -0.636351225 | THYM |
| KEGG_TGF BETA SIGNALING PATHWAY | 0.600201251 | THYM |
| KEGG_JAK STAT SIGNALING PATHWAY | -0.56416157 | THYM |
| KEGG_FOCAL ADHESION | 0.510588457 | THYM |
| KEGG_MELANOMA | 0.575708213 | THYM |
| KEGG_ECM RECEPTOR INTERACTION | 0.568796346 | THYM |
| KEGG_OLFACTORY TRANSDUCTION | -0.487846358 | THYM |
| KEGG_ANTIGEN PROCESSING AND PRESENTATION | -0.668928239 | UCEC |
| KEGG_REGULATION OF AUTOPHAGY | -0.694769906 | UCEC |
| KEGG_CYTOSOLIC DNA SENSING PATHWAY | -0.641675097 | UCEC |
| KEGG_CARDIAC MUSCLE CONTRACTION | 0.691786746 | UCEC |
| KEGG_FATTY ACID METABOLISM | -0.631697305 | UCEC |
| KEGG_RIG I LIKE RECEPTOR SIGNALING PATHWAY | -0.585325519 | UCEC |
| KEGG_GRAFT VERSUS HOST DISEASE | -0.619537223 | UCEC |
| KEGG_TASTE TRANSDUCTION | -0.581788906 | UCEC |
| KEGG_ASTHMA | -0.60814552 | UCEC |
| KEGG_AUTOIMMUNE THYROID DISEASE | -0.578797872 | UCEC |
| KEGG_PORPHYRIN AND CHLOROPHYLL METABOLISM | 0.702016647 | UCEC |
| KEGG_TOLL LIKE RECEPTOR SIGNALING PATHWAY | -0.499449615 | UCEC |
| KEGG_RIBOFLAVIN METABOLISM | -0.641371642 | UCEC |
| KEGG_GLYCOLYSIS GLUCONEOGENESIS | -0.525464644 | UCEC |
| KEGG_JAK STAT SIGNALING PATHWAY | -0.468656685 | UCEC |
| KEGG_AXON GUIDANCE | 0.545672252 | UCEC |
| KEGG_ALLOGRAFT REJECTION | -0.560701421 | UCEC |
| KEGG_GLYCOSPHINGOLIPID BIOSYNTHESIS LACTO AND NEOLACTO SERIES | -0.584829022 | UCEC |
| KEGG_NEUROACTIVE LIGAND RECEPTOR INTERACTION | 0.49539455 | UCEC |
| KEGG_LEISHMANIA INFECTION | -0.496006288 | UCEC |
| KEGG_PROXIMAL TUBULE BICARBONATE RECLAMATION | 0.729892494 | UCEC |
| KEGG_ASCORBATE AND ALDARATE METABOLISM | 0.72365924 | UCEC |
| KEGG_T CELL RECEPTOR SIGNALING PATHWAY | -0.479194524 | UCEC |
| KEGG_FC EPSILON RI SIGNALING PATHWAY | -0.469432698 | UCEC |
| KEGG_HEMATOPOIETIC CELL LINEAGE | -0.445857006 | UCEC |
| KEGG_NATURAL KILLER CELL MEDIATED CYTOTOXICITY | -0.5569456 | UCEC |
| KEGG_CYTOKINE CYTOKINE RECEPTOR INTERACTION | -0.659033498 | UCS |
| KEGG_REGULATION OF AUTOPHAGY | -0.833602617 | UCS |
| KEGG_CARDIAC MUSCLE CONTRACTION | 0.665058991 | UCS |
| KEGG_PORPHYRIN AND CHLOROPHYLL METABOLISM | -0.810356484 | UCS |
| KEGG_ANTIGEN PROCESSING AND PRESENTATION | -0.73691983 | UCS |
| KEGG_CYTOSOLIC DNA SENSING PATHWAY | -0.757436532 | UCS |
| KEGG_NATURAL KILLER CELL MEDIATED CYTOTOXICITY | -0.699125506 | UCS |
| KEGG_METABOLISM OF XENOBIOTICS BY CYTOCHROME P450 | -0.734296365 | UCS |
| KEGG_RETINOL METABOLISM | -0.762040978 | UCS |
| KEGG_RIG I LIKE RECEPTOR SIGNALING PATHWAY | -0.717889205 | UCS |
| KEGG_TOLL LIKE RECEPTOR SIGNALING PATHWAY | -0.692703123 | UCS |
| KEGG_AUTOIMMUNE THYROID DISEASE | -0.750824968 | UCS |
| KEGG_T CELL RECEPTOR SIGNALING PATHWAY | -0.66477103 | UCS |
| KEGG_DRUG METABOLISM CYTOCHROME P450 | -0.701242003 | UCS |
| KEGG_RIBOSOME | 0.597004373 | UCS |
| KEGG_DRUG METABOLISM OTHER ENZYMES | -0.729955881 | UCS |
| KEGG_JAK STAT SIGNALING PATHWAY | -0.603981319 | UCS |
| KEGG_CHEMOKINE SIGNALING PATHWAY | -0.579074155 | UCS |
| KEGG_HYPERTROPHIC CARDIOMYOPATHY HCM | 0.575959824 | UCS |
| KEGG_CELL ADHESION MOLECULES CAMS | -0.586029487 | UCS |
| KEGG_OLFACTORY TRANSDUCTION | -0.479951671 | UCS |
| KEGG_DILATED CARDIOMYOPATHY | 0.539410906 | UCS |
| KEGG_PENTOSE AND GLUCURONATE INTERCONVERSIONS | -0.810075518 | UCS |
| KEGG_ASCORBATE AND ALDARATE METABOLISM | -0.84773112 | UCS |
| KEGG_GRAFT VERSUS HOST DISEASE | -0.725304228 | UCS |
| KEGG_B CELL RECEPTOR SIGNALING PATHWAY | -0.601818827 | UCS |
| KEGG_TYPE I DIABETES MELLITUS | -0.690726426 | UCS |
| KEGG_TASTE TRANSDUCTION | 0.583678224 | UCS |
| KEGG_NEUROACTIVE LIGAND RECEPTOR INTERACTION | 0.637005153 | UVM |
| KEGG_CARDIAC MUSCLE CONTRACTION | 0.67409409 | UVM |
| KEGG_T CELL RECEPTOR SIGNALING PATHWAY | 0.654212393 | UVM |
| KEGG_CYTOKINE CYTOKINE RECEPTOR INTERACTION | 0.616893797 | UVM |
| KEGG_HYPERTROPHIC CARDIOMYOPATHY HCM | 0.652106289 | UVM |
| KEGG_DILATED CARDIOMYOPATHY | 0.641722484 | UVM |
| KEGG_HEMATOPOIETIC CELL LINEAGE | 0.644551486 | UVM |
| KEGG_CALCIUM SIGNALING PATHWAY | 0.597674221 | UVM |
| KEGG_CHEMOKINE SIGNALING PATHWAY | 0.588845023 | UVM |
| KEGG_JAK STAT SIGNALING PATHWAY | 0.5763049 | UVM |
| KEGG_NOD LIKE RECEPTOR SIGNALING PATHWAY | 0.638848988 | UVM |
| KEGG_ARRHYTHMOGENIC RIGHT VENTRICULAR CARDIOMYOPATHY ARVC | 0.622740944 | UVM |

**Abbreviations:** ACC: adrenocortical cancer; BLCA: bladder urothelial carcinoma; BRCA: breast invasive carcinoma; CESC: cervical & endocervical cancer; CHOL: cholangiocarcinoma; COAD: colon adenocarcinoma; DLBC: diffuse large B-cell lymphoma; ESCA: esophageal carcinoma; GBM glioblastoma multiforme; GSEA: Gene Set Enrichment; HNSC: head & neck squamous cell carcinoma; KEGG: Kyoto Encyclopedia of Genes and Genomes; KICH kidney chromophobe; KIRC: kidney clear cell carcinoma; KIRP: kidney papillary cell carcinoma; LAML: acute myeloid leukemia; LGG: brain lower grade glioma; LIHC: liver hepatocellular carcinoma; LUAD: lung adenocarcinoma; LUSC: lung squamous cell carcinoma; MESO: mesothelioma; MHC: major histocompatibility complex; OV: ovarian serous cystadenocarcinoma; PAAD: pancreatic adenocarcinoma; PCPG: pheochromocytoma & paraganglioma; PRAD: prostate adenocarcinoma; READ: rectum adenocarcinoma; SARC: sarcoma; SKCM: skin cutaneous melanoma; STAD: stomach adenocarcinoma; TGCT: testicular germ cell tumor; THCA thyroid carcinoma; THYM: thymoma; UCEC: uterine corpus endometrioid carcinoma; UCS uterine carcinosarcoma; UVM: uveal melanoma.
